# Interactions between timing and transmissibility explain diverse flavivirus dynamics in Fiji

**DOI:** 10.1038/s41467-021-21788-y

**Published:** 2021-03-15

**Authors:** Alasdair D. Henderson, Mike Kama, Maite Aubry, Stephane Hue, Anita Teissier, Taina Naivalu, Vinaisi D. Bechu, Jimaima Kailawadoko, Isireli Rabukawaqa, Aalisha Sahukhan, Martin L. Hibberd, Eric J. Nilles, Sebastian Funk, Jimmy Whitworth, Conall H. Watson, Colleen L. Lau, W. John Edmunds, Van-Mai Cao-Lormeau, Adam J. Kucharski

**Affiliations:** 1 Centre for the Mathematical Modelling of Infectious Diseases, Department of Infectious Disease Epidemiology, London School of Hygiene & Tropical Medicine, London, UK; 2 Fiji Center for Diseases Control, Suva, Fiji; 3 Institut Louis Malardé, Papeete, Tahiti French Polynesia; 4 Fiji National University, Suva, Fiji; 5 Department of Infection Biology, London School of Hygiene & Tropical Medicine, London, UK; 6 Harvard Humanitarian Initiative, Cambridge, MA USA; 7 Epidemic Diseases Research Group Oxford, University of Oxford, Oxford, UK; 8 Research School of Population Health, The Australian National University, Canberra, ACT Australia

**Keywords:** Dengue virus, Bayesian inference, Computer modelling, Epidemiology

## Abstract

Zika virus (ZIKV) has caused large, brief outbreaks in isolated populations, however ZIKV can also persist at low levels over multiple years. The reasons for these diverse transmission dynamics remain poorly understood. In Fiji, which has experienced multiple large single-season dengue epidemics, there was evidence of multi-year transmission of ZIKV between 2013 and 2017. To identify factors that could explain these differences in dynamics between closely related mosquito-borne flaviviruses, we jointly fit a transmission dynamic model to surveillance, serological and molecular data. We estimate that the observed dynamics of ZIKV were the result of two key factors: strong seasonal effects, which created an ecologically optimal time of year for outbreaks; and introduction of ZIKV after this optimal time, which allowed ZIKV transmission to persist over multiple seasons. The ability to jointly fit to multiple data sources could help identify a similar range of possible outbreak dynamics in other settings.

## Abstract (web summary)

Dengue and Zika virus are closely related flaviviruses but can have contrasting transmission dynamics in the same populations. Here, the authors use a model combining serological, surveillance and viral sequence data to explain differences in transmission dynamics in Fiji.

## Introduction

Emerging and re-emerging flaviviruses typically generate large, brief outbreaks, particularly in isolated island populations^1–3^. However, alongside examples of clearly-defined outbreaks of Zika virus (ZIKV) with a high attack rate during 2014–16^3–6^, there is evidence of low-level, multi-year circulation^7^. The challenges of collecting data during an emerging outbreak mean that the reasons for these diverse flavivirus dynamics are currently not well understood.

**Fig. 1:**
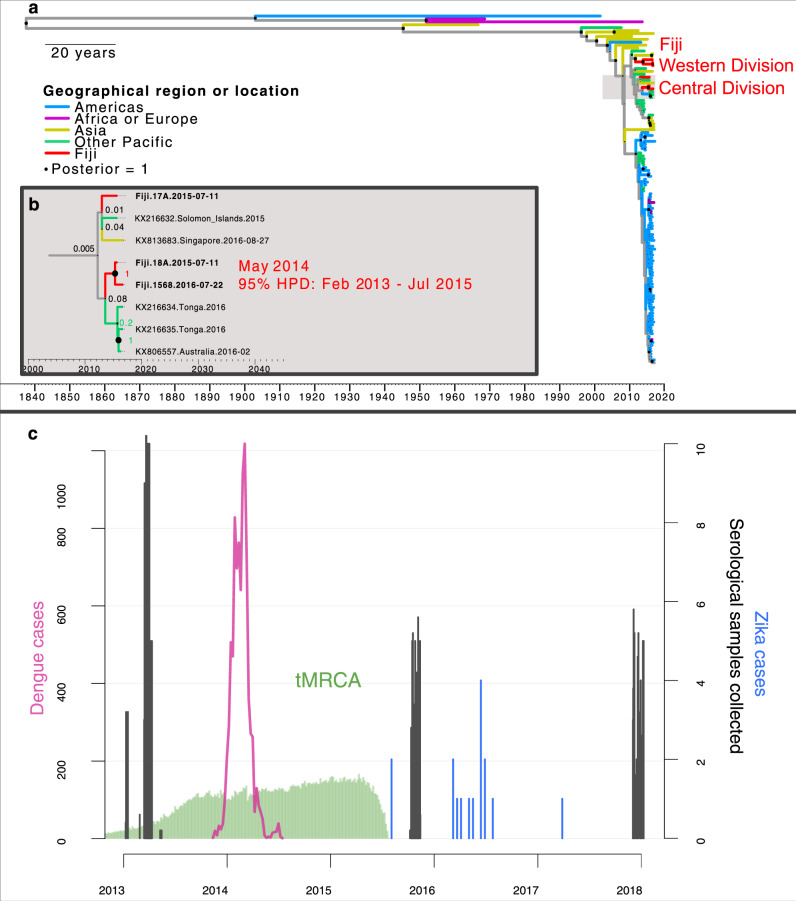
Available data on ZIKV transmission in Fiji. **a** Dated Bayesian phylogeny of three sequences recovered from Central Division, Fiji, and other locations in the Pacific and Americas. Nodes with a posterior probability of 1.00 are indicated. Branch lengths correspond to time in calendar years. (inset **b**) Detailed phylogeny of the Central Division cluster. The estimated time to most recent common ancestor (tMRCA) for the two closely related Central Division sequences, selected based on previous analysis^10^, is shown. **c** Green region, density of estimated tMRCA from phylogenetic analysis. This distribution was used as a prior for ZIKV introduction time in the main transmission model fitting. Pink line, cases of DENV3. Blue bars, cases of ZIKV. Grey bars, serological samples collected in Central Division.

To investigate which factors shape the invasion dynamics of dengue virus (DENV) and ZIKV, we combined surveillance, serological and molecular data to analyse the emergence of ZIKV and re-emergence of DENV-3 in the same population in Fiji, which resulted in very different outbreak dynamics. In 2013/14, dengue virus serotype 3 (DENV-3) caused a large outbreak that lasted 9 months, with 12,413 suspected cases reported in Central Division which has a population of 342,000^8,9^. In contrast, there were only 16 PCR-confirmed cases of the closely related flavivirus ZIKV in Fiji between 2015 and 2017, with evidence of low-level circulation over multiple seasons^10^. Data from a longitudinal community serological survey found an increase from 7.8% seroprevalence for long-term ZIKV antibodies in November 2013 to 21.9% in November 2015^10^, suggesting that ZIKV had been circulating during this period despite only two confirmed cases being reported. Further, the estimated time to most recent common ancestor (tMRCA) estimated in phylogenetic analysis of available ZIKV gene sequences—including from 3 ZIKV cases from Central Division, Fiji—suggested that ZIKV may have been introduced into Fiji in late 2013 or 2014^10^ (Fig. 1).

There are several possible explanations for differences in flavivirus dynamics in the same population. One possible explanation is that ZIKV was less transmissible than DENV in Fiji: analysis of flavivirus outbreaks on other Pacific islands found that ZIKV can have a slightly lower basic reproduction number, *R*_0_, than DENV in the same location^11,12^. Another factor is seasonality: because mosquito populations are influenced by environmental factors like temperature and rainfall^13,14^ there is a strong temporal component to flavivirus transmission in Fiji^8^; the time of year the virus is introduced therefore could influence the dynamics of the resulting outbreak. Additionally, flavivirus outbreak dynamics will depend on prior immunity within the population, as well as immunity that accumulates during an outbreak^8,11,15^, or wanes following infection^16^. Finally, the tMRCA of ZIKV in Fiji spans the duration of a large DENV-3 outbreak (Fig. 1c), so it is possible that infections during the DENV-3 outbreak also conferred a degree of transient cross-protection against other flaviviruses^17–19^.

Both ZIKV and DENV can cause asymptomatic or sub-clinical infections^20,21^, which means many infections will not appear in routine surveillance data. We therefore developed a transmission dynamic model and jointly fitted it to surveillance data, three longitudinal serological surveys and virus sequences to estimate unobserved ZIKV infection dynamics in Fiji during 2013–17. We used this model to identify factors that could explain why the dynamics of ZIKV and DENV-3 were so different.

## Results

### ZIKV arrived later than DENV and persisted for multiple years

**Fig. 2:**
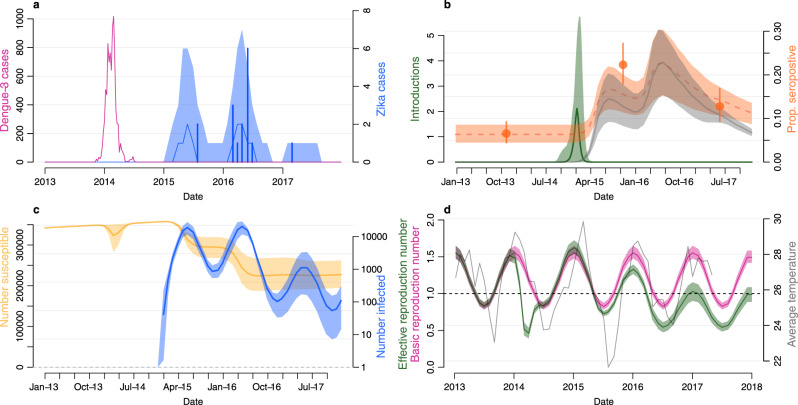
Estimated transmission of ZIKV in Fiji using a mathematical model and multiple data sources. **a** Pink line, weekly cases of DENV-3. Blue bars, monthly cases of ZIKV. Blue dashed line and region, model estimated median cases of ZIKV and 95% CrI. **b** Seroprevalence and introduction of ZIKV. Green line and region, estimated median introduction of ZIKV infected individuals and 95% CrI. Grey line and region, estimated median proportion of the population that had recovered and were temporarily immune to ZIKV infection (median and 95% CrI). Orange dashed line and region, estimated observed seroprevalence and 95% CrI. Seroprevalence includes an estimated 6.3% (95% CrI: 4.4--8.5%) false positive rate and 79% (95% CrI: 52--98%) assay sensitivity. Orange dots and vertical lines, observed ZIKV seroprevalence from three serological surveys (mean and 95% binomial CI, *n* = 458, 327, 321 in 2013, 2015, 2017, respectively). **c** ZIKV infection dynamics in Central Division. Yellow line and region, median and 95% CrI of the number of people susceptible to ZIKV. Blue line and region, median and 95% CrI of the number infected on the natural log scale. **d** Pink line and region, estimated basic reproduction number for ZIKV (median and 95% CrI). Green line and region, effective reproduction number (median and 95% CrI). This included an estimated decline in transmission coinciding with a 2014 vector clean-up campaign^8^. Grey line, monthly temperature data from Suva, Central Division.

We fitted our full transmission model to serological and surveillance data and used prior information from an analysis of molecular data. We found it was possible to explain the observed dynamics with the following combination of factors: ZIKV was introduced into Central Division, Fiji, after the ecologically optimal time of year and transmitted at a low level over 3 years until a combination of seasonal forcing and accumulation of immunity resulted in the end of transmission in 2017 (Fig. 2a).

Although the first case of ZIKV was reported in July 2015, we found evidence that transmission of ZIKV likely began in early 2015 in Central Division, Fiji. Infectious individuals were introduced to our model using a continuous logistic function defined by parameters for the peak, width and midpoint of the wave of introductions. The 95% credible interval for the most likely midpoint ranges from October 2014 to February 2015 with a median of January 2015 (Fig. 2b and Supplementary Fig. S11). By using a posterior estimate from a previous phylogenetic analysis as a prior in our model, our joint inference produced a more precise estimate than the original phylogenetic analysis alone,^10^ which had an inferred introduction date of May 2014 (95% HPD: Feb 2013–Jul 2015).

### Seasonal variation in transmission defines a period of substantially higher risk for ZIKV introduction

To estimate the role of seasonal variation in temperature on transmission, the model included sinusoidal forcing in transmission with timing and amplitude estimated from available daily temperature data^22^. We then converted this into a relative transmission rate using the published data on the mechanistic relationship between temperature and basic reproduction number for transmission driven by *Aedes aegypti* mosquitoes^13^.

In Fiji, we found a strong seasonal variation in transmission which peaked in February (Fig. 2c). The seasonality of transmission resulted in a period with an effective reproduction number (*R*) below 1 (Fig. 2d). However, we estimated that this was insufficient for the epidemic to fade out over the colder months between 2015–2016 and 2016–2017 as the prevalent number of infections was consistently above 100 (Fig. 2c).

The seasonal pattern of transmission also created a period of heightened epidemic risk if a flavivirus was introduced during this period. Towards the end of the calendar year as temperatures, and therefore the transmission rate, increased the required number of initial cases to seed an outbreak was lower than during the colder months. We excluded the possibility of an outbreak emerging from an implausibly small introduction during the period when *R* was below 1.

### Estimated *R*_0_ and reporting proportion for ZIKV was lower than the DENV-3 outbreak

In our model, we estimated the full cycle basic reproduction number (*R*_0_), the average number of new infections in humans from an initial infected human, which varied over time according to seasonal forcing. Over the course of a year, we estimated a median ZIKV *R*_0_ of 1.2 (95% CrI: 0.8–1.5) (Fig. 2c and Supplementary Table S4). Before fitting to ZIKV data we initially fitted the same model to DENV-3 surveillance serological data from the 2013-14 epidemic (Supplementary Fig. S9 and Supplementary Table S4). From this analysis we estimated a higher but comparable median and 95% credible interval for *R*_0_ for the 2013-14 DENV-3 epidemic (1.8; 95% CrI: (1.3–2.4)).

Other studies have found evidence that inherent DENV transmissibility is similar or slightly higher than ZIKV in the same location^11,23–25^. A more complex modelling analysis of this 2013–14 DENV-3 epidemic estimated an *R*_0_ of 1.12 (95% CrI: 1.02–1.25), similar to our estimate of ZIKV for the same region^8^. Our results are consistent with these findings, that ZIKV is similarly but slightly less transmissible in the same population as DENV. This likely contributed, but was insufficient, to explain the diverse outbreak dynamics between DENV-3 and ZIKV in Central Division.

We also estimated a very small reporting proportion for ZIKV from our model of 0.01% (95% CrI: 0.006–0.02%). This implies that nearly all infections were not reported as cases and were either asymptomatic, not severe enough to seek medical attention, not referred for ZIKV tests by clinicians in Fiji or undetected ZIKV in tests. This low reporting proportion is uncommon for arbovirus outbreaks in Fiji. We estimated a reporting proportion of 16% (95% CrI: 12–23%) for DENV-3 during the 2013–14 epidemic. This discrepancy is the main cause of the diverse observed outbreaks in surveillance case data. However, it is insufficient to explain why ZIKV infections transmitted at a low level for multiple years.

### Flavivirus outbreak dynamics depend on the virus introduction timing

**Fig. 3:**
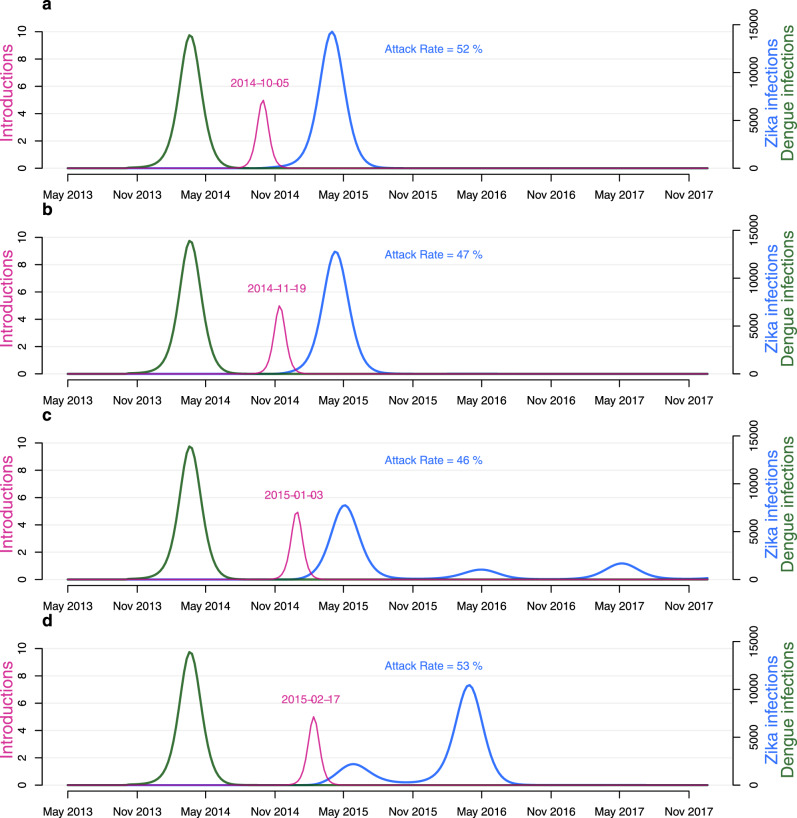
Transmission dynamics by varying introduction time. Simulated ZIKV outbreaks using the maximum a posteriori parameter set and adjusting the midpoint of the introduction of ZIKV infectious individuals. Changing the introduction time alone can vary resulting outbreak dynamics between low level circulation over multiple years to large single-season epidemics. The modelled DENV-3 infections during the 2013-14 epidemic is reproduced here for comparison. Introduction time centred around October 2014 (**a**), November 2014 (**b**), January 2015 (**c**), February 2015 (**d**). Blue line, model simulation for the prevalence of ZIKV infections (not cases). Green line, modelled DENV-3 2013–14 infections. Pink line, introduction of infectious individuals. The date of the midpoint of the introduction function is displayed in pink. The attack rate is equal to the sum of all ZIKV infections divided by the population size at the start of the outbreak, 342,000 people.

To examine how introduction dynamics could shape subsequent ZIKV outbreaks, we simulated model trajectories using the maximum a posteriori estimates, then varied the midpoint of the introduction function. We found that the timing of introductions had a large effect on ensuing outbreak dynamics (Fig. 3a–d). For example, using our model with an introduction event centred around January 2015—slightly after peak transmission—there were three waves of infections at a low level, as in our main findings (Fig. 3c). We found that shifting the introduction event 2 months earlier to November 2014—slightly before transmissibility had peaked—caused a larger single season outbreak comparable to the 2013-14 DENV-3 epidemic (Fig. 3b). An introduction centred around February 2015, generated a smaller first wave in a shorter high transmission season given the later introduction. However, this delayed the epidemic and there was a larger second wave in 2016 (Fig. 3c). In our model, varying the timing of the introductions alone could create diverse outbreak dynamics from single large outbreaks to seasonal annual persistence for multiple years.

### Early ZIKV introduction and interaction with DENV was not well supported by the data

It has been proposed that infection with one flavivirus may result in transient cross-immunity against others^17,26,27^. We therefore examined whether the large DENV-3 epidemic in 2013/14 could have temporary cross-immunity that delayed the emergence of ZIKV until 2015. Our model allowed a proportion of those infected with DENV-3 during the 2013/14 to potentially be temporarily protected from ZIKV infection. To ensure the outbreaks overlapped, we constrained ZIKV to be introduced in 2013 (i.e. before the DENV-3 outbreak). In this scenario, we found that a combination of DENV-3 cross-immunity and reduced transmission that coincided with a vector control campaign in March 2014 could have suppressed ZIKV transmission in 2014 (Supplementary Fig. S17). However the Deviance Information Criterion (DIC) from this model was much higher than our baseline model (Table 1), suggesting very little support for this alternative explanation. The reason for poor model performance is the short outbreak duration that resulted from this interaction: DENV did not just influence ZIKV in 2014 in this scenario; by suppressing ZIKV transmission to a large extent, subsequent multi-year outbreaks of ZIKV during 2015–2017 were not possible in the model, in contrast with the observed reported cases during this period.

**Table 1:** Model performance and estimated *R*_0_ when introduction date is unconstrained and constrained. The model with no constraint on introduction time estimated an early 2015 introduction, which meant there was no need for a DENV/ZIKV interaction to explain the observed data. In contrast, constraining the introduction time to 2013 resulted in an estimated interaction, but performed less well when compared to the observed serological and surveillance data.

Model		DIC	*R*_0_ (95% CrI)
A	No constraint on introduction	76.3	1.2 (0.8–1.5)
B	Forced 2013 introduction	129.8	1.2 (0.9–1.6)

## Discussion

We combined multiple data sources with a dynamic transmission model to reconstruct unobserved transmission dynamics of ZIKV in Fiji between 2013 and 2017. We found that transmission persisted over multiple years with three consecutive small annual outbreaks between 2015 and 2017, with strong seasonal forcing in transmission resulting in a high risk period of the year for ZIKV introduction. This means there is potential for large, brief flavivirus outbreaks, as well as a period of lower risk where low-level transmission is more likely. We estimated a mean basic reproduction number of 1.2 (95% CrI: 0.8–1.5) for ZIKV, which combined with seasonality in transmission meant there was insufficient infection—and hence acquired immunity—in 2015 or 2016 to prevent re-emergence of the virus in the following year.

We found that ZIKV was slightly less transmissible than DENV in the same population and that nearly all ZIKV infections were undetected, unlike the estimated reporting proportion during the DENV-3 epidemic. We show that if the ZIKV reporting proportion was equivalent to DENV-3 then the two epidemics would appear similar in overall magnitude (Supplementary Fig. S12). However, this is insufficient to explain why ZIKV transmitted at a low level over multiple years, unlike DENV-3 which caused a large single-season epidemic. We have demonstrated that small changes to the introduction time can produce a diverse range of outbreak dynamics because of the strong seasonal forcing in ZIKV transmission in Fiji (Fig. 3).

Our ability to infer unobserved dynamics benefited from being able to simultaneously fit a transmission dynamic model to serological, surveillance and viral sequence data. Each data source provided insights into different aspects of the dynamics. The surveillance data provided information on the temporal distribution of symptomatic infections, the serological surveys provided estimates of community-level exposure at different points in time and the sequence data provided informative prior information on the potential time of ZIKV introduction to Fiji. To synthesise these complementary information sources, we used a mathematical model that could generate observations representing the serological and surveillance data, then we jointly fitted the model to these data sets in a Bayesian framework, while the molecular data formed an informative prior on the time of introduction. The data available for Fiji presented a unique opportunity to compare and contrast the dynamics of ZIKV and DENV infection; without the combination of these three data sets it would have been far more challenging to reliably infer the unobserved ZIKV outbreak dynamics.

In our study we allowed flexibility in the timing and size of the initial virus introduction, but in all scenarios we assumed that there was a single introduction wave with continuous transmission afterwards, rather than multiple separate introductions in consecutive years. ZIKV outbreaks in other locations have shown evidence of multiple introductions^28^. Although we cannot rule out multiple early introductions that did not result in widespread transmission, a previous phylogenetic analysis of ZIKV sequences from the region identified two distinct clusters of Fiji sequences, one of which included sequences recovered from Western Division and the other from Central Division^10^. The weak branch support for the Central Division cluster in both analyses means we cannot distinguish between one or more introduction events. However, the fact that this previous analysis estimated a cluster that included all three Central Division sequences, with a close relationship between sequences from 2015 and 2016 suggests persistence rather than separate introductions. Single ZIKV introduction events have also been estimated for other Pacific Islands^29^, and an introduction during 2014–15 in Fiji is further supported by context of ZIKV transmission in the Pacific, where the majority of large outbreaks occurred in 2014 and early 2015, rather than 2016 onwards: the first large outbreak occurred in French Polynesia in late 2013; during 2014 there were ZIKV outbreaks confirmed in New Caledonia, Easter Island and the Cook Islands and in 2015 in Vanuatu, and Solomon Islands^29^. Moreover, the level of seroprevalence found in Fiji in 2015 suggests there was widespread transmission between 2013 and 2015, rather than a series of isolated cases^10^.

We used a deterministic framework to model ZIKV, which is limited because it could produce artificially cyclical outbreaks that do not reflect reality. There were two potential problems deterministic models can cause that we explicitly guarded against. With strong seasonal forcing in transmission it is possible that epidemics can reduce to implausibly low levels to persist over colder months before re-emerging. Our model included a condition that there was at least one infected person for the virus to transmit. Regardless, we estimated that the number infected over colder months was consistently above 100 during lower transmission seasons (Fig. 2c). Secondly, to avoid epidemics emerging in our model at implausible times of the year we explicitly prevented introductions and a possible epidemic take off when *R* was below 1 in our model.

In our model we assumed that detectable anti-ZIKV antibodies could wane over time and therefore seroprevalence in the population could decline over time, as has been observed in serological studies^16,30^. However, as the precise relationship between a specific titre value and susceptibility to ZIKV infection is unclear, we assumed that seroreversions did not lead to loss of protection. This is consistent with other ZIKV modelling studies^31,32^ and the fact that many participants in the Fiji survey who were seronegative for ZIKV (as measured by MIA) specific antibodies still had evidence of neutralising titres^16^.

In the Fiji serological survey, ZIKV seroprevalence was already 7.8% in November 2013. Given the antigenic similarity of DENV and ZIKV, we assumed that this level of ZIKV seroprevalence may be the result of cross-reactive antibody responses from prior flavivirus infections. To reflect this, we included a parameter that measured the false positive percentage (1 minus the specificity) of the assay, which was estimated as 6.3% (95% CrI: 4.4–8.5%) in the model fitting (Supplementary Table S4). This may explain why there was some evidence of seroprevalence before our model estimated ZIKV had arrived in Fiji. Similarly, we estimated a sensitivity of 79% (95% CrI: 52–98%). Both are consistent with the previously reported assay sensitivity and specificity for ZIKV of 79.6% and 94.9%^16^. With these adjustments we found that the observed seroprevalence was broadly consistent with our expected seroprevalence from the model. However, in 2015 the observed value was at the limit of our expected seroprevalence (Fig. 2b). It is possible the assay was more sensitive or less specific during this serological analysis. It is unlikely that there were more true infections than our model produced since this would require a higher transmission rate and therefore increase the likelihood of a single season large epidemic, which is inconsistent with the surveillance data.

The surveillance data collected during the 2013–14 DENV-3 epidemic was primarily from syndromic surveillance and did not have laboratory confirmation^8^. There is significant overlap in the definitions of dengue-like illness, Zika-like illness, Influenza-like illness, and acute fever and rash, so it’s a challenge for doctors and nurses to classify patients into these categories, and there are inherent uncertainties in the reported numbers. It is therefore possible that some of the cases defined as DENV-3 in 2013–14 were actually caused by ZIKV infection and ZIKV was introduced earlier to Fiji than we suggest here. We did not preclude this possible conclusion from the model, but the probability of ZIKV arriving in Fiji in 2013 unobserved and still circulating in 2017 was less plausible than a late 2014 introduction in our model (Supplementary Fig. S17 and Supplementary Table S5). All confirmed cases attributed to ZIKV in this study had reverse transcription PCR confirmation in Fiji^10^.

Despite these limitations, our results show that ZIKV does not necessarily cause large, brief outbreaks in settings where other flaviviruses have done so, and can persist over multiple seasons, mostly undetected, even in isolated locations. We found that these dynamics most likely resulted from the timing and the magnitude of the introductions of infections prior to the first reported cases. Given the strong seasonal forcing on transmission of vector-borne infections in Fiji, the timing of the introduction had a large impact on the resulting dynamics. This indicates a period of high epidemic risk in Fiji—specifically as temperatures begin to increase—during which surveillance should be particularly vigilant. It also suggests that a wide range of outbreak dynamics are possible if infections are introduced outside this period, including repeated, low-level outbreaks over varying numbers of years. By estimating this range of possible transmission dynamics with such models, it should be possible to develop improved forecasts about likely outbreak dynamics when new cases are identified. More broadly, with a similar joint analysis of wider data sources for flavivirus outbreaks, there is potential to characterise the range of possible dynamics for other settings as well.

## Methods

### Ethics statement

Each serosurvey had ethical approval from both the Fiji National Research Ethics Review Committee (2013-03, 2015.111.C.D and 2017.20.MC) and the London School of Hygiene & Tropical Medicine Observational Research Ethics Committee (6344, 10207 and 12037). All participants in follow-up studies in 2015 and 2017 had agreed to be recontacted for further health research and an updated informed consent was obtained. To respect local customs and ensure research activities were culturally accepted, the head of the household or village was visited with local bilingual field teams. The study was explained in English or iTaukei at the preference of the potential participant. Parental/guardian consent was obtained for children under 18.

### Surveillance data

Between June 2015 and August 2017 there were 16 confirmed cases of ZIKV through laboratory surveillance in Central Division, Fiji (Supplementary Figs. S1 and S2). At the Institut Louis Malardé (Tahiti, French Polynesia), reverse transcription polymerase chain reaction (RT-PCR) was used to detect ZIKV in referred cases. Between March 2016 and August 2017 these tests were performed on site at Mataika House, public health laboratory, Fiji Center for Diseases Control. In addition, saliva samples were submitted for ZIKV RT-PCR. Full details are described in the Supplementary Methods.

### Serological data

We conducted a longitudinal seroepidemiological survey over the period 2013–2017 in Central Division, Fiji. The original sampling used population-proportionate sampling in Central Division to identify nursing zones from across Fiji for inclusion and full details of the sampling framework have been published^33,34^. The same participants were followed up in 2015^8,10^ and 2017^16^ to collect another serum sample for testing.

In brief, serological testing of samples to detect immunoglobulin class G antibodies against ZIKV and each of the four DENV serotypes was performed using a recombinant antigen-based microsphere immunoassay (MIA). The sensitivity and specificity of the MIA assay were respectively 100% and 100% for DENV-1, 89.5% and 97.1% for DENV-2, 100% and 100% for DENV-3, 96.9% and 100% for DENV-4, and 79.6% and 94.9% for ZIKV^16^. A subset of samples in 2013 and 2015 and all samples in 2017 were also tested for the presence of neutralising antibodies against ZIKV and each of the four DENV serotypes using a plaque reduction neutralisation assay. Full details are available in the Supplementary Methods.

### Molecular data

A previous study details the recovery of the envelope (E) gene of ZIKV strains from Fiji and the original phylogenetic analysis that informed this study^10^. The sequences from Central Division were recovered from two saliva samples collected in 2015 and a serum sample collected in 2016 (Supplementary Tables S1 and S2). The same sequences retrieved from GenBank in the original study were used in this study, which included 120 ZIKV sequences all from the Asian lineage^10^.

### Modelling seasonal forcing using climate data

We collected daily maximum and minimum temperature data from the Fiji Meteorological Service which covered the study period up to June 2017. We defined a sinusoidal function with variable amplitude and midpoint. We estimated these two parameters by fitting this function to daily average temperature data over the study period by Bayesian Markov Chain Monte Carlo (MCMC) using a normal distribution and likelihood and uninformative priors. We then converted the estimated peak and base of this temperature wave function to ZIKV transmission using previously published data^13^. The distance between the relative peak and base *R*_0_ was fixed as the amplitude of our seasonal forcing function in our transmission model. Further details are described in the Supplementary Methods.

### Phylogenetic analysis

We reproduced previous phylogenetic analysis by Bayesian MCMC inference in BEAST (v1.10.4) to estimate the distribution of the tMRCA for the Central Division cluster^10^. Given the weak branch support for all three Central Division sequences, we forced only the sequences 18A, recovered in 2015, and 1568, recovered in 2016, to form a monophyletic taxon set and estimated the tMRCA for these two samples. Further details on the Fiji sequences, primers used and the remainder of the BEAST analysis are provided in the Supplementary Methods. The sequences and .xml file generated with BEAUti for the BEAST analysis are available on GitHub (10.5281/zenodo.4487358)^35^.

### Transmission model

We developed a model which had flexibility to consider six possible factors: prior population immunity, accumulation of herd immunity during the outbreak, seasonal variation in climate (Supplementary Fig. S4), introduction time (Supplementary Fig. S6), interaction between DENV and ZIKV resulting from cross-protection, and inherent viral transmissibility. We excluded antibody-dependent enhancement between DENV and ZIKV from our model as this would result in an increase in reported ZIKV cases and a comparison of our surveillance and serological data showed that very few infections were recorded as cases of ZIKV.

We developed a deterministic susceptible-exposed-infectious-recovered compartmental transmission model with a dynamic human population (Supplementary Fig. S7). The model included seasonal forcing on the transmission rate using a sinusoidal function and a temporary reduction in transmission in March 2014 from a mosquito clean-up campaign using a flexible sigmoid function^8^ (Supplementary Fig. S5). We also allowed a proportion of those infected with DENV-3 during the 2013/14 outbreak to potentially be temporarily protected against ZIKV infection. People left the recovered compartment to reflect waning ZIKV antibodies over time, however they remained immune to ZIKV.

We used a symmetric logistic function to reflect introduction of ZIKV infection into the population in our model, with the midpoint, width and peak of the function to be estimated. This created a flow of individuals to the *I* compartment, which seeded the early outbreak dynamics. Further details are available in the Supplementary Methods.

Deterministic models can generate artificially cyclical epidemics. To better reflect reality, we included two conditions explicitly in the model. Firstly, there had to be at least one infectious individual for the virus to transmit to prohibit virus persistence at implausibly low levels over the low-transmission season. Secondly, we set the number of infectious introductions to zero if the effective reproduction number was below 1. This prevents epidemic take-off at implausible points of the year.

### Model fitting and comparison

We initially fitted the transmission model to DENV-3 surveillance and serological data to estimate parameter value relating to the seasonal forcing and clean-up campaign in March 2014. These parameters were then fixed for the ZIKV transmission model. When modelling ZIKV, we fixed the parameters for the concurrent DENV-3 epidemic such that *R*_0_ = 1.3 and a proportion of those infected with DENV-3 would become temporally immune from ZIKV.

Full details on the model fitting process (Supplementary Fig. S3) and the full parameter set (*θ*) estimated are given in the Supplementary Information (Supplementary Table S3). For the ZIKV transmission model an informative prior was used for the introduction time. We fitted an empirical distribution to the posterior distribution presented in our study (Fig. 1c) and used this as our prior. Since the phylogenetic posterior was not normally distributed and had a wide highest posterior density (HPD), the prior information in the transmission model fitting was weak. Parameter estimates were constrained within realistic bounds for the width and peak of the infected introductions function and the two baseline transmission rates. The transmission model was jointly fitted to case and serological data using adaptive MCMC with a Metropolis-Hastings algorithm. We assumed that cases were distributed according to a negative binomial distribution and the proportion seropositive at each serosurvey was binomially distributed.

To compare different models under various assumptions we fixed certain parameters in our model (Supplementary Notes). The joint posterior distribution of each model was obtained from 20,000 MCMC iterations, each with a burn-in of 8000. We used adaptive MCMC by adjusting the covariance matrix used to re-sample and obtain a target acceptance rate of 0.234^36^. We used the DIC to compare model fits (Supplementary Table S5). Once we identified our best fitting model, we obtained the joint posterior distribution presented in this study from 1,200,000 MCMC iterations on two chains, each with a burn-in of 480,000. The Supplementary Information includes the DENV-3 model fit (Supplementary Fig. S8), trace plots (Supplementary Figs. S13 and S15), density plots (Supplementary Figs. S9 and S10) and a parameter correlation plot (Supplementary Fig. S14) for the main ZIKV model fit, as well as results from the model comparison analysis (Supplementary Figs. S16–S21).

All models were implemented in R version 4.0.2^37,38^ using the mvtnorm^39^ and deSolve packages^40^ and parallelised using the doMC library^41^.

### Reporting summary

Further information on research design is available in the Nature Research Reporting Summary linked to this article.

## Supplementary information

Supplementary Information

Peer Review File

Reporting Summary

## Data Availability

The surveillance, serological and sequence data used in this study have been deposited in GitHub (10.5281/zenodo.4487358)^35^.

## Code availability

Microsoft Excel (version 16.44) and R (version 4.0.2)^37,38^ were used to clean and process data. All models were implemented in R version 4.0.2^37,38^ using the mvtnorm (version 1.1)^39^, truncnorm (version 1.0.8)^42^ and deSolve (version 1.28)^40^ packages and parallelised using the doMC (version 1.3.6)^41^ package. An MCMC sampler was written in R^43^ to fit the mathematical model to multiple data sources using tidyverse (version 1.3.0)^44^. Figures were created with the ggplot2 package (version 3.3.2)^45^. BEAST and BEAUTi (version 1.10.4) were used to analyse sequence data^46^. All code used in this analysis are available at https://github.com/a-henderson91/fiji-zikv-model (https://doi.org/10.5281/zenodo.4487358)^35^.
