# Supplementary material for: Interactions between timing and transmissibility explain diverse flavivirus dynamics in Fiji
Source: Nat Commun. 2021 Mar 15;12:1671. doi: 10.1038/s41467-021-21788-y (PMC7961049; doi:10.1038/s41467-021-21788-y)
Supplement: Supplementary file 1 — Supplementary Information [file 41467_2021_21788_MOESM1_ESM.pdf]

Interactions between timing and transmissibility explain diverse flavivirus dynamics in Fiji

Supplementary Information

Alasdair D. Henderson[1*], Mike Kama[2], Maite Aubry[3], Stephane Hue[1], Anita Teissier[3], Taina Naivalu[4], Vina D. Bechu[2], Jimaima Kailawadoko [2], Isireli Rabukawaqa [2], Aalisha Sahukhan [2], Martin L. Hibberd[1], Eric J. Nilles[5], Sebastian Funk[1], Jimmy Whitworth[1], Conall H. Watson[1,6], Colleen L. Lau[7], W. John Edmunds[1], Van-Mai Cao-Lormeau[3], Adam J. Kucharski[1]

[1] Centre for the Mathematical Modelling of Infectious Diseases, Department of Infectious Disease Epidemiology, London School of Hygiene & Tropical Medicine, London, United Kingdom

[2] Fiji Center for Diseases Control, Suva, Fiji

[3] Institut Louis Malardé, Papeete, Tahiti, Polynésie française

[4] Fiji National University, Suva, Fiji

[5] Harvard Humanitarian Initiative, Cambridge, Massachusetts, USA

[6] Epidemic diseases Research Group Oxford, University of Oxford, United Kingdom

[7] Australian National University, Canberra, Australia

*Corresponding author e-mail: alasdair.henderson1@lshtm.ac.uk

Supplementary Methods

Study setting

There are two major islands in Fiji in terms of population and economic activity. Vanua Levu, in the Northern Division, and Viti Levu which is split between the Central and Western Divisions (Supplementary Figure 2). Approximately 80% of the Fijian population of 884,887 people live in Central and Western Division. The capital city, Suva, is in the southeast corner of Viti Levu and is home to 94,088 Fijians[1].

Age has been shown to be an important risk factor for the severity of DENV clinical presentation since older people are more likely to have experienced DENV infection and even in endemic settings[2,3]. Consideration of the age distribution of a population is therefore important when studying arbovirus transmission. The median age in the Fijian population as recorded in the 2017 census was 27.5 years and 20% of the population are children younger than 10[4] (Supplementary Figure 1).

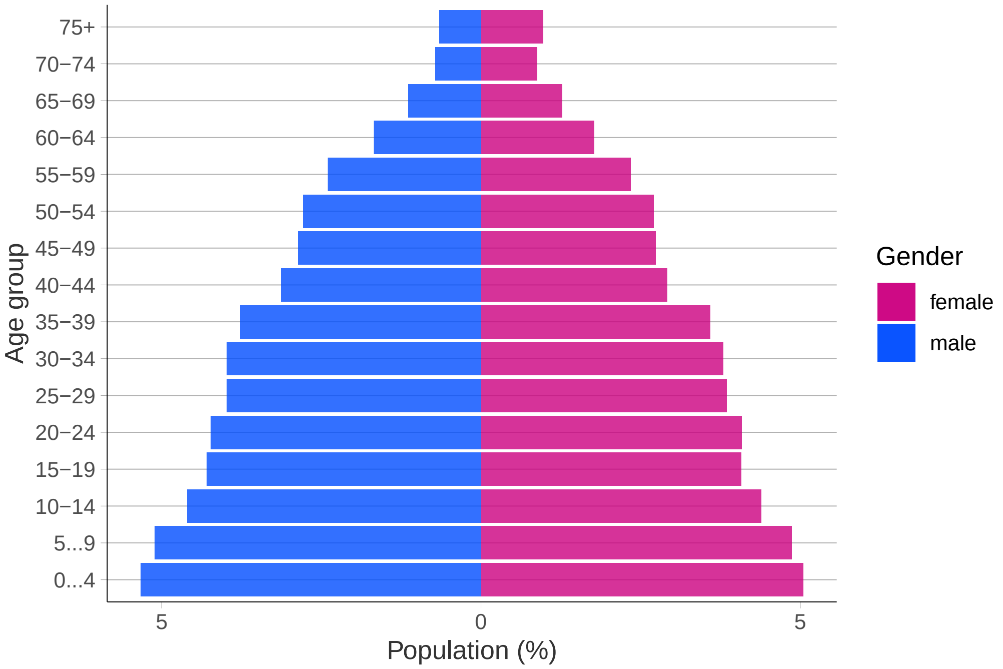

Supplementary Figure 1: *Age pyramid of Fiji from census data collected in 2017*[4]

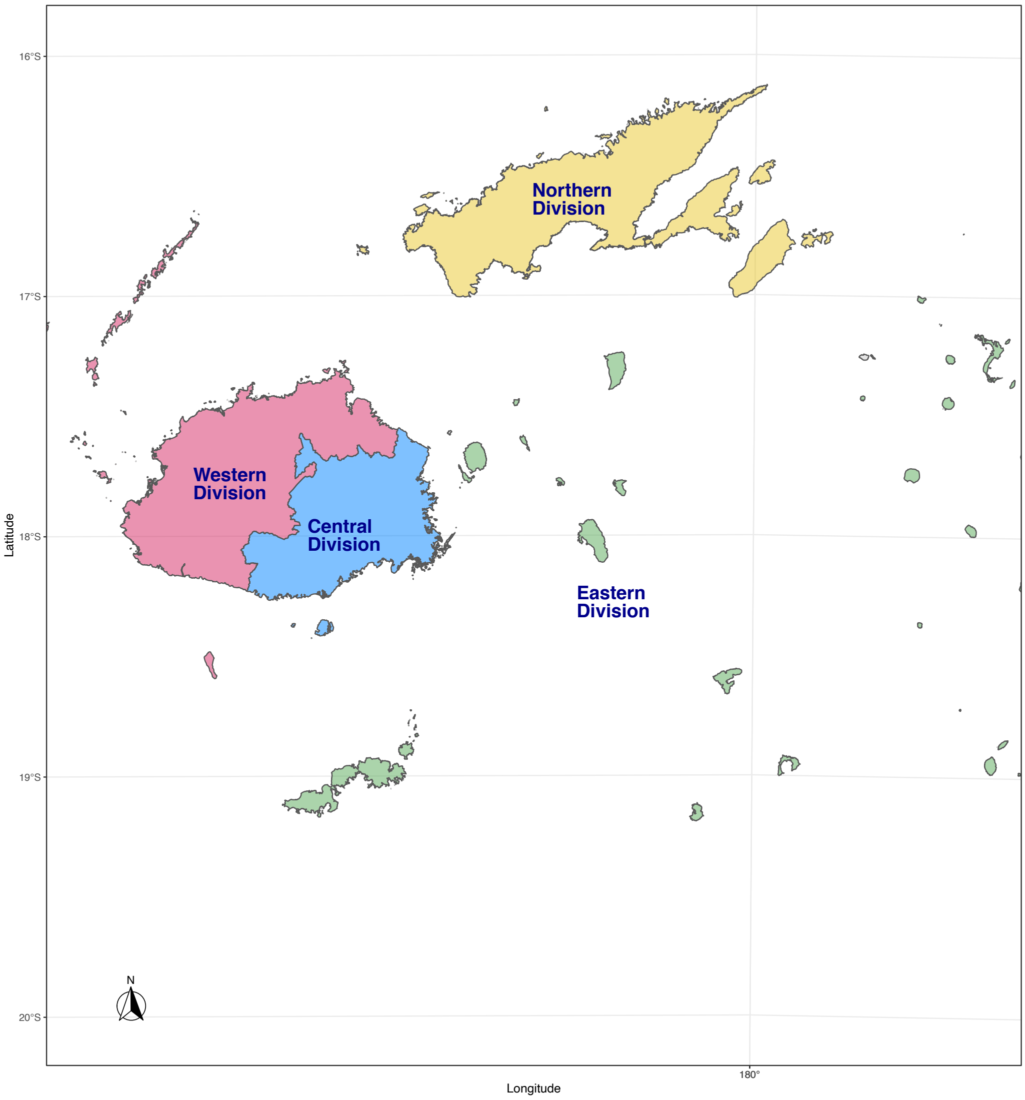

Supplementary Figure 2: *Map of Divisions within Fiji*[5]*. This study was conducted in the Central Division of Fiji (blue).*

Data

Surveillance data

Between June 2015 and August 2017 there were 16 confirmed cases of ZIKV through laboratory surveillance in Central Division, Fiji. Surveillance in Fiji is coordinated by the Ministry of Health, with ZIKV cases were confirmed by four sources. Serum samples from patients with acute fever and rash and/or dengue-like illness (AFR/DLI) were referred to Institut Louis Malardé, French Polynesia, over the period January 2013 to August 2017. Here, reverse transcription polymerase chain reaction (RT-PCR) was used to detect ZIKV. Between March 2016 and August 2017 these tests were performed on site at Mataika House, Suva, Fiji. In addition, saliva samples were submitted to ZIKV RT-PCR. Finally, between March and July 2016, additional samples were referred to the Environmental Science and Research Limited (ESR, New Zealand) and the Environmental Health Institute (EHI, Singapore) for ZIKV molecular testing.

Over the period 27th October 2013 to 31st August 2014, there were 12,413 DENV-3 suspected cases reported in Central Division (i.e. laboratory tested or confirmed or dengue-like illness)[6]. For the lab-confirmed cases, date of testing was used to compile weekly case incidence time series; for the dengue-like illness data, date of presentation to a health centre was used, as these dates were most complete.

Serological data

We conducted a longitudinal seroepidemiological survey over the period 2013-2017 with three visits to the same participants in Central Division, Fiji, in November 2013, November 2015 and June 2017. The original sampling used population-representative sampling to identify nursing zones from Central Division, Fiji, for inclusion. This selected 28 nursing zones from the Central Division. One community was then chosen from each nursing zone and 25 households randomly selected from the community and a participant was recruited at random from each household[7,8]. Each cluster in Central Division had a maximum of 25 participants. In 2017, participants were recontacted through last known addresses, phone numbers and the assistance of local nurses. Once informed consent was obtained, a 5ml blood sample was collected and a short questionnaire conducted. The sample was processed and sent for testing at Institut Louis Malardé in French Polynesia.

Samples collected in 2013, 2015 and 2017 were tested for detection of immunoglobulin class G (IgG) antibodies against ZIKV using a recombinant antigen-based microsphere immunoassay (MIA)[9,10,11]. Recombinant antigens of ZIKV were incubated with serum samples. The cut-off for ZIKV antigen were identified using positive and negative control sera analysed by ROC curve[12]. Sera samples with values above this cut-off were considered positive for the detection of ZIKV specific antibodies, which was interpreted as evidence of previous ZIKV infection[6,10,11,13]. The sensitivity and specificity of the MIA assay were respectively 100% and 100% for DENV-1, 89.5% and 97.1% for DENV-2, 100% and 100% for DENV-3, 96.9% and 100% for DENV-4, and 79.6% and 94.9% for ZIKV[11].

Molecular data

A previous study details the recovery of the envelope (E) gene of ZIKV strains from Fiji and the original phylogenetic analysis that informed this study[13]. The sequences from Central Division were recovered from two saliva samples collected in 2015 and a serum sample collected in 2016. The retrieval of sequences from GenBank has been detailed previously[13]. In brief, sequences were retrieved from GenBank and selected using nucleotide BLAST searches[14]. We retained all sequences with a reported date of sampling and country of origin sharing more than 99% genetic identity to the Fiji sequences. We removed duplicates as done in the original study, but we retained all sequences including those from Europe and Africa. In total, the ZIKV alignments contained 120 sequences including 5 from Fiji, 3 of which were from Central Division.

Climate data

We collated daily maximum and minimum temperature from the Fiji Meteorological Service which covered the study period up to June 2017. We calculated the daily average temperature as the mean of the maximum and minimum temperature recorded on that day.

Modelling

Data on ZIKV transmission in Fiji was sparse and apparently contradictory. Serological data showed evidence that approximately 17% of the population developed ZIKV-specific antibodies between November 2013 and November 2015, however only 2 cases were confirmed in surveil-

lance data over that period. To model the underlying transmission dynamics, we collected data from other sources that could inform ZIKV transmission dynamics in Fiji between 2013 and 2017. A summary of available data and how it was included in our final ZIKV transmission model is shown in Supplementary Figure 3.

Each step in this analysis is outlined in detail later in this section but is briefly summarised here. We initially estimated the dynamics of seasonal forcing on transmission from temperature data in Fiji and used these values in a model of the 2013-14 DENV-3 epidemic. This DENV-3 model was fitted to DENV-3 surveillance and serology using a model similar to a previous study in Fiji[6]. Prior distributions were specified for the full ZIKV transmission model for parameters determining the seasonal forcing of transmission (β_{amp}, β_{mid}) and the effect of a mosquito clean-up campaign in March 2014 on arbovirus transmission (β_{base}) by using *a poseriori* estimates from this DENV-3 model.

At the same time, we performed a phylogenetic analysis of 3 ZIKV sequences from Central Division, Fiji, aligned with 117 other global ZIKV (Asian lineage) sequences. From this analysis we obtained an estimate of the distribution of the tMRCA for the Central Division cluster and used this as a prior for the midpoint of the introduction of ZIKV to Central Division (ψ_{m}).

Finally, these informative priors were used in the full ZIKV transmission model which was fitted to both surveillance and serological data using an Markov Chain Monte Carlo (MCMC) framework.

Modelling seasonal forcing using temperature data

We assumed a linear relationship between temperature in Fiji and the relative transmission of ZIKV based on research from Mordecai et al.[15]. The authors of this study integrated data from several laboratory experiments into a mathematical model of temperature-dependent transmission. The study defined the relationship between temperature and relative R_0 of ZIKV transmitted by *Aedes aegypti* mosquitoes and found maximal transmission occurring in a range from 26-29°C. Data from this study were publicly available and are shown in Supplementary Figure 4 along with the median and interquartile range of the average daily temperature from Fiji over our study period. Although the relationship between temperature and R_0 is non-linear, it is mostly linear in the range of temperatures observed in Fiji across our study period.

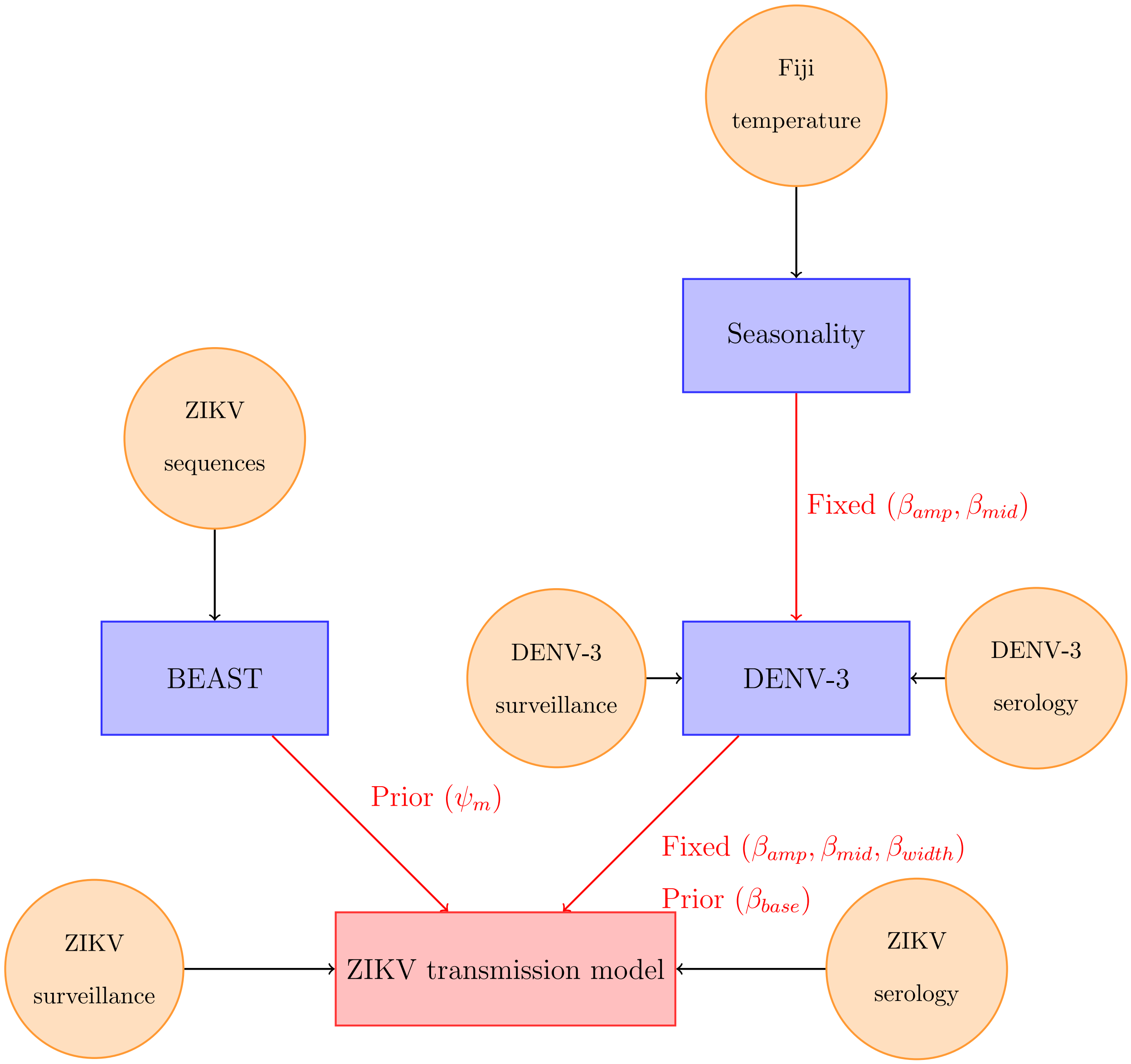

Supplementary Figure 3: *Schematic of modelling approach and available data. The final ZIKV transmission model (red rectangle) used direct data inputs on ZIKV surveillance and serology. Data (orange circles) were used to fit models (blue rectangles) and estimate certain parameters. Information from these model fitting processes was incorporated into this final transmission model through specification of informative priors and fixed values (red arrows)*

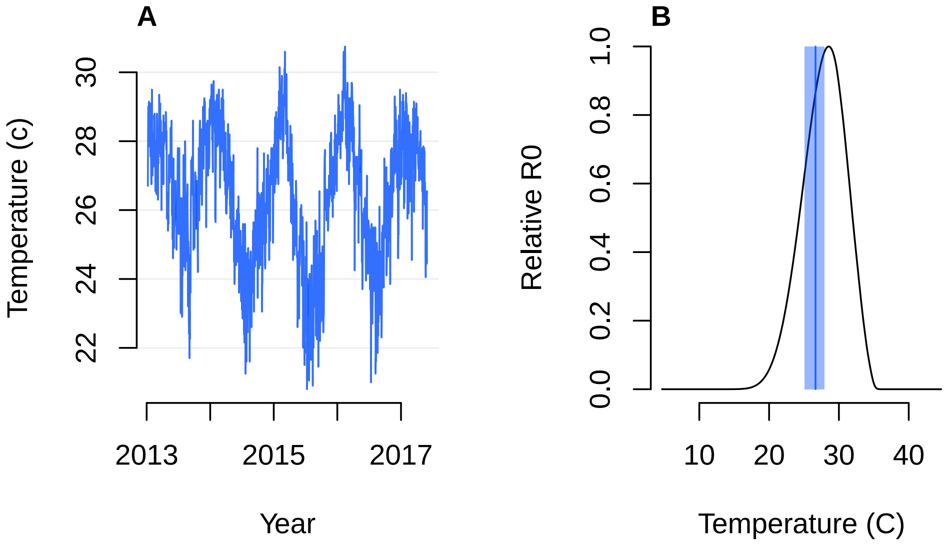

Supplementary Figure 4: *A, daily temperature (°C) in Fiji. B, relationship between average daily temperature and R_0 in Suva, Central Division, Fiji. Black line, mean posterior estimate of the relationship between temperature and relative R_0 for arboviruses transmitted by Aedes aegypti mosquitoes*[15]*. Blue line and region, median and interquartile range of average daily temperature in Fiji between November 2013 and June 2017*

Seasonal variation in rainfall and temperature is known to affect the population and ability of the primary vector of ZIKV, *Aedes aegypti* mosquitoes, to transmit viruses[15,16,17]. Annual temperature in Fiji follows a wave-like pattern so to capture variation in transmission over time we defined a sine function with parameters that determine the amplitude (β_{amp}) and midpoint (β_{mid}) of the sine wave (Equation Equation (1)). We assumed that transmission of both DENV and ZIKV would vary seasonally. Under the assumption of a linear relationship, the rate of transmission of ZIKV (β_Z) varied with time t. The transmission rate at time t was defined by the seasonality function:

$$seasonal(t) = 1 + \beta_{\text{amp}} \sin\left(2\pi\left(t + \beta_{\text{mid}}\right)\right) \tag{1}$$

The sine function Equation (1) was fitted to daily average temperature data[18] using MCMC via a Metropolis-Hastings algorithm with weakly informative priors. We assumed that the temperature data at time t was normally distributed with mean μ and standard deviation σ derived from the overall time series temperature data. Let the parameter set $\zeta = (\beta_{\text{amp}}, \beta_{\text{mid}})$, the corresponding estimated temperature data from the sin function $S = \{s_t\}_{t=1}^T$ and recorded temperature data $Y = \{y_t\}_{t=1}^T$. The overall log-likelihood was then:

$$L(\zeta|Y) = \sum_t \log P(y_t|s_t) \tag{2}$$

We used bootstrap samples of the *a posteriori* estimates of β_{amp} and β_{mid} to obtain samples of the sine wave defined in Equation Equation (1). We defined the amplitude of seasonal forcing as the range between peak and low temperature from the sine function rather than the maximum and minimum temperature in the raw data which could have been influenced by outliers. We then used the previously defined relationship between temperature and relative R_0[15] to convert this temperature range into a range of relative transmission of ZIKV or DENV. This value for β_{amp} and the median estimate for β_{mid} was then fixed when modelling DENV-3 and ZIKV transmission subsequently. This reduced the number of parameters to estimate in the main ZIKV transmission model while capturing the effect of temperature fluctuations on arbovirus transmission in Fiji.

Modelling the 2013-14 DENV-3 epidemic and the effect of vector control interventions in March 2014

There is evidence that a vector control campaign reduced transmission during the 2013-14 DENV-3 outbreak[6]. Given the overlapping geographic region of this DENV-3 epidemic and the ZIKV transmission of this study, we assumed that the effect of the clean-up campaign in March 2014 would have the same effect on ZIKV transmission if ZIKV was circulating at this time.

We adapted the control function used by[6] which was a flexible sigmoid function. An example of the relative effect of this function on transmission is shown in Supplementary Figure 5 with parameter values set at initial conditions for the full ZIKV model.

This function is defined by four parameters. According to Equation Equation (3), relative transmission at time t would reduced to level β_{base} with midpoint β_{centre}. In the original analysis relative transmission remained at β_{base} permanently. In this analysis of ZIKV the study period is much longer than that of the DENV-3 outbreak so we adapted the function with a quadratic denominator and a scaling factor of 4. This function then temporarily reduces transmission relative to a baseline level before returning to the original baseline level (Supplementary Figure 5).

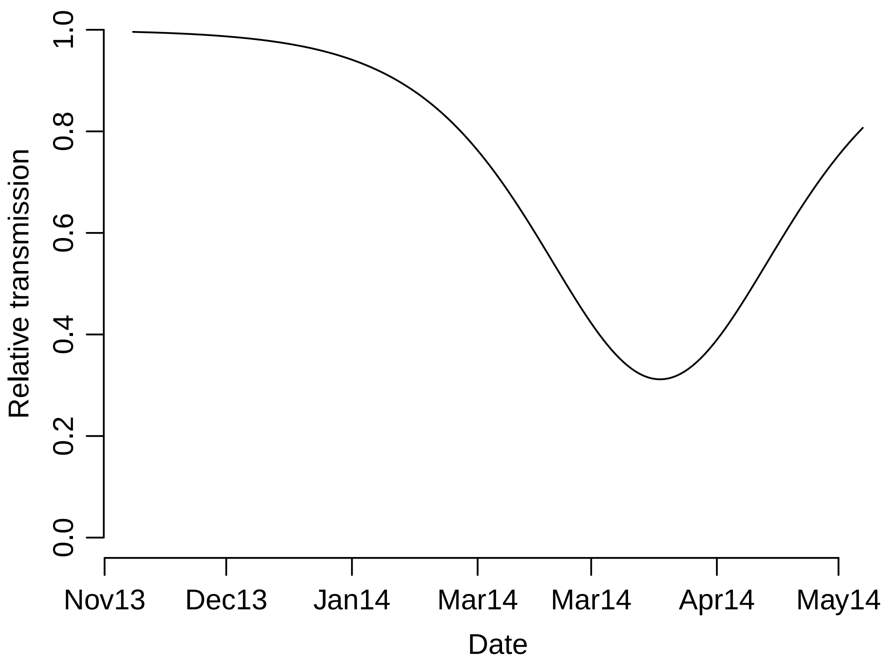

Supplementary Figure 5: *Schematic of control function in ZIKV transmission model*

$$control(t) = 1 - 4\beta_{base}\frac{e^{(\beta_{centre}\, -\, t)/\beta_{width}}}{(1 + e^{(\beta_{centre}\, -\, t)/\beta_{width}})^2} \tag{3}$$

We initially used this function when fitting the 2013-14 DENV-3 epidemic. For this analysis we fixed β_{centre} so that the reduction centred around March 2014 when the vector control campaign was implemented[6]. From fitting to DENV-3 surveillance and serological data we obtained *a posteriori* estimates for β_{base} and β_{width}.

We fitted our SEIR model to surveillance and serological data using MCMC and a negative binomial likelihood over 25,000 iterations using the priors outlined in Supplementary Table 3 below. We fixed the value of β_{width} and used an informative prior for the parameter β_{base} to reduce the number of parameters estimated in the final model.

Phylogenetic modelling of ZIKV sequences

We reproduced previous phylogenetic analysis by Bayesian MCMC inference[13]. We reconstructed phylogenies in nucleotide substitutions per sites and in unit of time ('dated' phylogenies) by Bayesian MCMC inference, using the package BEAST (v1.10.4)[19]. We had a data set of 120 aligned ZIKV sequences and generated a taxon set for two of the Central Division sequences. There were three sequences isolated in Central Division, however the phylogenetic

analysis gave weak branch support for a cluster of all three sequences[13]. There was very strong branch support for the relationship between two of the sequences – 18A, recovered in 2015, and 1568, recovered in 2016 – so we formed a monophyletic taxon set and estimated the tMRCA for these two samples as the estimate for the tMRCA for Central Division as a whole. Details of the sequences from Fiji and the primers used is provided (Supplementary Tables 1 and 2). The analysis was performed using the General Time Reversible (GTR) model to allow flexibility in the nucleotide substitution rate matrix. We used gamma-distributed rate heterogeneity with 4 gamma categories[13,20,21]. We used a coalescent Bayesian skyline tree prior with 10 groups and a piecewise-constant skyline model[22]. The analysis was run using a strict clock and an uncorrelated relaxed clock with a lognormal distribution[23]. The joint distributions were compared and showed improved performance from the uncorrelated relaxed clock model so this model was used. A mean substitution rate prior of 4e−4 substitutions per site per year was used. The MCMC chains were run with 20 million iterations. Convergence of the estimates was considered satisfactory when the effective sample size (ESS) calculated in Tracer v1.6.0 was >200.

Supplementary Table 1: *Characteristics of Zika virus strains sequenced in this study*[13]

Virus	Strain ID	Geographic origin	Collection date	Sample origin	GenBank accession number
Zika	Fiji2015–110715–17A	Suva (Central division)	11 July 2015	Saliva	MG216928
Zika	Fiji2015–110715–18A	Suva (Central division)	11 July 2015	Saliva	MG216929
Zika	Fiji2016–260516–7284	Lautoka (Western division)	26 May 2016	Serum	MG216930
Zika	Fiji2016–220716–1568	Suva (Central division)	22 July 2016	Serum	MG216931
Zika	Fiji2016–030816–1580	Lautoka (Western division)	03 August 2016	Serum	MG216932

Modelling ZIKV: Introduction function

The main ZIKV transmission model used a continuous flow of infectious individuals into the infectious compartment to better represent real introduction dynamics rather than a single introduction event at a single fixed point in time. This does not capture multiple separate introduction waves across multiple years. The number of introductions varied with three pa-

Supplementary Table 2: *Primers used for sequencing of the envelope gene of Zika virus*[13]

Virus	Primer	Genome position	Sequence (5'- 3') [Ref.]
Zika	ZIKVF2	782–801	CGCAAACCTGGTTGGAATCA[24]
Zika	ZIKV	835 882–904	TTGGTCATGATACTGCTGATTGC[25]
Zika	ZIKV	911c 958–937	CCTTCCACAAAGTCCCTATTGC[25]
Zika	ZIKV	1086 1133–1149	CCGCTGCCCAACACAAG[25]
Zika	ZIKV	1162c 1209–1186	CCACTAACGTTCTTTTGCAGACAT[25]
Zika	ZIKVF3	1510–1530	GGAAGCCTAGGACTTGATTGT[24]
Zika	ZIKVR2	1729–1709	CCACGACAGTTTGCCTTTTGG[24]
Zika	ZIKVF4	2172–2193	CAGCACCATTGGAAAAGCATTT[24]
Zika	ZIKVR3	2487–2466	CGAGCACCCCACATCAGCAGAG[24]
Zika	ZIKVR4	2952–2928	GAACCCATGATCCTCCACAAGAAAG[24]

rameters, the peak (ψ_{b}), midpoint (ψ_{m}) and width (ψ_{w}) according to the introduction function (for time t):

$$\psi(t) = (4\psi_{\mathrm{b}}) \left(\frac{e^{(\psi_{\mathrm{m}} - t)/\psi_{\mathrm{w}}}}{(1 + e^{(\psi_{\mathrm{m}} - t)/\psi_{\mathrm{w}}})^2} \right) \tag{4}$$

Where $\psi(t)$ is the number of infectious introductions in time t. Equation Equation (4) produces a symmetric function centred around ψ_m as demonstrated in Supplementary Figure 6. the integral of Equation Equation (4) between $-\infty$ and ∞ gives the total number of ZIKV introductions and is equal to $4\psi_b\psi_w$. ψ_{m} had an informative prior derived from the tMRCA from a phylogenetic analysis. We took the posterior distribution of the tMRCA from the BEAST analysis and used the `fitdistr` function from the R package `MASS` to estimate the empirical mean and standard deviation of this distribution. These parameters were then used to define a Gaussian distributed informative prior for ψ_{m}.

Modelling ZIKV: Transmission model

We developed a model which had flexibility to consider six possible factors that could identify the cause of diverse flavivirus dynamics: prior population immunity, accumulation of herd immunity during the outbreak, seasonal variation in climate, introduction time, interaction between DENV and ZIKV resulting from cross-protection, and inherent viral transmissibility.

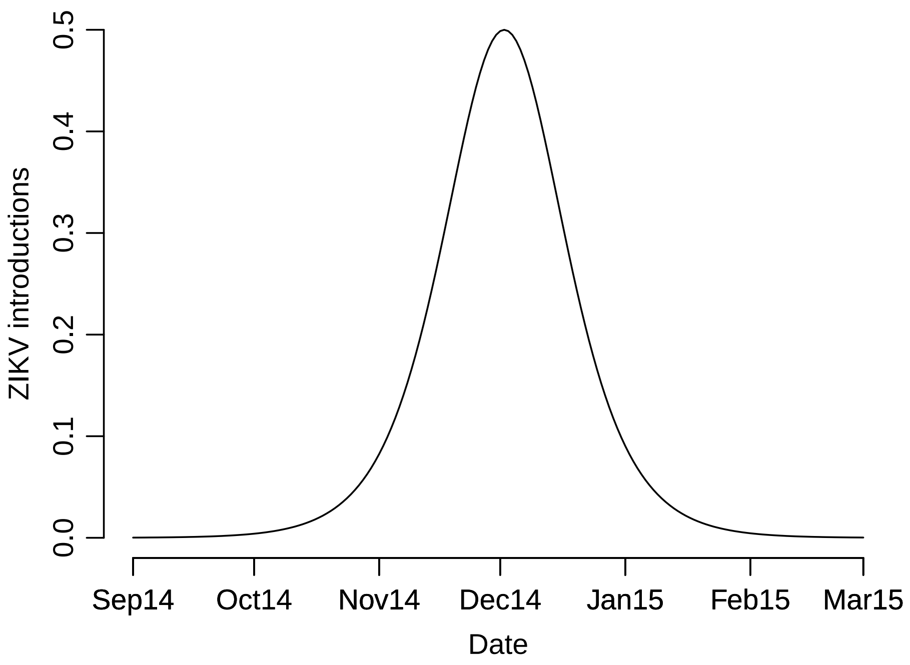

Supplementary Figure 6: *Schematic of introduction function in ZIKV transmission model*

We modelled ZIKV transmission using a deterministic compartmental model with transitions following a susceptible-exposed-infectious-removed (SEIR) structure. The model had ten compartments in total. Upon exposure to ZIKV, humans moved from initially susceptible (S_Z) to a latent class (E_Z), then an infectious class (I_Z) and finally a recovered class (R_Z). During the 2013-14 DENV-3 outbreak, a proportion (χ) of those infected with DENV-3 but susceptible to ZIKV were temporarily removed from the S_Z compartment while clearing the DENV-3 infection. For DENV-3, the human population similarly moved between susceptible (S_D), latent (E_D) and infectious compartments (I_D) before returning to the susceptible-to-ZIKV compartment S_Z over two transitional compartments T_{1D} and T_{2D}. Two compartments were used so that the duration to return to susceptible would follow an Erlang, not exponential, distribution[26,27]. Using an exponential distribution means that there is nonzero density for the null duration in a compartment, so some people can transition artificially rapidly. Using an Erlang distribution solves this problem and is particularly valuable when the average duration of in a compartment is long as it is between T_{2D} and S_Z.

The model included seasonal forcing on the transmission rate using a sinusoidal function and a temporary reduction in transmission in March 2014 from a mosquito clean-up campaign as previously characterised[6] and described in Equations Equation (1) and Equation (3). When this model was used to estimate ZIKV transmission dynamics ("ZIKV transmission model"" in Supplementary Figure 3), the parameters that determined the force of infection for the simul-

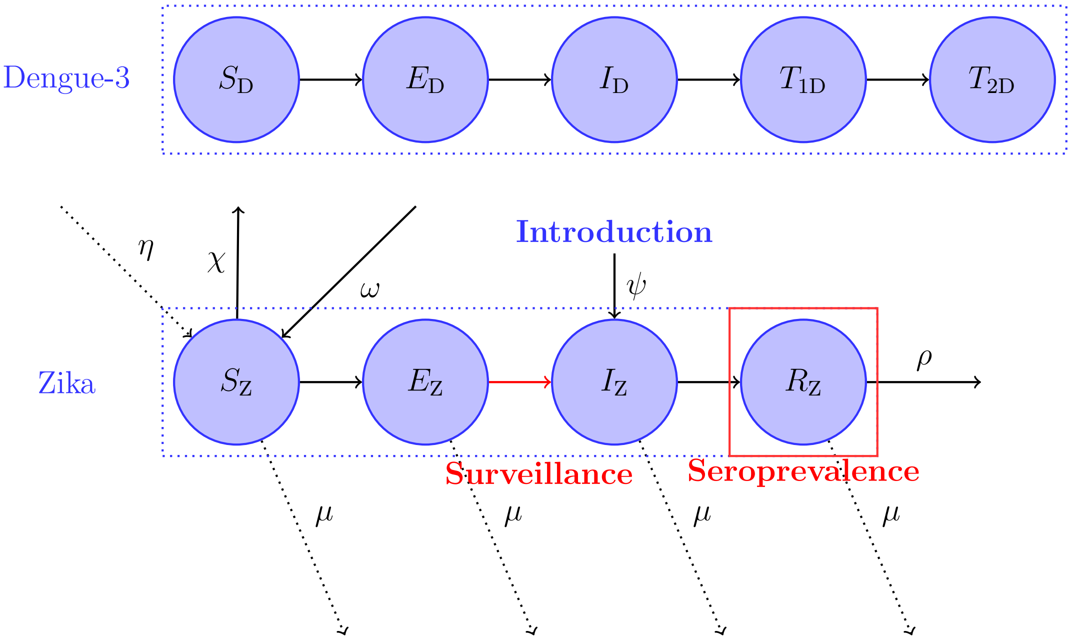

Supplementary Figure 7: *Model schematic of ZIKV transmission model. Dotted lines show demographic transitions. People do not move from the Zika compartments to the Dengue compartments. However, a proportion (χ) of those infected with dengue are temporarily removed from the Zika compartments while they transition from S_D to T_{2D} and return to S_Z at rate ω*

taneous DENV-3 epidemic ($\beta_D, \alpha_D, \gamma_D$) were fixed such that R_0 was 1.3 and a proportion (χ) of those infected with DENV-3 would become temporally immune from ZIKV. For simplicity, we did not include seasonal forcing or mortality in the simultaneous DENV-3 epidemic when the model was run to estimate ZIKV transmission dynamics. When this model was fitted with DENV-3 as the focus ("DENV-3" in Supplementary Figure 3) then seasonality and mortality were included.

The force of infection at time t for ZIKV infection in humans $\lambda_Z(t)$ was as follows:

$$\lambda_Z(t) = \beta_Z \left(seasonal(t) \times control(t) \right) \tag{5}$$

The full model was as follows. The compartment C is introduced to capture the cumulative incidence of infections in the model.

$$^{dS_Z}/_{dt} = \eta - S_Z(\lambda_Z(t)\frac{I_Z}{N}) - \chi\left(S_D\beta_D\frac{I_D}{N}\right) + \chi(2\omega T_{2D}) - \mu S_Z \tag{6}$$

$$^{dE_Z}/_{dt} = S_Z(\lambda_Z(t)^{I_Z}/_N) - (\mu + \alpha_Z)E_Z \tag{7}$$

$$^{dI_Z}/_{dt} = \alpha_Z E_Z - (\mu + \gamma_Z) I_Z + \psi(t) \tag{8}$$

$$^{dR_Z}/_{dt} = \gamma_Z I_Z - (\mu + \rho) R_Z \tag{9}$$

$$^{dC}/_{dt} = \alpha_Z E_Z \tag{10}$$

$$\tag{11}$$

$$^{dS_D}/_{dt} = -\left(S_D \beta_D \frac{I_D}{N} \right) \tag{12}$$

$$^{dE_D}/_{dt} = \left(S_D \beta_D \frac{I_D}{N} \right) - \alpha_D E_D \tag{13}$$

$$^{dI_D}/_{dt} = \alpha_D E_D - \gamma_D I_D \tag{14}$$

$$^{dT_{1D}}/_{dt} = \gamma_D I_D - 2\omega T_{1D} \tag{15}$$

$$^{dT_{2D}}/_{dt} = 2\omega T_{1D} - 2\omega T_{2D} \tag{16}$$

All parameters except the force of infection $\lambda_Z(t)$ and introduction of ZIKV infections $\psi(t)$ were fixed over time. We fixed values for the duration (in days) of the intrinsic incubation period $^1/_{\alpha_Z} = {^1/_{\alpha_D}} = 6.1$ days[28]. Likewise, the duration of the infectious period for ZIKV and DENV-3 in humans $^1/_{\gamma_D} = {^1/_{\gamma_Z}} = 5$ days[29]. We also fixed the duration of cross-protection from DENV-3 $^1/_\omega = 30$ days which was a conservative estimate as ZIKV neutralising antibodies in DENV infected patients had been shown *in vitro* to not persist beyond 6 months previously[30]. In a sensitivity analysis we fitted the model assuming that cross-protection persisted for 6 months. We also fixed the duration of detectable ZIKV antibodies $\rho^{-1} = 400$ days based on our serological studies in French Polynesia and Fiji which found a decrease in ZIKV-specific antibodies 18 months after outbreaks in both locations[11]. This study also showed no evidence that DENV-3 antibodies did not wane over time so the parameter ρ was set to ∞ when running the model for DENV-3.

We set the initial population size N to be 342,000 as per Fiji census data from 2007[4,6]. We used a death rate (μ) of the inverse of life expectancy in Fiji of 67 years[31]. Data from the World Bank shows that the birth rate in Fiji is approximately 2.5 times higher than the death rate[32,33]. We therefore set $\eta = 2.5\mu$.

As detailed above, the force of infection $\lambda_Z(t)$ was time dependent according to seasonal forcing and the effect of a clean-up campaign in March 2014. The force of infection was relative to the baseline transmission rate β_Z, which is used as a single term to incorporate number of female mosquitoes, biting rate, probability of contact and probability of transmission. Without better entomological data from Fiji we used this simpler approach with a single transmission rate.

The introductions of infected individuals $\psi(t)$ was time dependent as defined above in Equation Equation (4). The introduction of DENV-3 to the model was fixed such that 160 individuals were introduced at the start of the outbreak in November 2013, consistent with previous research[6].

The effective reproduction number, R, was defined as follows. The basic reproduction, R_0, was calculated by the same method, but assuming that both humans and vectors were fully susceptible[34]. We estimated the full cycle basic reproduction number (R_0), the average number of new infections in humans from an initial infected human.

$$R = \frac{S_Z \beta_Z \alpha_Z}{(\mu + \gamma_Z)(\mu + \alpha_Z)} \tag{17}$$

Full transmission model fitting

This model was used to separately fitted both the DENV-3 and ZIKV epidemics to serological and surveillance data. We have described equations, compartments and parameters as ZIKV or DENV because ZIKV is the primary focus of the study. However, the same model was used to fit DENV-3 as the primary infection of interest, which was done to obtain estimates for parameters controlling the seasonal forcing and reduced transmission during the clean-up campaign. The parameter χ was set to zero for this DENV-3 model run so that the $S_D, ..., T_{1D}$ compartments did not affect the dynamics in the primary infection compartments.

An informative prior was used for the introduction time for all ZIKV model runs. We fitted an empirical distribution to the posterior distribution presented in our study (**??**C) and used this as the prior for ψ_m. Since the posterior for tMRCA from the phylogenetic analysis had an imprecise estimate, the prior information in the transmission model fitting was weak. We fixed the value of ψ_w to 10 days because of mixing problems from a larger parameter set. To keep the flow of infected individuals to the model below a plausible value we used a uniform prior on the parameter ψ_b. This restricted the total number of introductions for each simulation of the model to be >1 and <800 (Supplementary Table 3).

The full ZIKV transmission model was jointly fitted to case and serological data using adaptive MCMC with a Metropolis-Hastings algorithm. We assumed that cases were distributed according to a negative binomial distribution with mean equal to the number of incident infections

Supplementary Table 3: *Parameter set for arbovirus model fitting*

Parameter	DENV-3 model prior	ZIKV model prior
β_Z	$U(0,1)$	$U(0,1)$
Seasonal amplitude (β_{amp})*	$\mu_{temperature}$	$\mu_{temperature}$
Seasonal midpoint (β_{mid})*	$\mu_{temperature}$	$\mu_{temperature}$
Initial immune (R^0)	$N(0.331, 0.2)$	0
Reporting proportion (r)	$U(0,1)$	$U(0,1)$
Test specificity ($1-\epsilon$)	$N(0, 0.15)$ [11]	$N(0.07, 0.15)$ [11]
Test sensitivity (ζ)	$N(1, 0.15)$ [11]	$N(0.8, 0.15)$ [11]
Cross protection (χ)	NA	$U(0,1)$
Waning ZIKV antibodies (ρ^-1 days)	NA	$U(0,\infty)$
Relative reduction during clean-up campaign** (β_{base})	$N(0.57, 0.15)$ [6]	$N(\mu_{DENV3}, \sigma_{DENV3})$
ZIKV introduction date (ψ_m)	28/09/2013 [6]	$N(\mu_{BEAST}, \sigma_{BEAST})$
ZIKV introductions peak (ψ_b)	400 [6]	$U(0.25, 25)$
Population size (N)	$342,000$ [1]	$342,000$ [1]
Incubation period (α^{-1} days)	6.1 [28]	6.1 [28]
Infectious period (γ^{-1} days)	5 [29]	5 [29]
DENV-3 reproduction number $\left(R_0^{DENV-3} = \frac{\beta_D}{\gamma_D + \alpha_D}\right)$	NA	1.3 [6]
Duration of cross-protection (ω^{-1} days)	NA	30
Death rate (μ^{-1} years)	67.25 [31]	67.25 [31]
Birth rate (η^{-1} years)	2.5μ [32,33]	2.5μ [32,33]

* Seasonal parameters are fixed from fitted values to temperature data

** Clean-up campaign in March 2014

reported in the model $c_t = r(C_t - C_{t-1})$, where r is the reporting proportion. The dispersion parameter ϕ adjusted for the inequality between mean and variance in the case data and was fixed to improve the mixing and convergence of other parameters.

We also fitted the model to the proportion seropositive at each time point of the corresponding serosurvey in 2013, 2015 and 2017. We assumed the proportion seropositive at each survey was binomially distributed with size equal to the population size at the time of the corresponding survey and probability equal to:

$$\frac{R_j\zeta + (1 - R_j)\epsilon}{N_j} \tag{18}$$

Where R_j is the number of people in the recovered (R) compartment and N_j is the population size at time j in our model. Therefore R_j/N_j is the total proportion of true infections that could be detected by an assay. ζ is then the estimated sensitivity of the assay, and ϵ is the estimated false positive rate of the assay. We assumed that sensitivity and specificity were fixed over time which is unlikely to hold true in reality. However, we did not attempt to estimate time varying assay sensitivity and specificity because of the limited size of our data available.

Let the random variable $X_j \sim \text{Bin}(N, {}^{R_j\zeta + (1 - R_j)\epsilon}/_{N})$. The overall log-likelihood for the transmission model with surveillance data $Y = \{y_t\}_{t=1}^{T}$ and serological data $Z = \{z_j\}_{j\in\{2013,2015,2017\}}$ is:

$$L\left(\theta|Y\right) = \sum_t \log P\left(y_t|c_t\right) + \sum_{j\in\{2013,2015,2017\}} \log P\left(X_j = z_j\right) \tag{19}$$

The joint posterior distribution of the parameter set θ was obtained from 1,200,000 MCMC iterations with a burn-in of 480,000. We used adaptive MCMC by adjusting the covariance matrix to obtain a target acceptance rate of 0.234[35]. All models were implemented in R version 4.0.2[36,37] using the `mvtnorm`[38] and `deSolve` packages[39] and parallelised using the `doMC` library[40]. All data and code used in the analysis are available on GitHub (DOI:10.5281/zenodo.4487358)[41].

Model comparison

This full transmission model was designed to be flexible enough to test multiple explanations for the ZIKV outbreak dynamics. The model could capture reduced transmission from inherent

differences in transmissibility, seasonal forcing, increased immunity and temporary cross protection during the 2013-14 DENV-3 epidemic. As a sensitivity analysis of these assumptions we ran the model with certain parameters constrained and jointly fitted the transmission model using adaptive MCMC over 50,000 iterations to compare the output.

The metric to compare model performance was the Deviance Information Criterion. For a likelihood $p\left(y|\Theta\right)$, we define the deviance as:

$$D(\Theta) = -2\log p\left(y|\Theta\right) \tag{20}$$

Where $p\left(y|\Theta\right)$ is the likelihood of the data given Θ and C is a constant that will cancel out when comparing two models.

The DIC can be computed as:

$$DIC = D(\bar{\Theta}) + 2p_D \tag{21}$$

Where $\bar{\Theta}$ is the mean of Θ with respect to the posterior distribution, and p_D is the effective number of parameters, which is approximately equal to half of the variance of the deviance with respect to the posterior distribution:

$$p_D = \frac{1}{2}\widehat{\mathrm{Var}}(D(\Theta)) \tag{22}$$

A difference in DIC of >10 was considered as evidence that the model with the lower DIC was better. A difference between 5 and 10 was considered borderline evidence and any difference less than 5 was considered as no evidence that the models performed differently[42].

Supplementary Notes

Posterior parameter estimates

We fitted our transmission model separately to the DENV-3 and ZIKV epidemics to serological and surveillance data. Parameter estimates from the model are shown in Supplementary Table 4.The estimate for the relative effect of "cross-protection" on ZIKV infection during the DENV-3 epidemic shows no evidence of an effect as the 95% credible interval extends from 0.074 (no effect) to 0.98 (total protection). This is unsurprising since most model simulations had ZIKV outbreaks that started after the DENV-3 outbreak had ended, so there would be no signal about cross-protection in these simulations. The effective sample size – the number of effectively independent draws from the posterior distribution – for the estimated eight parameter are above 100 and six have an ESS greater than 200. The full set of parameter estimates are shown in Supplementary Table 4.

DENV-3 model fit

We fitted our transmission model to DENV-3 surveillance and serological data for the 2013-14 epidemic to estimate the parameter set Θ as in Supplementary Table 3. Supplementary Figure 8 shows the estimated transmission dynamics of DENV-3 using a mathematical model fitted to multiple data sources.

Density plots for the eight estimated parameters from the ZIKV model fit are shown in Supplementary Figure 9. Density plots of the six estimated parameters in the DENV-3 model fit are shown in Supplementary Figure 9

Comparison of prior and posterior estimated ZIKV introduction time in the model

We obtained a posterior estimate for the time of introduction of ZIKV to Fiji by using a previous phylogenetic analysis as a prior in our model. The 95% credible interval for the most likely midpoint of ZIKV introduction extended from October 2014 to February 2015 with a median of January 2015. Our joint inference produced a more precise estimate than the

original phylogenetic analysis alone, which had an inferred tMRCA date of November 2013 (95% HPD interval March 2013-July 2015)[13]. A comparison of the two estimates of ZIKV arrival in Central Division are shown in Supplementary Figure 11.

Hypothetical ZIKV reported cases if ZIKV was reported the same as DENV-3

Supplementary Figure 12 shows the expected observed number of ZIKV cases from our modelled outbreak if ZIKV cases were reported at the same rate as DENV-3 cases were during the 2013-14 epidemic. This demonstrates that the underlying outbreak dynamics were similar in magnitude but that the main difference in the size of outbreaks in surveillance data was the discrepancy in reporting proportions.

MCMC diagnostics and convergence

The trace plots for the eight estimated parameters in the main model are shown in Supplementary Figure 13. Estimation of four of the eight parameters achieve an appropriate level of mixing in all three chains used in the MCMC fitting process. There is poor mixing for the parameter measuring cross-protection because if ZIKV transmits after the DENV-3 outbreak then there is no additional information provided by this parameter. The two parameters for the introduction function mix less efficiently, possibly because of their strong correlation (Supplementary Figure 14).

The trace plots for the DENV-3 model fit are shown in Supplementary Figure 15.

Sensitivity analyses

We tested three of the key assumptions we made when modelling ZIKV transmission. Firstly, we relaxed the assumption that seropositivity wanes in the population. Secondly, we extended the duration of the cross-protection for ZIKV following DENV infection. Finally, we forced ZIKV to start spreading in Fiji before the DENV-3 epidemic. A summary of the three models used for this comparison is presented in Supplementary Table 5. All four models were fitted to the same data using MCMC over 20,000 iterations.

Model A - full model

These are the results from the "best fitting model" but from a shorter fitting process over 20,000 iterations. To reiterate, the key assumptions that have been made are that detectable ZIKV-specific antibodies wane over time and that the model could accurately identify the reporting proportion. The estimated DIC from this main model was 76.8.

Model B - no reduction in seropositivity

We wanted to test the assumption that ZIKV-specific antibodies wane over time below a detectable threshold. There is good evidence for this assumption in serological data from Fiji[11]. However this is a novel concept so we wanted to compare it to a model where antibodies do not wane over time. The results from this alternative model show that, if ZIKV seropositivity does not wane, the estimated seroprevalence from the model fitting does not recapture the observed seroprevalence data (Supplementary Figure 16B). As a result the model fit is worse with a DIC of 103.1 compared to 76.8 from the main model.

Model C - longer period of cross-protection following DENV infection

We chose a conservative estimate for the duration of cross-protection between DENV-3 and ZIKV of 30 days. This proved uninformative in our main model findings since we estimated that ZIKV was introduced in late 2014, long after the DENV-3 epidemic. As a sensitivity analysis we set the duration of cross-protection to 6 months[30] to test whether this would make an early introduction and long suppression of ZIKV during the DENV-3 epidemic capture the observed ZIKV data. However the model still converged on a late 2014 introduction date (December 2014; 95% CrI: Oct 2014–Feb 2015).

Model D - constrain introduction of ZIKV to be before the 2013-14 DENV-3 epidemic

With introduction of ZIKV constrained to 2013, we found that a combination of DENV-3 cross-immunity and reduced transmission from a vector control campaign in March 2014 could have suppressed ZIKV transmission in 2014 (Supplementary Figure 17). However the Deviance

Information Criterion (DIC) from this model (129.8) was much higher than the best fitting model (78.4), suggesting very little support for this alternative explanation. The reason for poor model performance is the extended outbreak duration that resulted from this interaction: DENV did not just influence ZIKV in 2014 in this model: by suppressing ZIKV transmission to a large extent, subsequent multi-year outbreaks of ZIKV during 2015-2017 were less plausible in the model, in contrast with the observed reported cases during this period. This model also produces a higher R_0 of 1.24 (95% CrI: 0.9–1.6) so that ZIKV infections can persist through downward pressure on transmission between late 2013 and late 2014 from seasonality, any cross-protection during the DENV-3 epidemic and the clean-up campaign in March 2014.

MCMC trace plots for sensitivity analyses

The trace plot for the shorter version of the main model (model A) is shown in Supplementary Figure 18. The poor mixing from the alternative models; model B Supplementary Figure 19, model C (Supplementary Figure 20) and model D constrained to an introduction in 2013 (Supplementary Figure 21).

Supplementary Tables

Supplementary Table 4: *Parameter estimates from arbovirus transmission model fitted to the DENV-3 2013-14 epidemic, and ZIKV transmission between 2013 and 2017 (with effective sample size (ESS))*

Parameter	DENV-3 estimate (95% CrI)	ZIKV estimate (95% CrI)	ESS (ZIKV)
Median R_0	1.84 (1.27-2.39)	1.18 (0.82-1.54)	-
Median R	1.08 (0.403-1.61)	0.942 (0.518-1.56)	-
β_Z	0.35 (0.33-0.38)	0.24 (0.23-0.25)	6880
Reporting proportion (%)	16 (12-23)	0.011 (0.0061-0.019)	725
Cross protection	NA	0.51 (0.013-0.98)	158
(1 minus) Test specificity (%)	2.3 (0.35-8.2)	6.3 (4.4-8.5)	1900
Test sensitivity (%)	73 (23-99)	79 (52-98)	1920
ZIKV introduction date (mid)	Oct 2014	Jan 2015 (Oct 2014-Feb 2015)	217
ZIKV introductions (n)	400	394.6 (56.51-963.7)	115
Relative reduction during clean-up campaign (Mar 2014)	0.74 (0.71-0.77)	0.68 (0.64-0.73)	22600
Initial proportion immune	0.29 (0.23-0.35)	0	–
DIC	621.6	76.3	–

Model comparisons

Supplementary Table 5: *Sensitivity analysis of key assumptions in the modelling of ZIKV transmission dynamics in Fiji. Estimated deviance information criterion (DIC) and basic reproduction number (R_0) for each model are shown*

Model		DIC	R_0 (95% CrI)
A	Main model	76.8	1.15 (0.8–1.5)
B	No reduction in seropositivity	103.1	1.06 (0.7–1.4)
C	Longer cross-protection duration	75.9	1.14 (0.8–1.5)
D	ZIKV introduction in 2013	129.8	1.24 (0.9–1.6)

Supplementary Figures

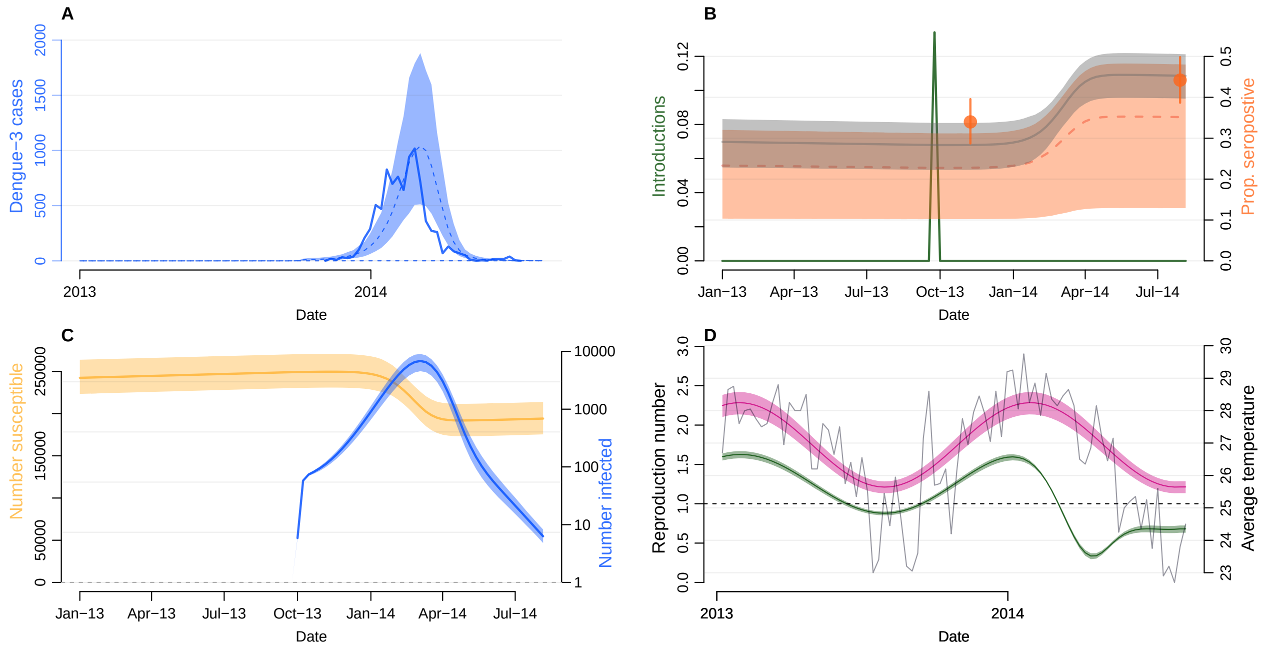

Supplementary Figure 8: *Estimated transmission of DENV-3 in Fiji using a mathematical model and multiple data sources. (A) Blue line, weekly cases of DENV-3. Blue dashed line and region, model estimated median cases of DENV-3 and 95% CrI. (B) Seroprevalence and introduction of DENV-3. Green line, fixed introduction of DENV-3 infected individuals. Grey line and region, estimated proportion of the population that had recovered from ZIKV infection (median and 95% CrI). Orange dashed line and region, estimated median observed seroprevalence and 95% CrI. Seroprevalence includes an estimated 2.3% (0.35–8.2%) false positive rate and 73% (95% CrI: 23–99%) assay sensitivity. Orange dots and vertical lines, estimated ZIKV seroprevalence from 3 serological surveys (mean and 95% binomial CI, n = 458, 327 in 2013 and 2015 respectively). (C) DENV-3 infection dynamics in Central Division. Yellow line and region, median and 95% CrI of the number of people susceptible to DENV-3. Blue line and region, median and 95% CrI of the number infected on the natural log scale. (D) Pink line and region, estimated basic reproduction number for DENV-3 (median and 95% CrI). Green line and region, effective reproduction number (median and 95% CrI). This included an estimated decline in transmission coinciding with a 2014 vector clean-up campaign[6]. Grey line, monthly temperature data from Suva, Central Division*

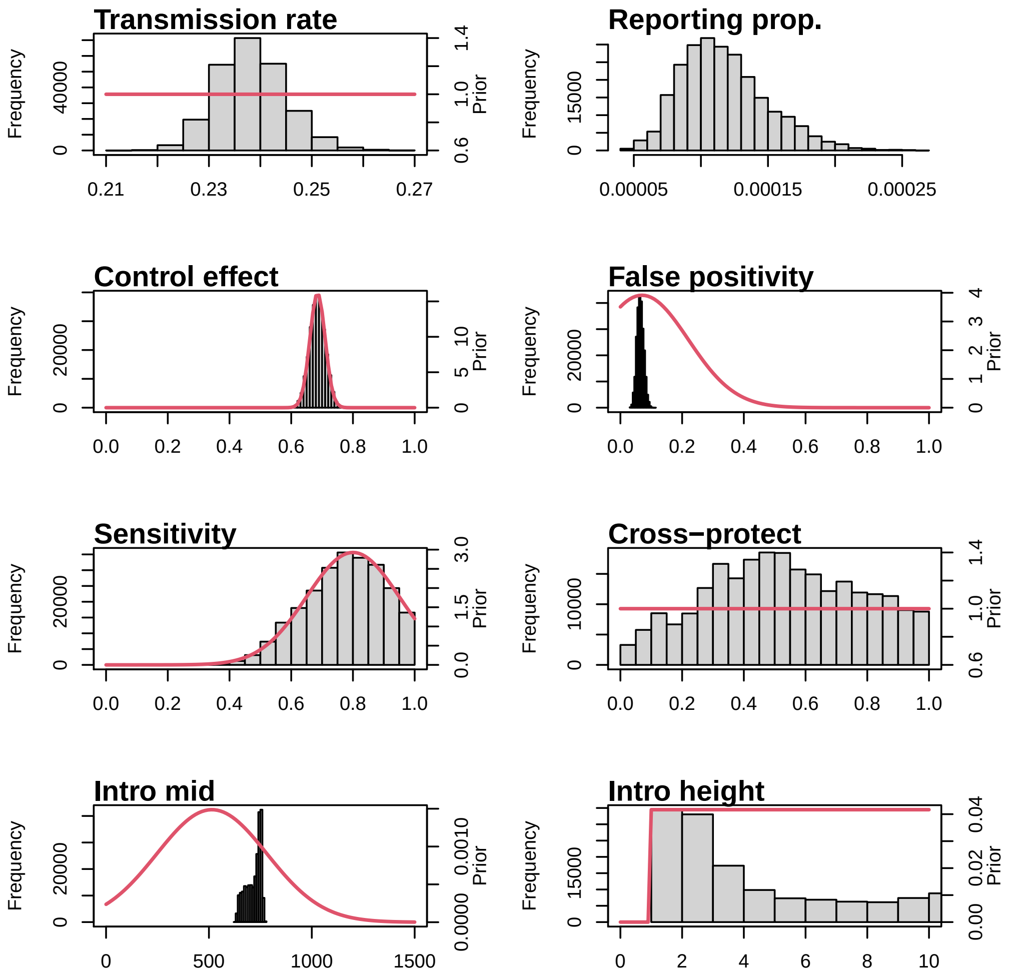

Supplementary Figure 9: *Density plot of estimated parameters in ZIKV transmission model. Grey bars, posterior density. Red lines, prior density. A uniform prior was used for the reporting proportion but is missing from the plot*

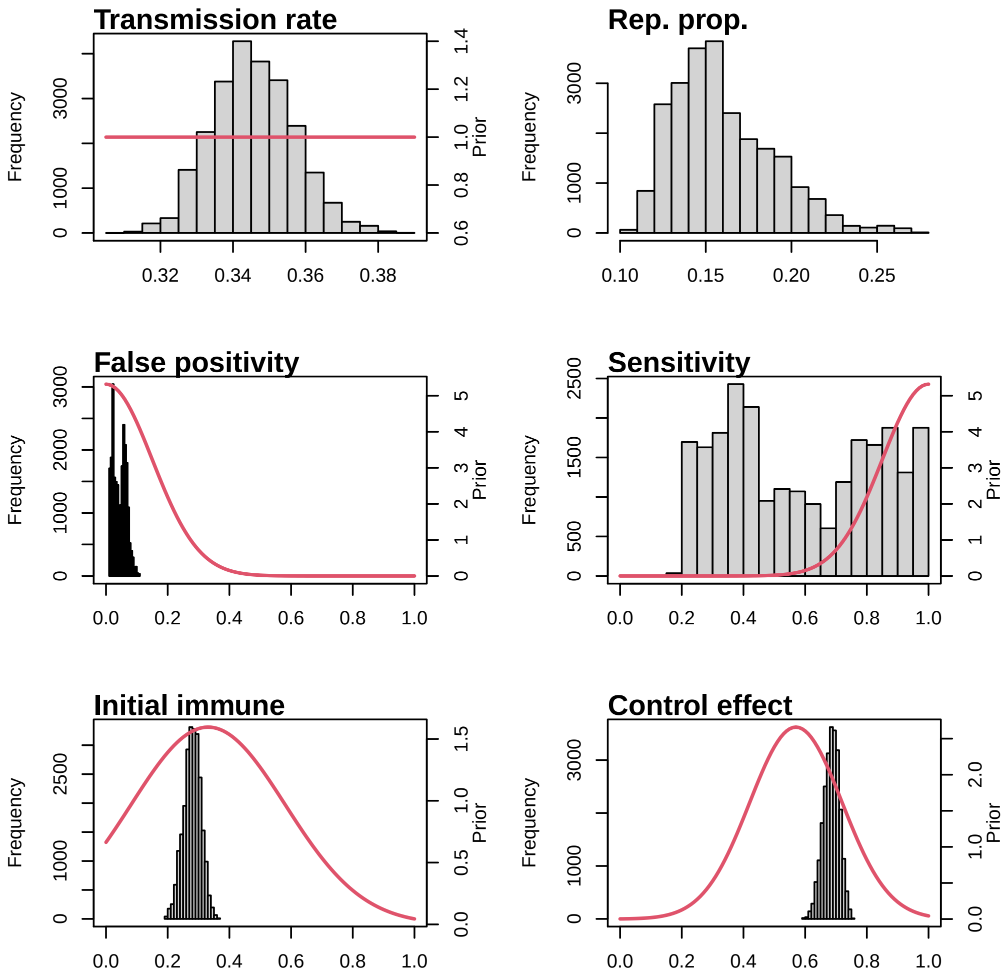

Supplementary Figure 10: *Density plot of estimated parameters in DENV-3 transmission model. Grey bars, posterior density. Red lines, prior density. A uniform prior was used for the reporting proportion but is missing from the plot*

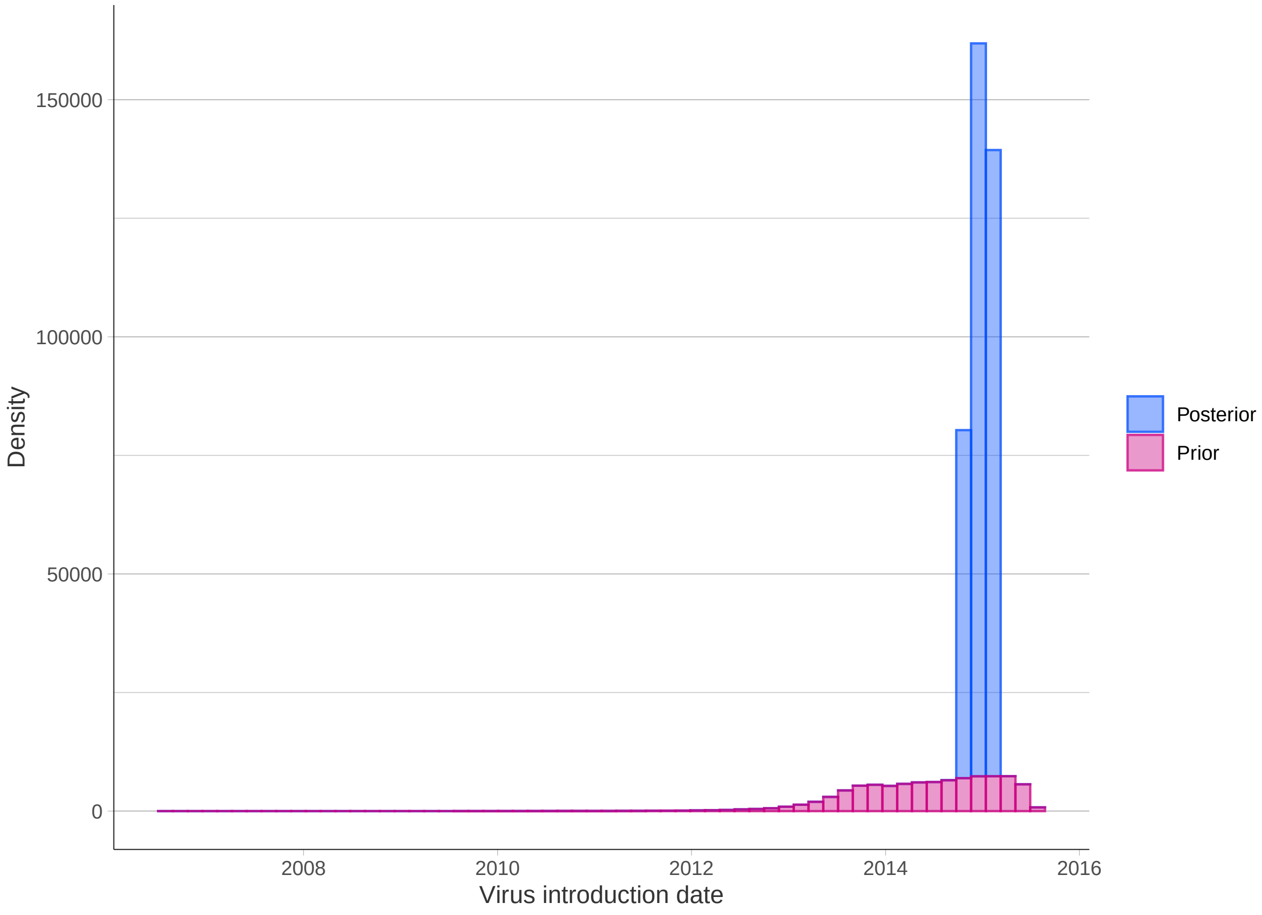

Supplementary Figure 11: *Estimated introduction of ZIKV compared to phylogenetic analysis. Density of estimated introduction time of ZIKV to Central Division, Fiji. Estimate from a phylogenetic analysis of sequence data (pink)*[13] *used as a prior in this analysis. The estimated midpoint* (β_{mid}) *from this transmission model (blue).*

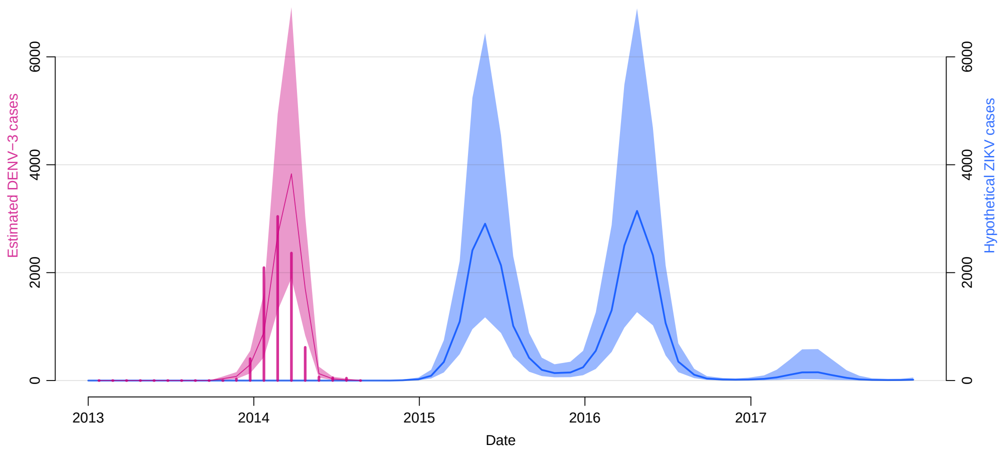

Supplementary Figure 12: *Hypothetical ZIKV case dynamics if the reporting mechanism was equivalent to that of the DENV-3 epidemic. Pink line and region, median estimated DENV-3 cases and 95% CrI. These estimates include an estimated 16% (95% CrI: 12–23%) reporting proportion. Blue line and region, hypothetical reported ZIKV cases if the reporting mechanism for ZIKV was the same as DENV-3 (median and 95% CrI). These estimates use the modelled infections from the ZIKV model but the reporting proportion from the DENV-3 model fit. The time scale for this plot is monthly not weekly, so the observed DENV-3 cases from surveillance data are reproduced on a monthly time scale as vertical lines.*

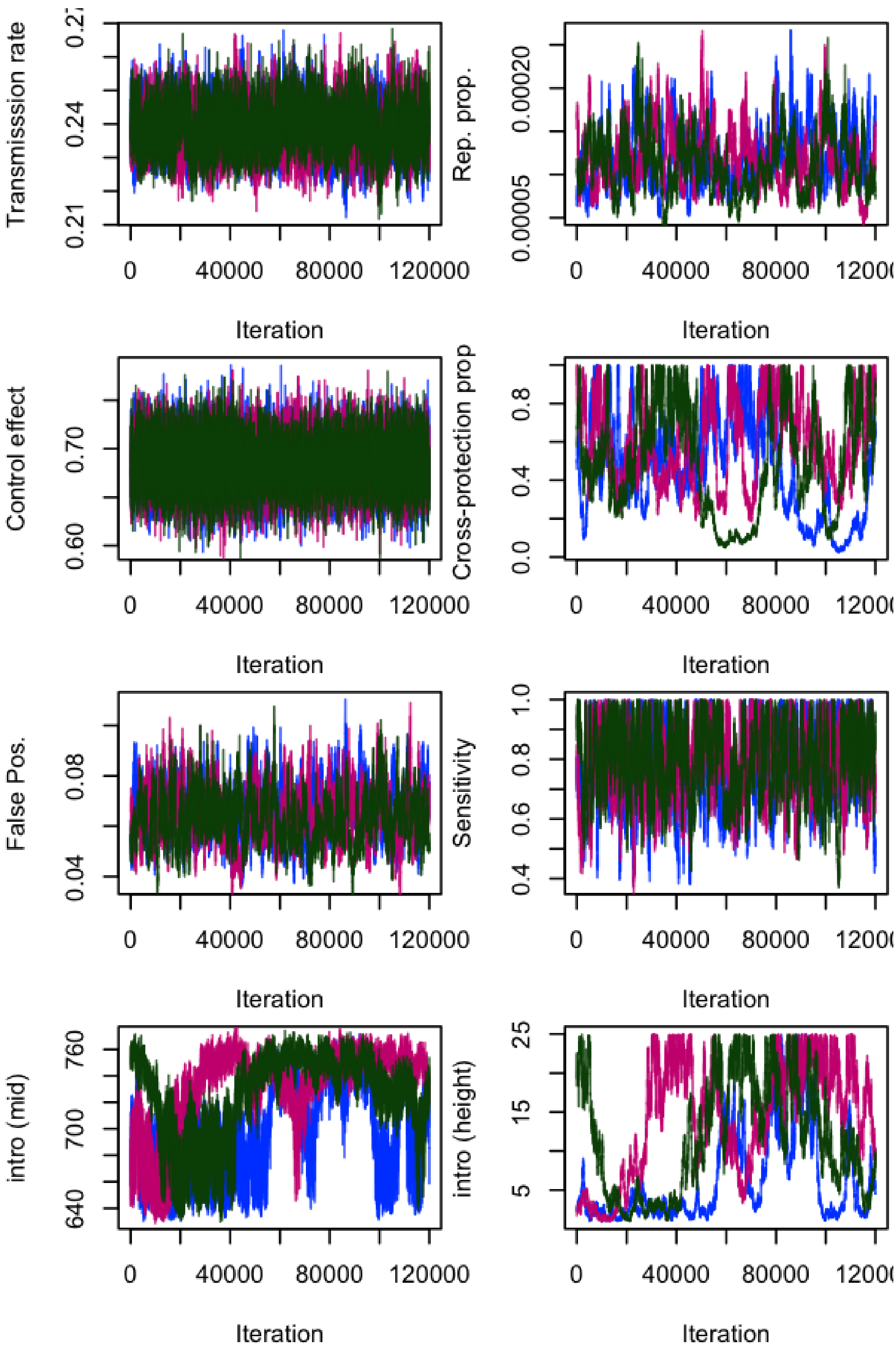

Supplementary Figure 13: *Trace plot of MCMC convergence for full ZIKV transmission model after burn-in of 40%. The three colours represent three separate MCMC chains used in the fitting of the model to surveillance and serological data.*

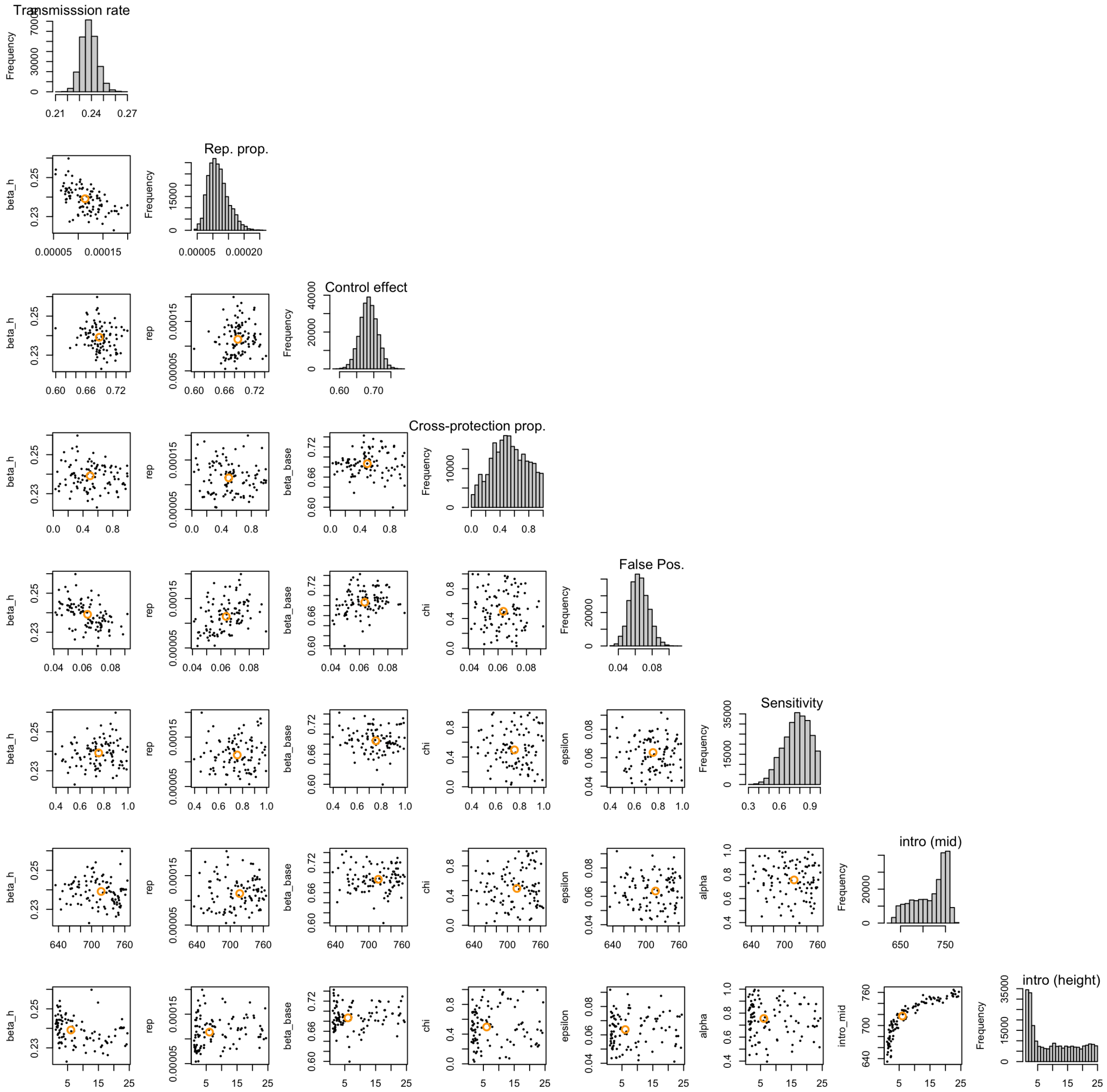

Supplementary Figure 14: *Correlation between estimated ZIKV model parameters. The histograms show estimates of the parameter value. The scatter plot shows the relationship between estimated values of these parameters.*

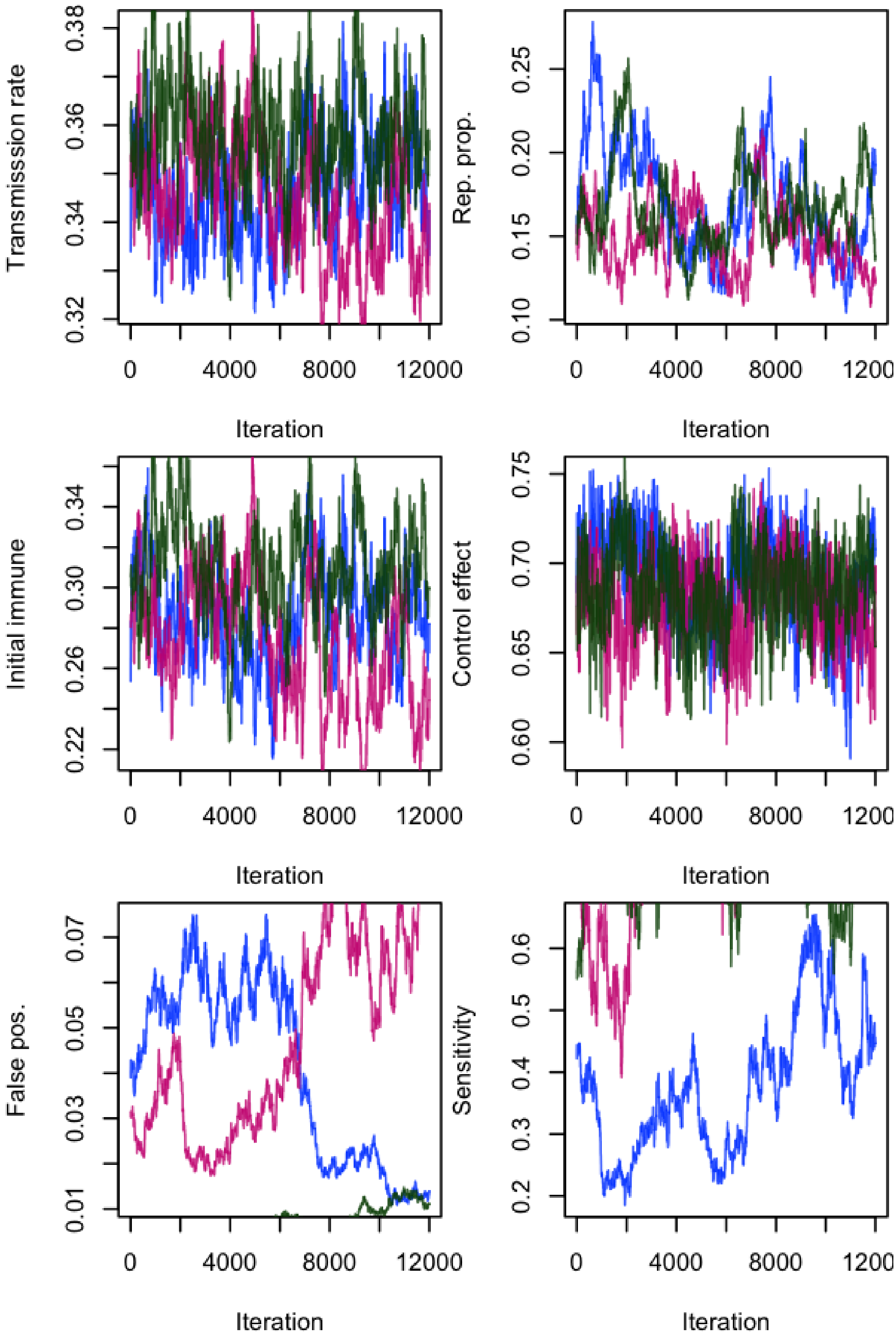

Supplementary Figure 15: *Trace plot of MCMC convergence for full DENV-3 transmission model after a burn-in of 40%. The three colours represent three separate MCMC chains used in the fitting of the model to surveillance and serological data.*

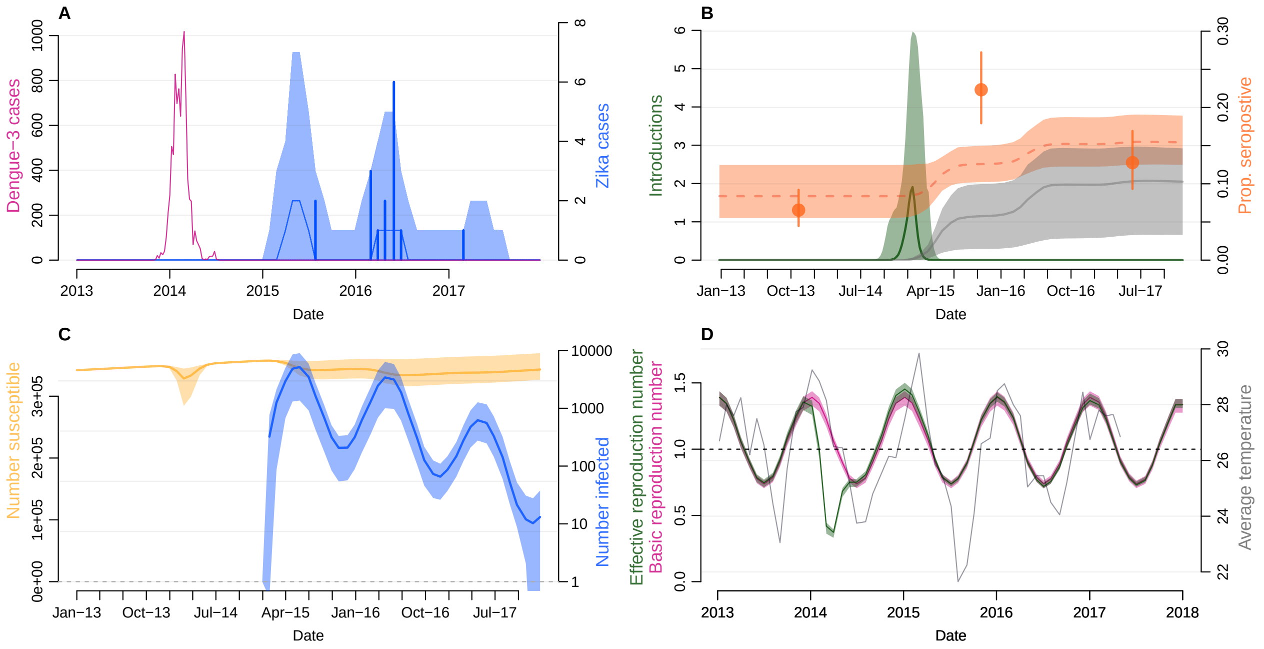

Supplementary Figure 16: *Model B: estimated ZIKV transmission, as in Model A but seropositivity can not decrease. (Estimated DIC: 103.1). (A) Pink line, weekly cases of DENV-3. Blue bars, monthly cases of ZIKV. Blue dashed line and region, model estimated median cases of ZIKV and 95% CrI. (B) Seroprevalence and introduction of ZIKV. Green line and region, estimated median number of introductions of ZIKV infected individuals and 95% CrI. Grey line and region, estimated proportion of the population that had recovered from ZIKV infection (median and 95% CrI). Orange dashed line and region, median estimated observed seroprevalence and 95% CrI. Seroprevalence includes an estimated false positive rate and assay sensitivity. Orange dots and vertical lines, estimated ZIKV seroprevalence from 3 serological surveys (mean and 95% binomial CI, n = 458, 327, 321 in 2013, 2015, 2017 respectively). (C) ZIKV infection dynamics in Central Division. Yellow line and region, median and 95% CrI of the number of people susceptible to ZIKV. Blue line and region, median and 95% CrI of the number infected on the natural log scale. (D) Pink line and region, estimated basic reproduction number for ZIKV (median and 95% CrI). Green line and region, effective reproduction number (median and 95% CrI). This included an estimated decline in transmission coinciding with a 2014 vector clean-up campaign*[6]. *Grey line, monthly temperature data from Suva, Central Division.*

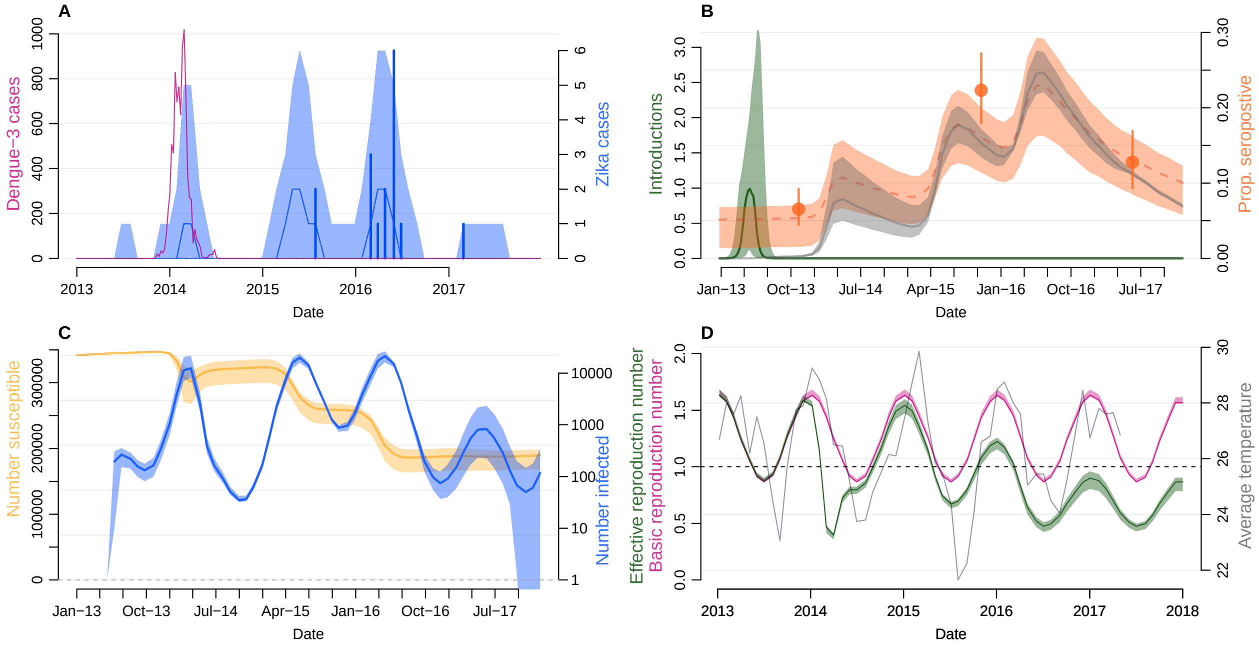

Supplementary Figure 17: *Model D: Estimated transmission of ZIKV in Fiji in a model with ZIKV introduction constrained to 2013 (Estimated DIC: 129.8). (A) Pink line, weekly cases of DENV-3. Blue bars, monthly cases of ZIKV. Blue dashed line and region, model estimated cases of ZIKV and 95% CrI. (B) Seroprevalence and introduction of ZIKV. Green line and region, estimated median number of introductions of ZIKV infected individuals and 95% CrI. Grey line and region, estimated proportion of the population that had recovered from ZIKV infection (median and 95% CrI). Orange dashed line and region, median estimated observed seroprevalence and 95% CrI. Seroprevalence includes an estimated false positive rate and assay sensitivity. Orange dots and vertical lines, estimated ZIKV seroprevalence from 3 serological surveys (mean and 95% binomial CI, n = 458, 327, 321 in 2013, 2015, 2017 respectively). (C) ZIKV infection dynamics in Central Division. Yellow line and region, median and 95% CrI of the number of people susceptible to ZIKV. Blue line and region, median and 95% CrI of the number infected on the natural log scale. (D) Pink line and region, estimated basic reproduction number for ZIKV (median and 95% CrI). Green line and region, estimated effective reproduction number (median and 95% CrI). This included an estimated decline in transmission coinciding with a 2014 vector clean-up campaign[6]. Grey line, monthly temperature data from Suva, Central Division.*

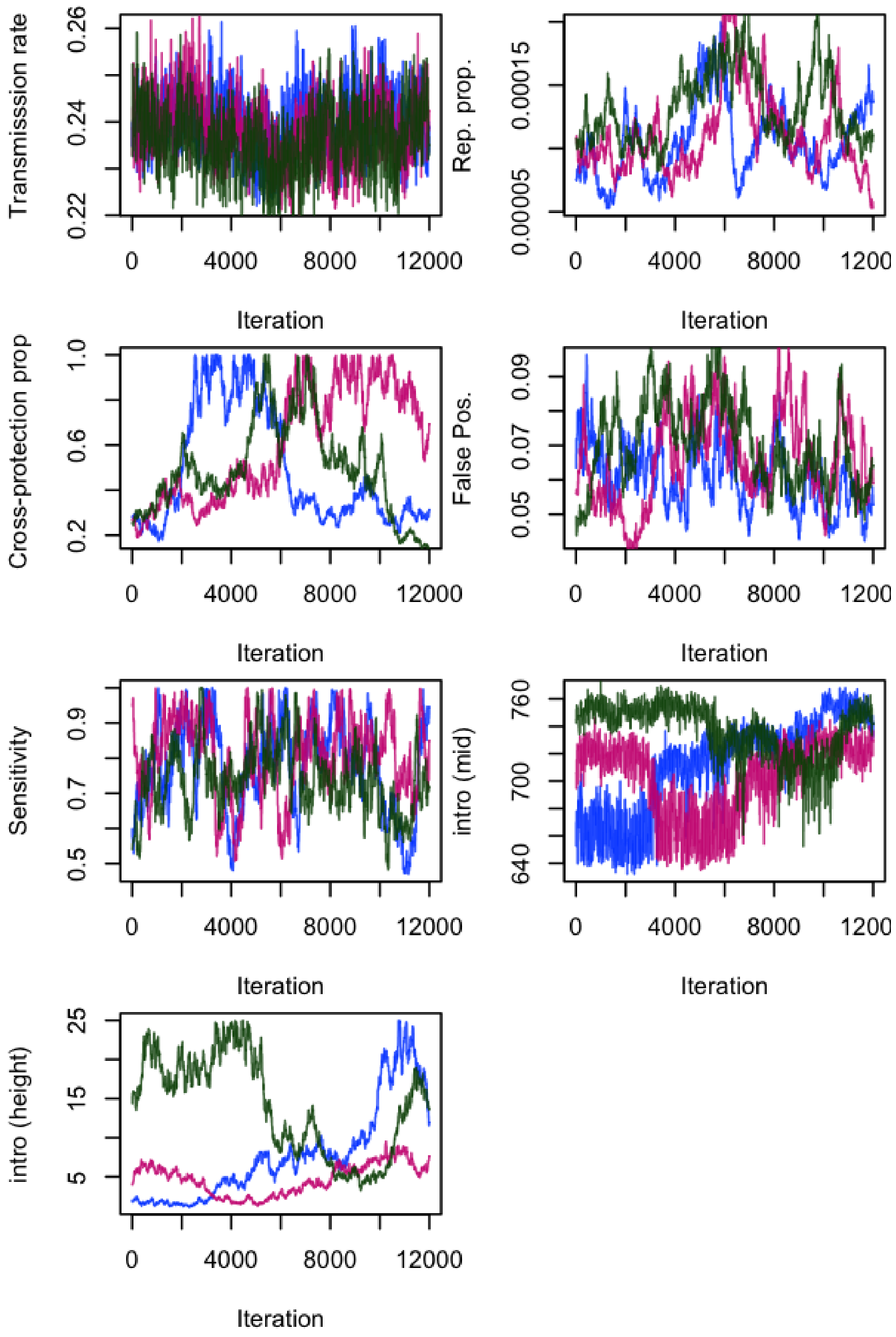

Supplementary Figure 18: *Trace plot of MCMC convergence for Model A after burn-in of 40%. The three colours represent three separate MCMC chains used in the fitting of the model to surveillance and serological data. A condensed plotting area is shown here so sometimes chains may not be visible, this is a sign of poor convergence*

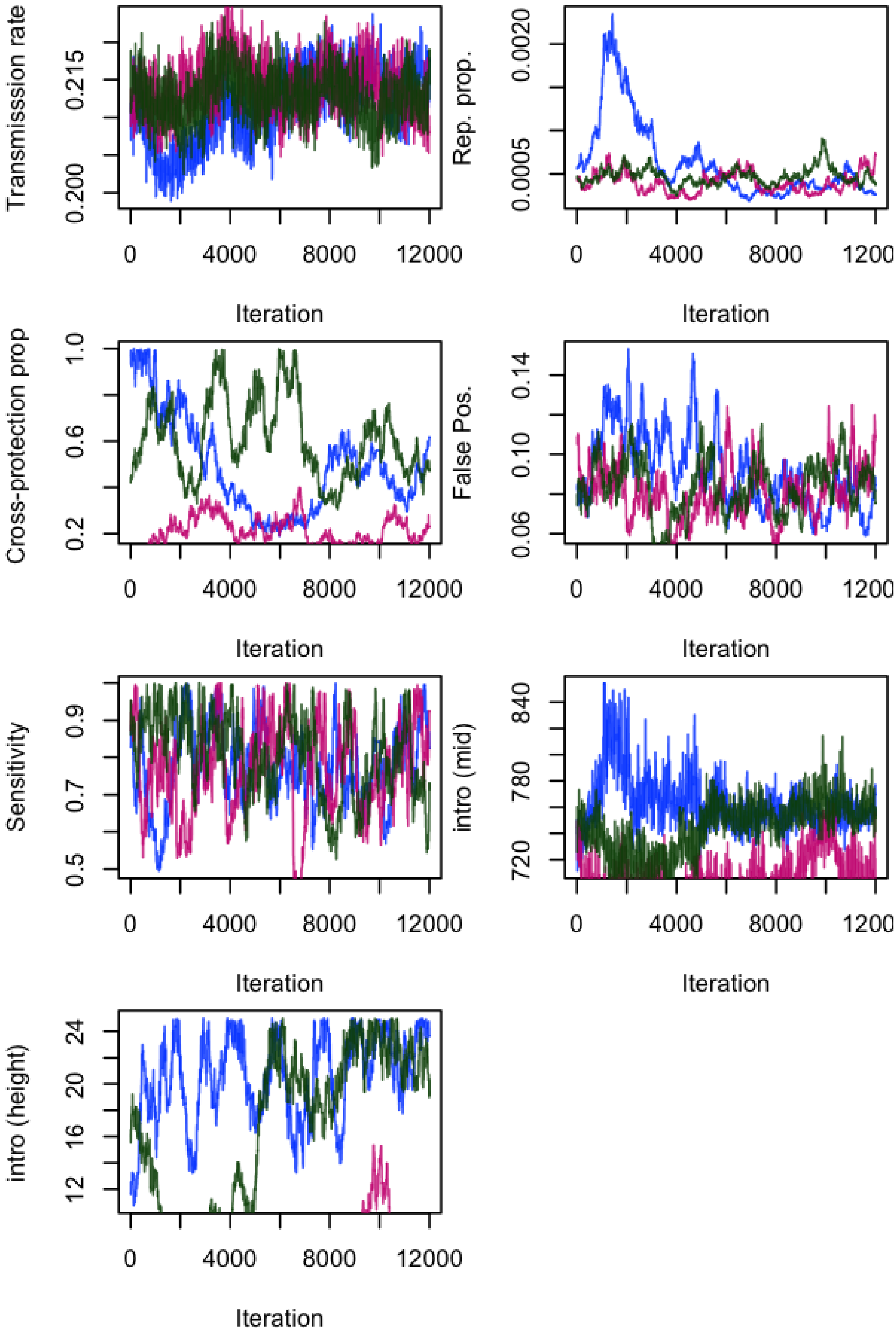

Supplementary Figure 19: *Trace plot of MCMC convergence for Model B after burn-in of 40%. The three colours represent three separate MCMC chains used in the fitting of the model to surveillance and serological data. A condensed plotting area is shown here so sometimes chains may not be visible, this is a sign of poor convergence*

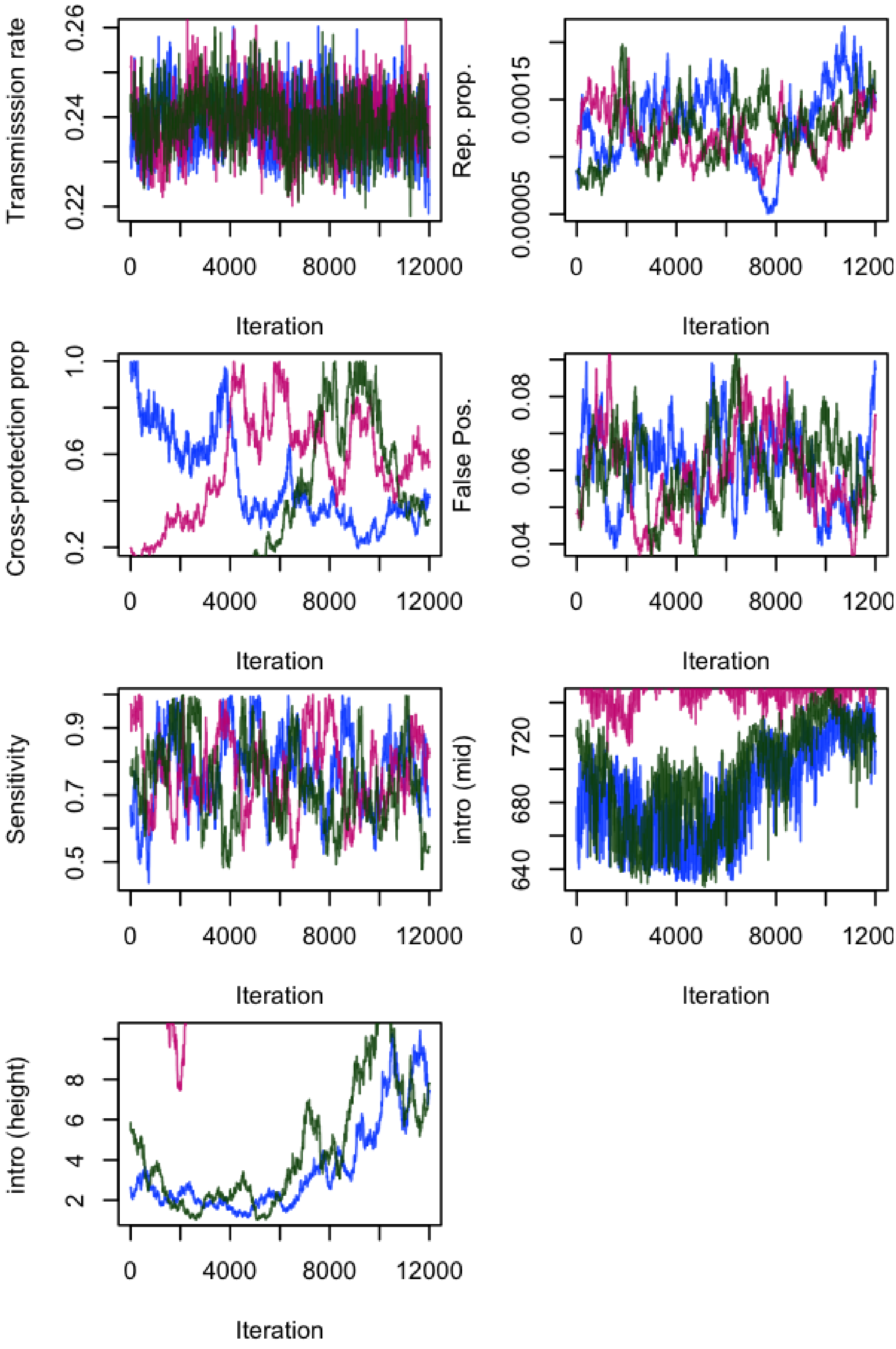

Supplementary Figure 20: *Trace plot of MCMC convergence for Model C after burn-in of 40%. The three colours represent three separate MCMC chains used in the fitting of the model to surveillance and serological data. A condensed plotting area is shown here so sometimes chains may not be visible, this is a sign of poor convergence*

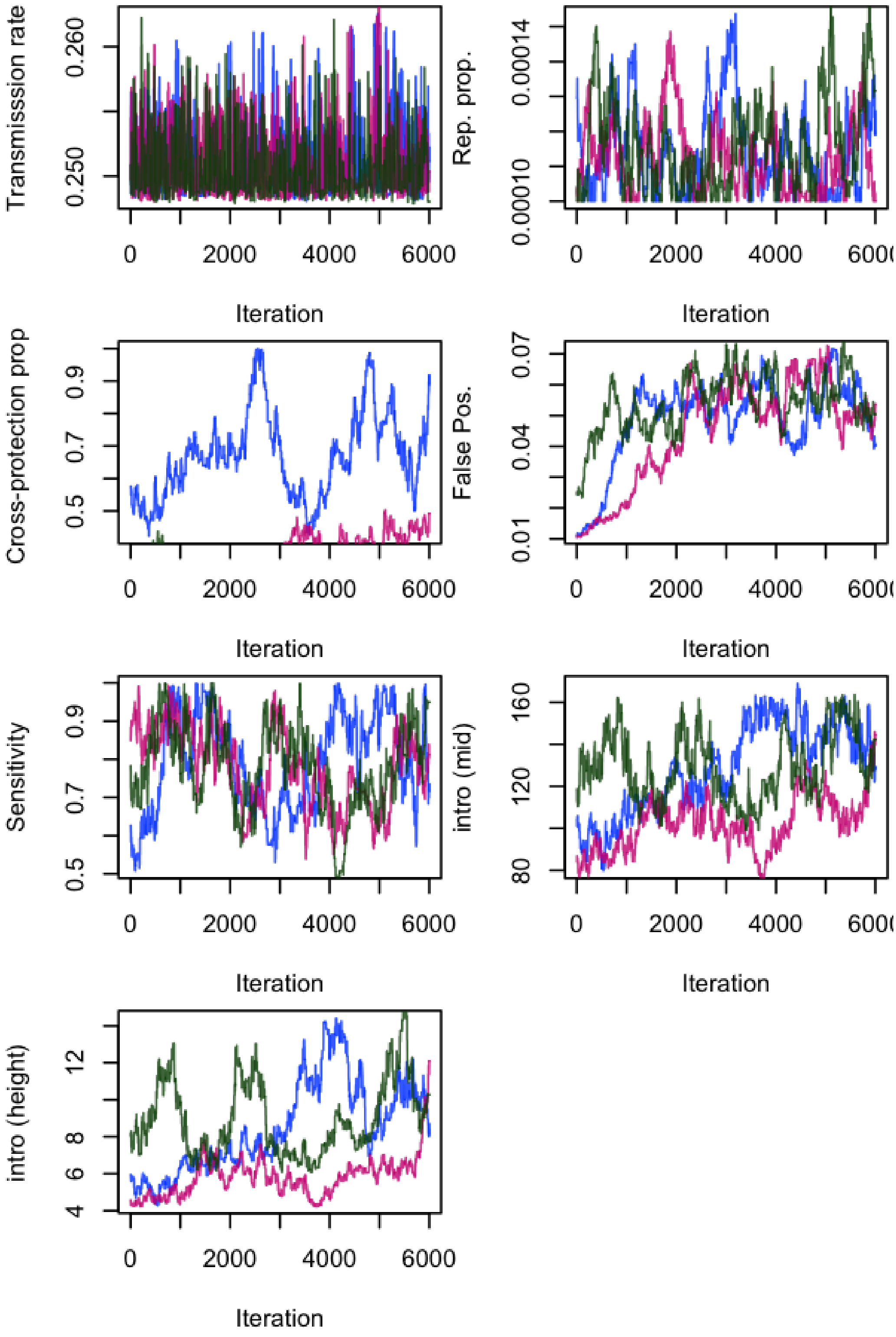

Supplementary Figure 21: *Trace plot of MCMC convergence for model D, constrained to an introduction in 2013 after burn-in of 40%. The three colours represent three separate MCMC chains used in the fitting of the model to surveillance and serological data. A condensed plotting area is shown here so sometimes chains may not be visible, this is a sign of poor convergence*

Supplementary References

[1] Fiji Bureau of Statistics. 2017 Population and Housing Census - Release 2. Technical report, 2018.

[2] Cameron P Simmons, Jeremy J Farrar, Nguyen van Vinh Chau, and Bridget Wills. Dengue. *New England Journal of Medicine*, 366(15):1423–1432, apr 2012. ISSN 0028-4793. doi: 10.1056/NEJMra1110265.

[3] Stefan Flasche, Mark Jit, Isabel Rodríguez-Barraquer, Laurent Coudeville, Mario Recker, Katia Koelle, George Milne, Thomas J Hladish, T Alex Perkins, Derek A T Cummings, Ilaria Dorigatti, Daniel J Laydon, Guido España, Joel Kelso, Ira Longini, Jose Lourenco, Carl A B Pearson, Robert C Reiner, Luis Mier-y Terán-Romero, Kirsten Vannice, and Neil Ferguson. The Long-Term Safety, Public Health Impact, and Cost-Effectiveness of Routine Vaccination with a Recombinant, Live-Attenuated Dengue Vaccine (Dengvaxia): A Model Comparison Study. *PLOS Medicine*, 13(11):e1002181, nov 2016.

[4] Fiji Bureau of Statistics. 2017 Population and Housing Census - Release 1. Technical report, 2018.

[5] OCHA's Regional Office for Asia and the Pacific. Fiji - Subnational Administrative Boundaries, 2018.

[6] Adam J. Kucharski, Mike Kama, Conall H. Watson, Maite Aubry, Sebastian Funk, Alasdair D. Henderson, Oliver J. Brady, Jessica Vanhomwegen, Jean Claude Manuguerra, Colleen L. Lau, W. John Edmunds, John Aaskov, Eric James Nilles, Van Mai Cao-Lormeau, Stéphane Hué, and Martin L. Hibberd. Using paired serology and surveillance data to quantify dengue transmission and control during a large outbreak in Fiji. *eLife*, 7, jan 2018. ISSN 2050084X. doi: 10.7554/eLife.34848.

[7] Conall H. Watson, Stephen Baker, Colleen L. Lau, Kitione Rawalai, Mere Taufa, Jerimaia Coriakula, Nga Tran Vu Thieu, Tan Trinh Van, Dung Tran Thi Ngoc, Niel Hens, John Lowry, Ruklanthi de Alwis, Jorge Cano, Kylie Jenkins, E. Kim Mulholland, Eric J. Nilles, Mike Kama, and W. John Edmunds. A cross-sectional seroepidemiological survey of typhoid fever in Fiji. *PLoS Neglected Tropical Diseases*, 11 (7):e0005786, jul 2017. ISSN 19352735. doi: 10.1371/journal.pntd.0005786.

[8] Colleen L. Lau, Conall H. Watson, John H. Lowry, Michael C. David, Scott B. Craig, Sarah J. Wynwood, Mike Kama, and Eric J. Nilles. Human Leptospirosis Infection in Fiji: An Eco-epidemiological Approach to Identifying Risk Factors and Environmental Drivers for Transmission. *PLoS Neglected Tropical Diseases*, 10(1), 2016. ISSN 19352735. doi: 10.1371/journal.pntd.0004405.

[9] Van Mai Cao-Lormeau, Alexandre Blake, Sandrine Mons, Stéphane Lastère, Claudine Roche, Jessica Vanhomwegen, Timothée Dub, Laure Baudouin, Anita Teissier, Philippe Larre, Anne Laure Vial, Christophe Decam, Valérie Choumet, Susan K. Halstead, Hugh J. Willison, Lucile Musset, Jean Claude Manuguerra, Philippe Despres, Emmanuel Fournier, Henri Pierre Mallet, Didier Musso, Arnaud Fontanet, Jean Neil, and Frédéric Ghawché. Guillain-Barré Syndrome outbreak associated with Zika virus infection in French Polynesia: A case-control study. *The Lancet*, 387(10027):1531–1539, 2016. ISSN 1474547X. doi: 10.1016/S0140-6736(16)00562-6.

[10] Maite Aubry, Anita Teissier, Michael Huart, Sébastien Merceron, Jessica Vanhomwegen, Claudine Roche, Anne Laure Vial, Sylvianne Teururai, Sébastien Sicard, Sylvie Paulous, Philippe Desprès, Jean Claude Manuguerra, Henri Pierre Mallet, Didier Musso, Xavier Deparis, and Van Mai Cao-Lormeau. Zika virus seroprevalence, French Polynesia, 2014–2015. *Emerging Infectious Diseases*, 23(4):669–672, 2017. ISSN 10806059. doi: 10.3201/eid2304.161549.

[11] Alasdair D. Henderson, Maite Aubry, Mike Kama, Jessica Vanhomwegen, Anita Teissier, Teheipuaura Mariteragi-Helle, Tuterarii Paoaafaite, Yoann Teissier, Jean Claude Manuguerra, John Edmunds, Jimmy

Whitworth, Conall H. Watson, Colleen L. Lau, Van Mai Cao-Lormeau, and Adam J. Kucharski. Zika seroprevalence declines and neutralizing antibodies wane in adults following outbreaks in french polynesia and fiji. *eLife*, 9, jan 2020. ISSN 2050084X. doi: 10.7554/eLife.48460.

[12] Cécile Beck, Philippe Desprès, Sylvie Paulous, Jessica Vanhomwegen, Steeve Lowenski, Norbert Nowotny, Benoit Durand, Annabelle Garnier, Sandra Blaise-Boisseau, Edouard Guitton, Takashi Yamanaka, Stéphan Zientara, and Sylvie Lecollinet. A High-Performance Multiplex Immunoassay for Serodiagnosis of Flavivirus-Associated Neurological Diseases in Horses. *BioMed Research International*, 2015:678084, 2015. ISSN 23146141. doi: 10.1155/2015/678084.

[13] Mike Kama, Maite Aubry, Taina Naivalu, Jessica Vanhomwegen, Teheipuaura Mariteragi-Helle, Anita Teissier, Tuterarii Paoaafaite, Stéphane Hué, Martin L. Hibberd, Jean Claude Manuguerra, Ketan Christi, Conall H. Watson, Eric J. Nilles, John Aaskov, Colleen L. Lau, Didier Musso, Adam J. Kucharski, and Van Mai Cao-Lormeau. Sustained low-level transmission of zika and chikungunya viruses after emergence in the Fiji islands. *Emerging Infectious Diseases*, 25(8):1535–1538, 2019. ISSN 10806059. doi: 10.3201/eid2508.180524.

[14] Stephen F. Altschul, Warren Gish, Webb Miller, Eugene W. Myers, and David J. Lipman. Basic local alignment search tool. *Journal of Molecular Biology*, 215(3):403–410, oct 1990. ISSN 00222836. doi: 10.1016/S0022-2836(05)80360-2.

[15] Erin A. Mordecai, Jeremy M. Cohen, Michelle V. Evans, Prithvi Gudapati, Leah R. Johnson, Catherine A. Lippi, Kerri Miazgowicz, Courtney C. Murdock, Jason R. Rohr, Sadie J. Ryan, Van Savage, Marta S. Shocket, Anna Stewart Ibarra, Matthew B. Thomas, and Daniel P. Weikel. Detecting the impact of temperature on transmission of Zika, dengue, and chikungunya using mechanistic models. *PLoS Neglected Tropical Diseases*, 11(4), 2017. ISSN 19352735. doi: 10.1371/journal.pntd.0005568.

[16] Elodie Descloux, Morgan Mangeas, Christophe Eugène Menkes, Matthieu Lengaigne, Anne Leroy, Temaui Tehei, Laurent Guillaumot, Magali Teurlai, Ann Claire Gourinat, Justus Benzler, Anne Pfannstiel, Jean Paul Grangeon, Nicolas Degallier, and Xavier de Lamballerie. Climate-based models for understanding and forecasting dengue epidemics. *PLoS Neglected Tropical Diseases*, 6(2), 2012. ISSN 19352727. doi: 10.1371/journal.pntd.0001470.

[17] Duane J Gubler. Dengue and Dengue Hemorrhagic Fever. *Clinical Microbiology Reviews*, 11(3):480–496, jul 1998. ISSN 0893-8512.

[18] Fiji Meteorological Service. Historical Meteorological Data Request Form, 2017.

[19] Alexei J. Drummond, Marc A. Suchard, Dong Xie, and Andrew Rambaut. Bayesian phylogenetics with BEAUti and the BEAST 1.7. *Molecular Biology and Evolution*, 29(8):1969–1973, feb 2012. ISSN 07374038. doi: 10.1093/molbev/mss075.

[20] Philippe Lemey, Andrew Rambaut, Alexei J Drummond, and Marc A Suchard. Bayesian Phylogeography Finds Its Roots. *PLoS Computational Biology*, 5(9):e1000520, 2009. ISSN 1553-7358. doi: 10.1371/journal.pcbi.1000520.

[21] Z Yang. A space-time process model for the evolution of DNA sequences. *Genetics*, 139(2):993–1005, 1995. ISSN 0016-6731.

[22] A. J. Drummond, A. Rambaut, B. Shapiro, and O. G. Pybus. Bayesian coalescent inference of past population dynamics from molecular sequences. *Molecular Biology and Evolution*, 22(5):1185–1192, feb

2005. ISSN 07374038. doi: 10.1093/molbev/msi103.

[23] Alexei J. Drummond, Simon Y.W. Ho, Matthew J. Phillips, and Andrew Rambaut. Relaxed phylogenetics and dating with confidence. *PLoS Biology*, 4(5):699–710, mar 2006. ISSN 15457885. doi: 10.1371/journal.pbio.0040088.

[24] Yong-Qiang Deng, Hui Zhao, Xiao-Feng Li, Na-Na Zhang, Zhong-Yu Liu, Tao Jiang, Da-Yong Gu, Lei Shi, Jian-An He, Hong-Jiang Wang, Zhao-Zeng Sun, Qing Ye, Dong-Yang Xie, Wu-Chun Cao, and Cheng-Feng Qin. Isolation, identification and genomic characterization of the Asian lineage Zika virus imported to China. *Science China Life Sciences*, 59(4):428–430, 2016. ISSN 1869-1889. doi: 10.1007/s11427-016-5043-4.

[25] Robert S Lanciotti, Olga L Kosoy, Janeen J Laven, Jason O Velez, Amy J Lambert, Alison J Johnson, Stephanie M Stanfield, and Mark R Duffy. Genetic and Serologic Properties of Zika Virus Associated with an Epidemic, Yap State, Micronesia, 2007. *Emerging Infectious Disease journal*, 14(8):1232, 2008. ISSN 1080-6059. doi: 10.3201/eid1408.080287.

[26] Anton Camacho, Sébastien Ballesteros, Andrea L. Graham, Fabrice Carrat, Oliver Ratmann, and Bernard Cazelles. Explaining rapid reinfections in multiplewave influenza outbreaks: Tristan da Cunha 1971 epidemic as a case study. *Proceedings of the Royal Society B: Biological Sciences*, 278(1725):3635–3643, 2011. ISSN 14712970. doi: 10.1098/rspb.2011.0300.

[27] H J Wearing, P Rohani, and M J Keeling. Appropriate models for the management of infectious diseases. *PLoS Medicine*, 2, 2005.

[28] Toscane Fourie, Gilda Grard, Isabelle Leparc-Goffart, Sebastien Briolant, and Albin Fontaine. Variability of zika virus incubation period in humans. *Open Forum Infectious Diseases*, 5(11), 2018. ISSN 23288957. doi: 10.1093/ofid/ofy261.

[29] Veasna Duong, Louis Lambrechts, Richard E Paul, Sowath Ly, Rath Srey Lay, Kanya C Long, Rekol Huy, Arnaud Tarantola, Thomas W Scott, Anavaj Sakuntabhai, and Philippe Buchy. Asymptomatic humans transmit dengue virus to mosquitoes. *Proceedings of the National Academy of Sciences of the United States of America*, 112(47):14688–93, 2015. ISSN 1091-6490. doi: 10.1073/pnas.1508114112.

[30] Matthew H. Collins, Eileen McGowan, Ramesh Jadi, Ellen Young, Cesar A. Lopez, Ralph S. Baric, Helen M. Lazear, and Aravinda M. de Silva. Lack of durable cross-neutralizing antibodies against zika virus from dengue virus infection. *Emerging Infectious Diseases*, 23(5):773–781, 2017. ISSN 10806059. doi: 10.3201/eid2305.161630.

[31] The World Bank. Life expectancy at birth, total (years) - Fiji, 2020.

[32] The World Bank. Birth rate, crude (per 1,000 people) - Fiji, 2020.

[33] The World Bank. Death rate, crude (per 1,000 people) - Fiji, 2020.

[34] Matt J. Keeling and Pejman Rohani. *Modeling infectious diseases in humans and animals*. 2011. ISBN 9781400841035. doi: 10.1016/s1473-3099(08)70147-6.

[35] Gareth O. Roberts and Jeffrey S. Rosenthal. Examples of adaptive MCMC. *Journal of Computational and Graphical Statistics*, 18(2):349–367, 2009. ISSN 10618600. doi: 10.1198/jcgs.2009.06134.

[36] R R Development Core Team. *R: A Language and Environment for Statistical Computing*, volume 1. 2011. ISBN 3900051070. doi: 10.1007/978-3-540-74686-7.

[37] RStudio Team. RStudio: Integrated development environment for R, 2012. ISSN 0022541X.

[38] A. Genz, F. Bretz, T. Miwa, X. Mi, F. Leisch, F. Scheipl, B. Bornkamp, M. Maechler, and T. Hothorn.

Package ‘ mvtnorm ’, 2015.

[39] Karline Soetaert, Thomas Petzoldt, and R. Woodrow Setzer. Solving differential equations in R: Package deSolve. *Journal of Statistical Software*, 33(9):1–25, 2010. ISSN 15487660. doi: 10.18637/jss.v033.i09.

[40] Revolution Analytics. doMC: Foreach parallel adaptor for the multicore package, 2013.

[41] Alasdair Henderson. a-henderson91/fiji-zikv-model: Include corrections from thesis, February 2021.

[42] MRC Biostatistics Unit. DIC: Deviance Information Criteria, 2020.
