# Supplementary material for: Interactions between timing and transmissibility explain diverse flavivirus dynamics in Fiji
Source: Nat Commun. 2021 Mar 15;12:1671. doi: 10.1038/s41467-021-21788-y (PMC7961049; doi:10.1038/s41467-021-21788-y)
Supplement: Supplementary file 3 — Reporting Summary [file 41467_2021_21788_MOESM3_ESM.pdf]

nature research

Corresponding author(s): Alasdair Henderson

Last updated by author(s): Feb 3, 2021

Reporting Summary

Nature Research wishes to improve the reproducibility of the work that we publish. This form provides structure for consistency and transparency in reporting. For further information on Nature Research policies, see our Editorial Policies and the Editorial Policy Checklist.

Statistics

For all statistical analyses, confirm that the following items are present in the figure legend, table legend, main text, or Methods section.

n/a	Confirmed	
☐	☒	The exact sample size (*n*) for each experimental group/condition, given as a discrete number and unit of measurement
☐	☒	A statement on whether measurements were taken from distinct samples or whether the same sample was measured repeatedly
☒	☐	The statistical test(s) used AND whether they are one- or two-sided<br>*Only common tests should be described solely by name; describe more complex techniques in the Methods section.*
☒	☐	A description of all covariates tested
☐	☒	A description of any assumptions or corrections, such as tests of normality and adjustment for multiple comparisons
☐	☒	A full description of the statistical parameters including central tendency (e.g. means) or other basic estimates (e.g. regression coefficient) AND variation (e.g. standard deviation) or associated estimates of uncertainty (e.g. confidence intervals)
☒	☐	For null hypothesis testing, the test statistic (e.g. *F*, *t*, *r*) with confidence intervals, effect sizes, degrees of freedom and *P* value noted<br>*Give P values as exact values whenever suitable.*
☐	☒	For Bayesian analysis, information on the choice of priors and Markov chain Monte Carlo settings
☒	☐	For hierarchical and complex designs, identification of the appropriate level for tests and full reporting of outcomes
☒	☐	Estimates of effect sizes (e.g. Cohen's *d*, Pearson's *r*), indicating how they were calculated

Our web collection on statistics for biologists contains articles on many of the points above.

Software and code

Policy information about availability of computer code

Data collection	Microsoft Excel (version 16.44) was used for data entry. R (version 4.0.2) was used to clean and process data.
Data analysis	All models were implemented in R version 4.0.2 using the mvtnorm (version 1.1), truncnorm (version 1.0.8) and deSolve (version 1.28) packages and parallelised using the doMC (version 1.3.6) package. An MCMC sampler was written in R to fit the mathematical model to multiple data sources using tidyverse (version 1.3.0). Figures were created with the ggplot2 package (version 3.3.2). BEAST and BEAUTi (version 1.10.4) were used to analyse sequence data. All code used in this analysis are available at https://github.com/a-henderson91/fiji-zikv-model (DOI: 10.5281/zenodo.4487358).

For manuscripts utilizing custom algorithms or software that are central to the research but not yet described in published literature, software must be made available to editors and reviewers. We strongly encourage code deposition in a community repository (e.g. GitHub). See the Nature Research guidelines for submitting code & software for further information.

Data

Policy information about availability of data

All manuscripts must include a data availability statement. This statement should provide the following information, where applicable:

- Accession codes, unique identifiers, or web links for publicly available datasets
- A list of figures that have associated raw data
- A description of any restrictions on data availability

The surveillance, serological and sequence data used in this study have been deposited in GitHub (DOI:10.5281/zenodo.4487358).

Field-specific reporting

Please select the one below that is the best fit for your research. If you are not sure, read the appropriate sections before making your selection.

- [x] Life sciences
- [ ] Behavioural & social sciences
- [ ] Ecological, evolutionary & environmental sciences

For a reference copy of the document with all sections, see nature.com/documents/nr-reporting-summary-flat.pdf

Life sciences study design

All studies must disclose on these points even when the disclosure is negative.

Sample size	For the serological data, we aimed to follow up 350 participants in 2017 as this was approximately 50% of the original 2013 study participants in 2017. We assumed that approximately 15% of these paired samples would seroconvert between 2015 and 2017. Allowing for 5% seroreversion, assuming no cross-reactivity and a probability of type-1 error of 0.05, with a sample size of 350 we would be able to detect a 15% change in ZIKV seroprevalence between 2015 and 2017 with 88% power using McNemar's test, and a 20% change with 98% power.
Data exclusions	Participants were eligible for inclusion if they were aged 12 months or older during the first serological data collection in 2013. The youngest possible participants for inclusion would be at least 3 by May 2017. Exclusion criteria were clotting disorders, such as haemophilia and other coagulopathies, concurrent medical anticoagulation such as through administration of warfarin or heparin, or the presence of severe underlying medical conditions or significant acute illness. Non-medical exclusion criteria were needlephobia or other unwillingness to participate, inability to consent to treatment through lack of insight, understanding, and for children, the refusal of, or inability to attain parental consent. There were no additional exclusion criteria. These criteria were checked again in 2017 to confirm that the potential participant was still eligible.
Replication	Our findings are dependent on the timing of sample collection and it is therefore not possible to reproduce these findings as described in our study. The modeling and model fitting procedures have been described in detail so that the results may be reproduced with the same data.
Randomization	Our longitudinal serological study did not separate groups by exposure status. We tested for the evidence of previous infection with Zika or dengue virus in the general population. Randomization was therefore unnecessary for the purposes of our study.
Blinding	There was no group allocation as this was a study in the general population. Blinding was therefore unnecessary for the purposes of our study.

Reporting for specific materials, systems and methods

We require information from authors about some types of materials, experimental systems and methods used in many studies. Here, indicate whether each material, system or method listed is relevant to your study. If you are not sure if a list item applies to your research, read the appropriate section before selecting a response.

Materials & experimental systems

n/a	Involved in the study	
x		Antibodies
x		Eukaryotic cell lines
x		Palaeontology and archaeology
x		Animals and other organisms
	x	Human research participants
x		Clinical data
x		Dual use research of concern

Methods

n/a	Involved in the study	
x		ChIP-seq
x		Flow cytometry
x		MRI-based neuroimaging

Human research participants

Policy information about studies involving human research participants

Population characteristics	The population has been previously described in full (Henderson et al., Zika seroprevalence declines and neutralizing antibodies wane in adults following outbreaks in French Polynesia and Fiji, eLife, 2020).
Recruitment	In 2013, nursing zones were randomly selected, from which one individual from 25 households in a randomly selected community was recruited. Participants who had consented to being contacted again for health research were subsequently recruited in November 2015 in 23 communities in Central Division through last known addresses, phone numbers and the assistance of local nurses (n=327). A third follow-up serosurvey was conducted in June 2017 using the same protocol as in 2015 (n=321).
Ethics oversight	The original 2013 study, and the 2015 and 2017 follow up studies were approved by the Fiji National Research Ethics Review Committee (ref 2013–03, 2015.111.C.D, 2017.20.MC) and the London School of Hygiene and Tropical Medicine Observational Research Ethics Committee (ref 6344, 10207, 12037).

Note that full information on the approval of the study protocol must also be provided in the manuscript.
