# Supplementary material for: Interactions between timing and transmissibility explain diverse flavivirus dynamics in Fiji
Source: Nat Commun. 2021 Mar 15;12:1671. doi: 10.1038/s41467-021-21788-y (PMC7961049; doi:10.1038/s41467-021-21788-y)
Supplement: Supplementary file 2 — Peer Review File [file 41467_2021_21788_MOESM2_ESM.pdf]

REVIEWER COMMENTS

Reviewer #1 (Remarks to the Author):

In this manuscript, Henderson and colleagues combined multiple datasets, including surveillance cases, serology, meteorological data, and molecular sequencing data, with a mathematical transmission model to reconstruct complex hidden transmission dynamics of ZIKV in the Central Division of Fiji during 2013-18. They successfully revealed the influence of virus introduction time on subsequent ZIKV outbreaks, annual optimal time window in which the seeding of ZIKV was easier due to ecological factors, and weak model support to the cross-protection between dengue outbreak in 2014 and subsequent ZIKV outbreaks. In my opinion, their topic is of interest to the audiences of Nature Communications, with sound method and presentation.

I have a few comments:
(1) The relationship between temperature and relative R0 is based on an existing study (Ref. [12]). This relationship of seasonality is used to inform the force of infection (FOI) Eq. (5)-(6) in supplementary.
Is it possible to infer the time-varying FOI directly from surveillance case or serological data? The catalytic model in this paper may be helpful: "Time-varying, serotype-specific force of infection of dengue virus, PNAS (2014) https://www.pnas.org/content/111/26/E2694"
I would like to suggest the authors to compare the time-varying FOI estimated with temperature data and the time-varying FOI estimated with case data or serological data. This will allow more solid assumptions on the FOI, and possibly more robust reconstruction of ZIKV outbreaks.

(2) Demographic change (e.g., birth/death) has been excluded in the math model?

(3) For cross-reactive immune response between dengue and ZIKV, the authors mainly considered the temporal cross-protection. Is it possible to also explore the influence of antibody-dependent enhancement between dengue and ZIKV infections?

(4) Line 747 – 749, the duration of cross-protection from DENV-3 is fixed to 30 days? This duration may be too short for the waning of cross-reactive dengue antibodies. Probably good to try 6 months.

(5) Page 29 (below Eq. (1) in supplement), "The sine function 1 was fit to daily maximum temperature data". Do you mean "fit to daily average temperature data"?

(6) Line 734 page 33, "follow an Erlang, not exponential, distribution [51]". It's good to also cite this paper: "Appropriate Models for the Management of Infectious Diseases, Plos Med (2005) https://journals.plos.org/plosmedicine/article?id=10.1371/journal.pmed.0020174"

(7) Why Erlang distribution is more suitable than exponential distribution to describe the waning of antibodies and hence reduced cross-protection effect?

(8) Line 744 page 34, "of the intrinsic period in humans". Do you mean the intrinsic incubation period?

(9) Line 775, the dispersion parameter is also fitted from the model?

Review

Interactions between timing and transmissibility explain diverse flavivirus dynamics in Fiji

NCOMMS-20-22353

Summary

The authors developed a model to understand what factors drive different transmission dynamics of DENV-3 and ZIKV in Fiji. They present nice use of data from different sources using a range of rigorous Bayesian methods and a mathematical model of transmission to explore introduction times, reporting proportions and waning seropositivity in people exposed to ZIKV. The figures are general clear and informative in explaining the methods used (Schematic 4 in the SI is very illuminating) and results (e.g. Figs 2 and 3); Figure 3 shows how much difference the timing of new infections ZIKV imports can make – with the possibility to have anything from one major peak to two similar sized peaks.

I have a list of comments (attached) which would improve the readability of the figures, and improve the technical presentation of material in the SI. Most of my major comments related to clarifying the model equations (main model and log-likelihood function).

The subject itself and use of modelling methods to better understand unobservable transmission events would be very appealing to readers interested in a broad range of infections.

Specific comments:

Page	line	Comment
3	14	Please mention that seroprevalance is based on an antibody test and so a measure of recent exposure rather than current infection. I know you discuss this later, but it is important for the readers understanding early on.
4	40	“fit” should be “fitted” (same on page 15 line 272 and others)
5	Fig 1	Some of the colours here are a bit hard to read. I suggest the yellow and light grey in C should be darker and some of the font in B is extremely small. I’m a bit confused by the pink line (tMRCA) going between B and C and many different axes sitting inside each other. It took me a while to work out the grey box B was the insert
6	Fig 2	Can you make the label “reproduction number” pink/red on Fig2C and “average temperature” label grey to make this more intuitive to read. Is the red line still the effective reproduction number of ZIKV, not DENV-3? I assume Suva is in the Central Divison?
9	Fig 3	Can you make the introductions and infections red and blue rather than saying (red) or (blue) in the label? This would be consistent with your other figures. I would find it more intuitive to have introductions on the LHS axis and infections on the RHS axis as introductions is the piece of information you’re look for first.

		The attack rate requires some further explanation – this is the total number of individuals would become infected if infection were introduced at different times?<br><br>Your main results show three peaks – when do you have to introduce cases to generate those dynamics?<br><br>Can you mention the population size of the Central Division in the main text (your SI says 342K)? For example the number of infections in Figure 3 are hard to interpret without this information. It could be part of the figure caption.
10	Discussion	What is the justification for using a deterministic transmission model when there were so few ZIKV cases? Would we expect persistence for several years in an equivalent stochastic formulation? There is no mention your model is deterministic until you read the SI, I would be more upfront about this.
10	136	In the discussion you mention how it's likely that there is a single introduction event, however you seen to contradict this in the SI (line 709) when you say that phi(t) "better represents real introduction dynamics rather than a single introduction event". I assume you mean there was a single wave of introductions rather than multiple events or waves?
15	274	"phulogenetic" should be "phylogenetic"
26	592	Should your specificity for ZIKV not change through time if DENV-3 prevalence changes and impacts the false positive rate?
27	631	Perhaps keep consistent and use of "I" instead of "we" (similar occurrences elsewhere in SI). You need to state the name of the first author (et al) as well as reference number (12).
30	667	Missing space between "study," and "we"
34	740	"clean-up" and "clean up" are used interchangeably. Please be consistent
34	Model	Why have you used partial derivative notation for the ODEs?<br><br>"I" should be "I_Z" (equations 13/14)<br><br>Why is dengue not dependent on mosquitoes or seasonality? There is only a constant force of infection of beta_D?<br><br>Mosquito population size: You don't say explicitly, but s_M, e_M, i_M are proportions. Your relative vector to host ratio should feature somewhere in R_0 though and at the moment it doesn't appear to be in your equations explicitly. I may have missed some non-dimensionalisation somewhere (and it might be contained as part of your lambdas/betas).<br><br>Although it won't make any ultimate difference to the final computed value of R_0, I think your R_ZM term shouldn't have the

		N in the dominator, and R_MZ should have it there (based on equations 8 and 14).<br><br>Can you give a biological interpretation for beta_M and beta_Z in terms of bite rates, probability of infection and vector population size? This would help understand why your prior for beta_Z is between 0 and 1, whilst your beta_M prior can take any positive value.<br><br>What happens to people transitioning out of the R_Z box? Do they continue to remain uninfected and immune to both ZIKV and DENV-3? You talk about DENV-3 conferring immunity to ZIKV but not the other way around. Please provide some brief note on this, including why ZIKV immunity is not waning, whilst DENV-3 immunity is.
34	Model	You don't explain C_Z(t) in the text around equation 11. I assume this counts the number of new infections at any given time? Later on (774) you use c_t=C_t-C_(t-1) – should this be C_Z(t)-C_Z(t-1)? This would make more sense to me.
37	Eq 24	You need to decide whether c_t is the number of new infections occurring or reported in the model. If it is occurring, your equation on line 774 makes sense, but equation 24 need to have an "r" in it for reporting. If it is reported (as per your text description on line 773) then c_t is not C_Z(t)-C_Z(t-1) but c_t=r(C_t-C_(t-1)).<br><br>I would have expected the serological data to be linked to R_Z(t) as this has the waning ZIKV antibodies component. Equation 24 uses C_j (which I assume is actually C_Z(j)?) which is the cumulative number of infected individuals rather than the ones with antibodies?
38	797	Rather than using arbitrary differences in DIC score which are very dependent on the data and set up of the log-likelihood function, can you compute the relative DIC score.<br><br>Also please provide a reference for your selected DIC equation. There are many different variants and a brief justification of why you used this DIC would be helpful (although I am pleased you explicitly stated your calculation).
39		This SI has provided all the statistical outputs I would expect to see for the main model. Being critical – I would expect better mixing for the beta parameters after burn-in. I do appreciate showing the two chains and diagnostic plots.<br><br>Do you have trace plots for Models B and C?
SI		I would find it very informative to see a map in SI of Fiji and central division

Reviewer #3 (Remarks to the Author):

This is an original research study which analyses data from a Zika virus outbreak in Fiji using mathematical models. There were 16 confirmed cases over a 2-year period. In contrast, two years earlier, a dengue virus outbreak had caused over 12,000 suspected cases over a 9-month period. Given the close relatedness of the two viruses and their common route of vector-borne transmission, the discrepancy between the two outbreaks was unexpected. Elucidating the underlying differences is the main motivation for this study. The authors implemented several alternative hypotheses and mechanisms in their models, which were fitted to the data. In addition to case reports, three serosurveys, conducted before, during and after the outbreak, were available, as well as sequences from a small number of isolates. The authors' lead hypothesis, which was confirmed by their analysis, was that the timing of the initial virus introduction was critical in determining the size of the outbreak due to seasonal variations in the mosquito vector population. They also tested a model where cross-immunity from the previous dengue outbreak reduced the population's susceptibility to Zika, but found little statistical support.

I found the general approach interesting, especially the combination of case reports, serology and sequencing to draw inference on the outbreak dynamics. However, I have some concerns about the model structure as well as some of the conclusions and I think the manuscript requires substantial revisions before its validity and value can be properly assessed.

First of all, I am not convinced that the study does much to address the main question laid out in the introduction: why were zika case reports three orders of magnitude lower than the previous dengue outbreak? Although it is implied that confirmed cases are only a fraction of the total number of infections, I didn't see any explicit results or discussion of this issue. The SEIR model does not distinguish between infected individuals who may test positive or exhibit symptoms, and I had to dig into the appendix (line 774) to find the briefest mention of a reporting rate r, whose prior and posterior are nowhere to be seen. The simulations shown on Figure 3 suggest (but again that's not explained by the authors) that the Zika epidemic actually infected thousands of people (although the actual value is hard to read from a continuous prevalence curve). The panels in Figure 2 indicate that the effect of the timing of introduction can affect the epidemic size by roughly a factor 2 at most (less if you add the two successive epidemic peaks). I cannot reconcile these small fluctuations with the 1,000-fold difference between the case reports for dengue and Zika as shown on Figure 1 C. I can only assume that most of it is driven by different case to infection ratios, with timing of introduction only playing a marginal role. As a result, I'm not sure what can really be learned from this study.

Turning to the model structure described in the appendix, I have several queries:
- First, I was surprised that a deterministic model was used given the very low number of cases. Even though I then realised that the numbers of infections were assumed to be much higher than the PCR-confirmed cases, there is still the potential for stochastic fadeouts between seasons. Deterministic models are known to generate clockwork epidemic cycles which can be very different from reality. A stochastic model would also be helpful for the introduction process, which involves very low numbers too.
- I'm very confused by the way the DENV epidemic was modelled. The equations on page 34 do not match the diagram on Figure 8. I don't understand why individuals go from S_Z to S_D upon exposure to dengue (why not go to E_D?). In any case, the corresponding term is missing from equation 17 (or 18). Then the DENV infection rate appears to imply that \beta_D is the force of infection and is a constant over the epidemic, with no apparent effect of mosquito infection or seasonality, both of which are surprising. It's also a bit strange that, given all these and other approximations, the authors deemed it necessary to use an Ehrlang distribution (just with 2 sub-compartments) for the duration of cross-protection.
- Given that simulations are run over several years, the lack of birth and death in the human population is an issue.
- Table 2 on page 36 shows a "test specificity" parameter with a prior set at U(0,1) which is quite

surprising: was there no prior evidence? More surprisingly, there does not appear to be a sensitivity parameter. Given the very low number of PCR-positive cases reported, I would expect the sensitivity rate to be crucial for inference.

Lastly, I was confused by the way the interaction between ZIKV and DENV were assessed statistically. The Results section on page 8 appears to be testing the statistical support for an early introduction of ZIKV, which seems to be a very specific scenario. Did the authors seek to assess the statistical support for cross-immunity more generally?

In conclusion, I think this study has a lot of potential to be interesting and relevant, but there are just too many grey areas and gaps in the current manuscript.

Response to reviewers

Having read the reviewer comments we agree that technical changes to the model were necessary and that the manuscript would benefit from greater clarity in the communication of results and presentation of the figures. A summary of the major revisions is described here followed by a point-by-point response to reviewer comments.

Major revisions:

- *The model has been simplified to reduce the number of estimated parameters and improve mixing and model convergence. The mosquito population is no longer directly modelled and both the Zika and dengue outbreak are modelled in an SEIR framework. However, demographic changes in the population are now included.*

- *The differences in reporting proportions are discussed explicitly in the manuscript and in greater detail in the supplementary information. This was a primary cause of the diverse observed outbreaks in case data and deserved greater importance in the manuscript.*

- *Both sensitivity and specificity of the serological assay are now estimated when comparing model outputs to the seroprevalence estimates.*

- *The use of a deterministic framework is discussed in greater detail. We show that the number of infections in the model when the effective reproduction number falls below 1 between epidemic seasons is large enough for the epidemic to persist over multiple years. We also explicitly remove the possibility of epidemic emergence when the effective reproduction number is less than 1.*

Greater detail on these revisions are provided in the response to reviewer comments below.

REVIEWER 1
1.1 The relationship between temperature and relative R0 is based on an existing study (Ref. [12]). This relationship of seasonality is used to inform the force of infection (FOI) Eq. (5)-(6) in supplementary. Is it possible to infer the time-varying FOI directly from surveillance case or serological data? The catalytic model in this paper may be helpful: “Time-varying, serotype-specific force of infection of dengue virus, PNAS (2014)”

I would like to suggest the authors to compare the time-varying FOI estimated with temperature data and the time-varying FOI estimated with case data or serological data. This will allow more solid assumptions on the FOI, and possibly more robust reconstruction of ZIKV outbreaks.

We agree that this would be an interesting method to apply but unfortunately it is not possible in our study because of the size of our serological surveys. The study cited by the reviewer and similar studies (Salje et al., Reconstruction of 60 Years of Chikungunya Epidemiology in the Philippines Demonstrates Episodic and Focal Transmission, JID (2015)) estimate time-varying force of infection from much larger serological sample sizes or samples collected over a longer time period than were available from our study. In 2017 we

had individual samples from 320 people which would be insufficient to estimate a time-varying force of infection for historical epidemics with any degree of precision. Given the sporadic nature of dengue epidemics in Fiji, there is also the issue of generalisablity of dynamics of outbreaks that occurred decades ago. For these reasons, we focused on more recent epidemics, combining serological data with surveillance and molecular data to infer the outbreak dynamics of ZIKV in Fiji, using the 2013/14 dengue epidemic to calibrate out model.

1.2 Demographic change (e.g., birth/death) has been excluded in the math model?

We agree that this was an important omission and now include demographic change in the model. Full details are provided in the methods (line 293) and supplementary information (SI) (lines 824-827, Figure 10, Equations 6-9).

1.3 For cross-reactive immune response between dengue and ZIKV, the authors mainly considered the temporal cross-protection. Is it possible to also explore the influence of antibody-dependent enhancement between dengue and ZIKV infections?

There is mixed evidence about the effect of previous DENV infection on subsequent ZIKV infection and whether it enhances or protects from serious disease. There is however evidence of ADE in the opposite direction, ZIKV infection enhancing subsequent DENV infection (https://science.sciencemag.org/content/369/6507/1123.abstract). We excluded the possibility of antibody-dependent enhancement between DENV and ZIKV because there were so few ZIKV cases in Fiji following a recent DENV-3 outbreak. However, we agree that the manuscript would benefit by making this explicit.

We have added the following to the methods (line 288):

> *We excluded antibody-dependent enhancement between DENV and ZIKV from our model as this would result in an increase in reported ZIKV cases and a comparison of our surveillance and serological data showed that very few infections were recorded as cases of ZIKV.*

1.4 Line 747 – 749, the duration of cross-protection from DENV-3 is fixed to 30 days? This duration may be too short for the waning of cross-reactive dengue antibodies. Probably good to try 6 months.

We agree that this is a conservative choice for the duration of cross-protective antibodies following DENV-3 infection. We ran the model with 6-month protection and found similar results that are now included in the SI (line 965). Early introduction of ZIKV remains poorly supported by our model and this finding is robust to different lengths for the duration of cross-protection.

1.5 Page 29 (below Eq. (1) in supplement), “The sine function 1 was fit to daily maximum temperature data”. Do you mean “fit to daily average temperature data”?

Thank you, this was an error and has been corrected (line 713).

1.6 Line 734 page 33, “follow an Erlang, not exponential, distribution [51]”. It’s good to also cite this paper: “Appropriate Models for the Management of Infectious Diseases, Plos Med (2005)
https://journals.plos.org/plosmedicine/article?id=10.1371/journal.pmed.0020174”
1.7 Why Erlang distribution is more suitable than exponential distribution to describe the waning of antibodies and hence reduced cross-protection effect?

We thank the reviewer and the citation has been included. The explanation of the choice of Erlang distributed cross-protective period now includes the following (line 802):

> *Two compartments were used so that the duration to return to susceptible would follow an Erlang, not exponential, distribution (Camacho et al., 2011. Wearing et al., 2015). Using an exponential distribution means that there is nonzero density for the null duration in a compartment, so some people can transition artificially rapidly. Using an Erlang distribution solves this problem and is particularly valuable when the average duration of in a compartment is long, as it is between R_D and S_Z.*

1.8 Line 744 page 34, “of the intrinsic period in humans”. Do you mean the intrinsic incubation period?

Thank you, this was an error and has been corrected (line 813).

1.9 Line 775, the dispersion parameter is also fitted from the model?

The following is included in the SI (857):

> *The dispersion parameter ϕ adjusted for the inequality between mean and variance in the case data. Since this was not a parameter of particular epidemiological interest for our study, we fixed the value to improve the mixing and convergence of other more significant parameters.*

REVIEWER 2:
2.1 Please mention that seroprevalence is based on an antibody test and so a measure of recent exposure rather than current infection. I know you discuss this later, but it is important for the readers understanding early on.

We have changed line 14 in the introduction to make this explicit:

> *Data from a longitudinal community serological survey found an increase from 7.8 seroprevalence for anti-ZIKV immunoglobulin class G (IgG) antibodies in November 2013 to 21.9% in November 2015.*

2.2 “fit” should be “fitted” (same on page 15 line 272 and others)

Thank you, this has been corrected throughout the manuscript.

2.5 (Figure 1) Some of the colours here are a bit hard to read. I suggest the yellow and light grey in C should be darker and some of the font in B is extremely small. I'm a bit confused by the pink line (tMRCA) going between B and C and many different axes sitting inside each other. It took me a while to work out the grey box B was the insert
2.6 (Figure 2) Can you make the label "reproduction number" pink/red on Fig2C and "average temperature" label grey to make this more intuitive to read. Is the red line still the effective reproduction number of ZIKV, not DENV-3? I assume Suva is in the Central Division?
2.7 (Figure 3) Can you make the introductions and infections red and blue rather than saying (red) or (blue) in the label? This would be consistent with your other figures. I would find it more intuitive to have introductions on the LHS axis and infections on the RHS axis as introductions is the piece of information you're look for first. The attack rate requires some further explanation – this is the total number of individuals would become infected if infection were introduced at different times?

Your main results show three peaks – when do you have to introduce cases to generate those dynamics? Can you mention the population size of the Central Division in the main text (your SI says 342K)? For example the number of infections in Figure 3 are hard to interpret without this information. It could be part of the figure caption.

Thank you for the detailed feedback on the presentation of figures. All comments have been adopted into the new Figures 1-3 and captions in the amended manuscript.

2.8 What is the justification for using a deterministic transmission model when there were so few ZIKV cases? Would we expect persistence for several years in an equivalent stochastic formulation? There is no mention your model is deterministic until you read the SI, I would be more upfront about this.

We agree that this is a good point and needed addressing in our model and manuscript.

We agree that a deterministic model can produce implausible dynamics if infections are introduced into the population during periods where the reproduction number is low, or generate artificially cyclical outbreaks if infection declines to implausibly low levels between outbreaks. The following has been added to the model and is described in the methods section (line 304):

> *To better reflect reality, we included two conditions explicitly in the model. Firstly, there had to be at least one infectious individual for the virus to transmit. Secondly, we set the number introduced to zero if the effective reproduction number was below 1. This prevents epidemic take-off and implausible points of the year.*

These changes are described in the methods section, the supporting information and we have included the following paragraph in the discussion about these changes and the limitations of deterministic model (line 177):

> *We used a deterministic framework to model ZIKV which is limited because it could produce artificially cyclical outbreaks that do not reflect reality. There were two*

potential problems deterministic models can generate that we explicitly guarded against. With strong seasonal forcing in transmission it is possible that epidemics can reduce to implausibly low levels to persist over colder months before re-emerging. Our model included a condition that there was at least one infected person for the virus to transmit. Regardless we estimated that the number infected over colder months was consistently above 100 during lower transmission seasons (Figure 2C). Secondly, to avoid epidemics emerging in our model at implausible times of the year. To protect against this, we explicitly prevented introductions and a possible epidemic take off when R was below 1.

2.9 In the discussion you mention how it's likely that there is a single introduction event, however you seen to contradict this in the SI (line 709) when you say that phi(t) "better represents real introduction dynamics rather than a single introduction event". I assume you mean there was a single wave of introductions rather than multiple events or waves?

We agree that this is an important distinction. The following has been added to the SI (line 774):

The main ZIKV transmission model used a continuous flow of infectious individuals into the infectious compartment to better represent real introduction dynamics rather than a single introduction event at a single fixed point in time. This does not capture multiple separate introduction waves across multiple years.

2.10 "phulogenetic" should be "phylogenetic"

Thank you, this was an error and has been corrected.

2.13 Should your specificity for ZIKV not change through time if DENV-3 prevalence changes and impacts the false positive rate?

This would be an interesting parameter to vary with time but given our limited data we assumed that the sensitivity and specificity were fixed over time. The following is added to the SI (line 866):

We assumed that sensitivity and specificity were fixed over time which is unlikely to hold true in reality. However, we did not attempt to estimate time varying assay sensitivity and specificity because of the limited size of our data available.

2.14 Perhaps keep consistent and use of "I" instead of "we" (similar occurrences elsewhere in SI). You need to state the name of the first author (et al) as well as reference number (12).
2.15 Missing space between "study," and "we"
2.16 "clean-up" and "clean up" are used interchangeably. Please be consistent
2.17 Why have you used partial derivative notation for the ODEs?
2.18 "I" should be "I_Z" (equations 13/14).
2.19 Why is dengue not dependent on mosquitoes or seasonality? There is only a constant force of infection of beta_D?

These were all errors or inconsistencies and have been corrected. The error for 2.19 was in the equations in the manuscript but not in the model itself.

2.20 Mosquito population size: You don't say explicitly, but s_M, e_M, i_M are proportions. Your relative vector to host ratio should feature somewhere in R_0 though and at the moment it doesn't appear to be in your equations explicitly. I may have missed some non-dimensionalisation somewhere (and it might be contained as part of your lambdas/betas).

These are excellent points, however the mosquito population is no longer explicitly modelled and the relevant sections have been removed.

2.21 Although it won't make any ultimate difference to the final computed value of R_0, I think your R_ZM term shouldn't have the N in the dominator, and R_MZ should have it there (based on equations 8 and 14).

Apologies, I am unclear what denominator this was referring too. I suspect because they include "M" that these were relevant to the mosquito population and are no longer included.

2.22 Can you give a biological interpretation for beta_M and beta_Z in terms of bite rates, probability of infection and vector population size? This would help understand why your prior for beta_Z is between 0 and 1, whilst your beta_M prior can take any positive value.

The amended model has only one single transmission rate parameter, and the following has been added to the SI (line 829):

> *As detailed above, the force of infection $\lambda_Z(t)$ was time dependent according to seasonal forcing and the effect of a clean-up campaign in March 2014. The force of infection was relative to the baseline transmission rate β_Z, which is used as a single term to incorporate number of female mosquitoes, biting rate, probability of contact and probability of transmission. Without better entomological data from Fiji we used this simpler approach with a single transmission rate.*

2.23 What happens to people transitioning out of the R_Z box? Do they continue to remain uninfected and immune to both ZIKV and DENV-3? You talk about DENV-3 conferring immunity to ZIKV but not the other way around. Please provide some brief note on this, including why ZIKV immunity is not waning, whilst DENV-3 immunity is.

This should have been more explicit and the following has been added to the methods (line 297):

> *People leave the recovered compartment to reflect waning ZIKV antibodies over time, however they remained immune to ZIKV.*

Regarding waning ZIKV but not DENV-3, the following has been added to the SI (line 819):

I also fixed the duration of detectable ZIKV antibodies $\rho^{-1} = 400$ days based on our serological studies in French Polynesia and Fiji which found a decrease in ZIKV-specific antibodies 18 months after outbreaks in both locations (Henderson et al., 2020). This study also showed no evidence that DENV-3 antibodies waned over time so the parameter ρ was set to ∞ when running the model for DENV-3.

2.24 Model You don't explain C_Z(t) in the text around equation 11. I assume this counts the number of new infections at any given time? Later on (774) you use c_t=C_t-C_(t-1) – should this be C_Z(t)-C_Z(t-1)? This would make more sense to me.

You need to decide whether c_t is the number of new infections occurring or reported in the model. If it is occurring, your equation on line 774 makes sense, but equation 24 need to have an "r" in it for reporting. If it is reported (as per your text description on line 773) then c_t is not C_Z(t)-C_Z(t-1) but c_t=r(C_t-C_(t-1)).

I would have expected the serological data to be linked to R_Z(t) as this has the waning ZIKV antibodies component. Equation 24 uses C_j (which I assume is actually C_Z(j)?) which is the cumulative number of infected individuals rather than the ones with antibodies?

The following has been added to the SI (line 810):

The compartment C is introduced to capture the cumulative incidence of infections in the model.

Throughout the SI the C_t now captures the cumulative number of infections up to and including t, and c_t is the incident new infections at t. The relevant section now reads (line 854):

The full ZIKV transmission model was jointly fitted to case and serological data using adaptive MCMC with a Metropolis-Hastings algorithm. We assumed that cases were distributed according to a negative binomial distribution with mean equal to the number of incident infections reported in the model $c_t = r(C_t - C_{t-1})$, where r is the reporting proportion.

2.26 Rather than using arbitrary differences in DIC score which are very dependent on the data and set up of the log-likelihood function, can you compute the relative DIC score. Also please provide a reference for your selected DIC equation. There are many different variants and a brief justification of why you used this DIC would be helpful (although I am pleased you explicitly stated your calculation).

We appreciate your point about the relative DIC score. However, the new model fits to the various sensitivity analysis return such wide gaps in DIC score (line 936) that we feel this is sufficient justification that the chosen model fit is the best fit to data. However, we agree that a citation was necessary.

2.27 This SI has provided all the statistical outputs I would expect to see for the main model. Being critical – I would expect better mixing for the beta parameters after burn-in. I do

appreciate showing the two chains and diagnostic plots. Do you have trace plots for Models B and C?

We agree that the mixing was poor in our original model. Convergence has improved with the simplified model and fewer estimated parameters (Figures 16 and 17). Trace plots have also been included for all DENV-3 and ZIKV model fits used in this analysis and sensitivity analyses (Figures 18, 21-24).

2.28 I would find it very informative to see a map in SI of Fiji and central division

A map and description of Fiji has been added to the SI (Figure 5).

REVIEWER 3
I found the general approach interesting, especially the combination of case reports, serology and sequencing to draw inference on the outbreak dynamics. However, I have some concerns about the model structure as well as some of the conclusions and I think the manuscript requires substantial revisions before its validity and value can be properly assessed.

3.1 First of all, I am not convinced that the study does much to address the main question laid out in the introduction: why were zika case reports three orders of magnitude lower than the previous dengue outbreak? Although it is implied that confirmed cases are only a fraction of the total number of infections, I didn't see any explicit results or discussion of this issue. The SEIR model does not distinguish between infected individuals who may test positive or exhibit symptoms, and I had to dig into the appendix (line 774) to find the briefest mention of a reporting rate r, whose prior and posterior are nowhere to be seen. The simulations shown on Figure 3 suggest (but again that's not explained by the authors) that the Zika epidemic actually infected thousands of people (although the actual value is hard to read from a continuous prevalence curve). The panels in Figure 2 indicate that the effect of the timing of introduction can affect the epidemic size by roughly a factor 2 at most (less if you add the two successive epidemic peaks). I cannot reconcile these small fluctuations with the 1,000-fold difference between the case reports for dengue and Zika as shown on Figure 1 C. I can only assume that most of it is driven by different case to infection ratios, with timing of introduction only playing a marginal role. As a result, I'm not sure what can really be learned from this study.

We have added a paragraph to the discussion to summarise why we believe these outbreaks spread differently and were recorded differently (line 137):

> *We found that ZIKV was slightly less transmissible than DENV in the same population and that nearly all ZIKV infections were undetected, unlike the estimated reporting proportion during the DENV-3 epidemic. We show that if the ZIKV reporting proportion was equivalent to DENV-3 then the two epidemics would appear similar in overall magnitude (SI). However, this is insufficient to explain why ZIKV transmitted at a low level over multiple years, unlike DENV-3 which caused a large single-season epidemic. We have demonstrated that small changes to the introduction time can*

> *produce a diverse range of outbreak dynamics because of the strong seasonal forcing in ZIKV transmission in Fiji (Figure 3).*

We agree that the importance of the reporting differences was underplayed. We have also added this section to the results (line 88):

> *We also estimated a very small reporting rate for ZIKV from our model of 0.012% (95% CrI: 0.0069-0.025%). This implies that nearly all infections were not reported as cases and were either asymptomatic, not severe enough to seek medical attention, not referred for ZIKV tests by clinicians in Fiji or undetected ZIKV in tests. This low reporting rate is uncommon for arbovirus outbreaks in Fiji. We estimated a reporting rate of 25% (95% CrI: 18-38%) for DENV-3 during the 2013-14 epidemic. This discrepancy is the main cause of the diverse observed outbreaks in surveillance case data. However, it is insufficient to explain why ZIKV transmitted at a low level for multiple years.*

Figure 3 now includes the modelled DENV-3 infections to show that ZIKV could have produced a similar but slightly smaller epidemic (in terms of infections, not cases) if the introduction time changes by 1 month.

Finally, with these inclusions we have removed the previous model C, which fixed the reporting proportion at 0.1%. This sensitivity analysis was previously included to make a similar point about the low estimated reporting proportion for ZIKV but was clearly an inappropriate tool to do so.

3.2 I was surprised that a deterministic model was used given the very low number of cases. Even though I then realised that the numbers of infections were assumed to be much higher than the PCR-confirmed cases, there is still the potential for stochastic fadeouts between seasons. Deterministic models are known to generate clockwork epidemic cycles which can be very different from reality. A stochastic model would also be helpful for the introduction process, which involves very low numbers too.

We agree that this required further discussion in the manuscript. Please see point 2.8 above for detail but in brief, we prevented epidemic fadeout by explicitly requiring at least 1 infectious individual in the model. We found that our model estimated at least 100 infectious individuals between epidemic peaks. We also explicitly prevented infectious individuals being introduced to the model when the effective reproduction number was below 1.

3.3 I'm very confused by the way the DENV epidemic was modelled. The equations on page 34 do not match the diagram on Figure 8. I don't understand why individuals go from S_Z to S_D upon exposure to dengue (why not go to E_D?). In any case, the corresponding term is missing from equation 17 (or 18). Then the DENV infection rate appears to imply that \beta_D is the force of infection and is a constant over the epidemic, with no apparent effect of mosquito infection or seasonality, both of which are surprising. It's also a bit strange that, given all these and other approximations, the authors deemed it necessary to use an Erlang distribution (just with 2 sub-compartments) for the duration of cross-protection.

We have made amendments to the presentation of the model in the manuscript.

In the model diagram (Figure 10) there should not have been a connection between S_Z and S_D and this has been corrected. In the model people do not move from the Zika compartments to the Dengue compartments. However, a proportion (χ) of those infected with dengue are temporarily removed from the Zika compartments while they transition from S_D to T_{2D} and return to S_Z at rate ω. The model diagram and caption have been amended to show this more clearly.

Please see points 1.6-1.7 for details about the choice of Erlang distribution for cross-protection. Also please see point 2.19 regarding the typo in the model equations between S_D and E_D.

Upon review, we understand the confusion about the modelling of DENV-3 and have given this process greater clarity in the revised manuscript. In short, the DENV-3 outbreak was initially modelled using the same transmission model as ZIKV with an SEIR framework. This was done to calculate parameter values that would then be fixed for the ZIKV model. Subsequently, when the ZIKV outbreak was modelled the background DENV outbreak (S_D, E_D, I_D, R_D) was fixed with an $R_0 = 1.3$, of which a proportion (χ) would become temporally immune from ZIKV infection.

This was not clear in the original manuscript and we have added the following to the results section (line 76):

> *Before fitting to ZIKV data we initially fitted the same model to DENV-3 surveillance serological data from the 2013-14 epidemic (SI). From this analysis we estimated a higher but comparable median and 95% credible interval for R_0 for the 2013-14 DENV-3 epidemic (1.73; 95% CrI: 1.2-2.25).*

The following to the methods (line 312):

> *We initially fitted the transmission model to DENV-3 surveillance and serological data to estimate parameter value relating to the seasonal forcing and clean-up campaign in March 2014. These parameters were then fixed for the ZIKV transmission model. When modelling ZIKV, we fixed the parameters for the concurrent DENV-3 epidemic such that $R_0 = 1.3$ and a proportion of those infected would become temporally immune from ZIKV.*

And the following to the SI (line 840):

> *This model was used to separately fit both the DENV-3 and ZIKV epidemics to serological and surveillance data. We have described equations, compartments and parameters as ZIKV or DENV because ZIKV is the primary focus of the study. However, the same model was used to fit DENV-3 as the primary infection of interest, as was done to obtain estimates for parameters controlling the seasonal forcing and reduced transmission during the clean-up campaign.*

The results from the DENV-3 model fit are now included in the SI. Also, Table 2 (SI) has been expanded to show which parameters were estimated in the DENV-3 model run and which were estimated in the main ZIKV transmission model run, and estimated parameter values from the DENV-3 model run have been added to Table 3.

3.4 Given that simulations are run over several years, the lack of birth and death in the human population is an issue.

We agree that this was an important omission and now include demographic change in the model. Please see point 1.2 for further details.

3.5 Table 2 on page 36 shows a "test specificity" parameter with a prior set at U(0,1) which is quite surprising: was there no prior evidence? More surprisingly, there does not appear to be a sensitivity parameter. Given the very low number of PCR-positive cases reported, I would expect the sensitivity rate to be crucial for inference.

We have now included a sensitivity parameter in the model and we use informative priors on both the sensitivity and specificity based on previously reported values for this assay. The following is included in the discussion to summarise (line 195):

> *To reflect this, we included a parameter that measured the false positive proportion (1 minus the specificity) of the assay, which was estimated as 6.2% (95% CrI: 4.3%-8.5%) in the model fitting (SI). This may explain why there was some evidence of seroprevalence before our model estimated ZIKV had arrived in Fiji. Similarly, we estimated a sensitivity of 78% (95% CrI: 51-98%). Both are consistent with the previously reported assay sensitivity and specificity for ZIKV of 79.6% and 94.9% (Henderson et al., 2020).*

3.6 Lastly, I was confused by the way the interaction between ZIKV and DENV were assessed statistically. The Results section on page 8 appears to be testing the statistical support for an early introduction of ZIKV, which seems to be a very specific scenario. Did the authors seek to assess the statistical support for cross-immunity more generally?

We agree that this is not a direct test of cross-protection hypothesis. However, given the known pattern of recent DENV outbreaks in Fiji this would be the only plausible scenario where cross-protection would have affected observed ZIKV transmission dynamics and was not supported by the data.

REVIEWER COMMENTS

Reviewer #1 (Remarks to the Author):

The authors have fully addressed the comments. My pleasure to recommend its acceptance

Review
Interactions between timing and transmissibility explain diverse flavivirus dynamics in Fiji
NCOMMS-20-22353

Summary

The authors developed a model to understand what factors drive different transmission dynamics of DENV-3 and ZIKV in Fiji. They present nice use of data from different sources using a range of rigorous Bayesian methods and a mathematical model of transmission to explore introduction times, reporting proportions and waning seropositivity in people exposed to ZIKV. The figures are much clearer thanks to improvements since first submission. There are substantial improvements also to the model (simplifications were made which largely resolved my previous concerns) and the fitting (improvements in mixing).

I now have further technical amendments to clear up (predominantly) where changes to the model have been made and there are ambiguities in its description or missing information.

Specific comments:

Page	line	Comment
6	Fig 2	Please add a y-axis label for the grey "recoverds" in subfigure B<br>Why does this proportion go down? Waning immunity? If so I recommend your caption is modified to "population that had recovered *and were temporarily immune to* ZIKV infection"
7	76	At the first definition of R_0 please can you clarify this is the full cycle reproduction number (e.g. average new infections in hosts from an initial infected host). Even though you have now only implicitly included mosquitos it would be ideal to note this since there is inconsistency in vector-borne literature in the use of R_0 or R_0^2
16		Define HPD as "Highest Posterior Density"
30	704	"based on research from [13]" should have the name of the author + citation (i.e. Mordecai *et al.* [13]) rather than just the number.
32	718	I thought \beta_M had been removed? Likewise in equation 1, why are there references to i=Z and M? I assumed the need for mosquito-related equations disappeared with your host-only model?
33	Eq 3	What is "i" (subscript in control) is this a remnant from your Z and M versions?
37	Eq 5	I assume lambda_Z could be expressed without subscripts on the seasonal and control since you are not concerned with mosquitos explicitly?<br>Why is there no natural mortality when people are infected with dengue, but as soon as they return to ZIKV compartments there is?

		You don’t seem to have defined beta_D anywhere before or direct after these equations. Why is it not lambda_D rather than beta_D to have seasonality of dengue too?
37/38	Eqs	In 6,7,12 and 13 the formatting of your S*lambda*I/Ns and S*beta*I/Ns is confusing. It almost looks like the I_Z/I_Ds are superscripts. I recommend using a full display equation, or simply leaving I_Z, I_D and N as full size characters.
39	Eq 17	S should be S_Z. It would be worth mentioning this is the full cycle R number (as per my comment on R0 in the main text)
40	Table	This is the table of both fixed and fitted parameters, but some (fixed) are missing. It would be helpful to specify what the parameter values were for fixed ones. Some parameters including beta_D, nu, N, omega, mu, gamma_Z/D, and alpha_Z/D aren’t defined here and I don’t know what value they took without hunting around the text (beta_D is completely missing).
41	Equation after line 873	Equations should have numbers. The formatting is hard to read. I suggest using a display equation style.
45/46	Fig12/13	What is the prior for the reporting probability? I can’t see it, unlike all other fitted parameters where there is a red line. (I believe it is uniform, but missing from the plots)
49, 51, 55-58	Figs	Check the y-axes limits on your trace plots – sometimes not all chains are visible.

Reviewer #3 (Remarks to the Author):

Thank you for providing detailed responses to all the suggestions from the first round of reviews. I think the revised manuscript is much improved and has gained in clarity throughout. I am now satisfied with the validity of the approach and predictions but would like to make two further comments.
Firstly, I'm still unconvinced by the justification for the use of an Erlang distribution (which is really about the variance in the duration of immunity) but I believe it's a really minor point.
Secondly, while I agree that fitting a deterministic model to your data is valid, I think the range of predictions presented in Figure 3 (i.e. the effect of introduction time on epidemic sizes and attack rates) might look different with a stochastic model, given the value of R0 just above 1. It might not change the overall picture, but it would give additional insight into the probability of outbreaks and fadeouts. I'm not insisting you should add a stochastic model, and I appreciate that you have clearly acknowledged the limitations in the discussion, but I think it does limit the potential impact of your study.

Review received 12/01/2021

Response to reviewers

Thank you again for submitting your manuscript "Interactions between timing and transmissibility explain diverse flavivirus dynamics in Fiji" to Nature Communications. We have now received reports from 3 reviewers and, on the basis of their comments, we have decided to invite a revision of your work for further consideration in our journal. Your revision should address all the points raised by our reviewers (see their reports below). In particular, please address the technical comments made by Reviewer 2 (see attached document).

Thank you for the constructive and useful feedback. We are grateful for the thorough reviews and have addressed all remaining technical concerns.

Greater detail on these revisions are provided in the response to reviewer comments below.

REVIEWER 1
The authors have fully addressed the comments. My pleasure to recommend its acceptance.

REVIEWER 2:
The authors developed a model to understand what factors drive different transmission dynamics of DENV-3 and ZIKV in Fiji. They present nice use of data from different sources using a range of rigorous Bayesian methods and a mathematical model of transmission to explore introduction times, reporting proportions and waning seropositivity in people exposed to ZIKV. The figures are much clearer thanks to improvements since first submission. There are substantial improvements also to the model (simplifications were made which largely resolved my previous concerns) and the fitting (improvements in mixing).

I now have further technical amendments to clear up (predominantly) where changes to the model have been made and there are ambiguities in its description or missing information.

Thank you for raising these technical amendments. All suggested revisions have been implemented and a point-by-point guide to these revisions follows. The line numbers refer to positions in the "tracked changes" version of the revised manuscript.

2.1 Please add a y-axis label for the grey "recoverds" in subfigure B Why does this proportion go down? Waning immunity? If so I recommend your caption is modified to "population that had recovered and were temporarily immune to ZIKV infection"
The y-axis label in Figure 2B has been changed to include "Prop. recovered" and the figure caption has been changed as well in line with this recommendation.

2.2 At the first definition of R0 please can you clarify this is the full cycle reproduction number (e.g. average new infections in hosts from an initial infected host). Even though you have now only implicitly included mosquitos it would be ideal to note this since there is inconsistency in vector-borne literature in the use of R0 or R0
This clarification has been added on line 76.

2.3 Define HPD as "Highest Posterior Density"
Amended on line 326.

2.4 "based on research from 13" should have the name of the author + citation (i.e. Mordecai et al. 13) rather than just the number.
Amended on line 466.

2.5 I thought \beta_M had been removed? Likewise in equation 1, why are there references to i=Z and M? I assumed the need for mosquito-related equations disappeared with your host-only model?
2.6 What is "i" (subscript in control) is this a remnant from your Z and M versions?
2.7 I assume lambda_Z could be expressed without subscripts on the seasonal and control since you are not concerned with mosquitos explicitly?
Thank you for highlighting these inconsistencies. These were remnants from the former version of the model with a mosquito population and have been amended at Equations 1, 3 and 5.

2.8 Why is there no natural mortality when people are infected with dengue, but as soon as they return to ZIKV compartments there is?
2.9 You don't seem to have defined beta_D anywhere before or direct after these equations. Why is it not lambda_D rather than beta_D to have seasonality of dengue too?
A clarification has been added at line 821. When fitting the model to estimate ZIKV transmission dynamics we used a simpler model without seasonality or mortality for the simultaneous DENV-3 outbreak to simplify the model. In this situation, β_D, α_D and γ_D were fixed such that R_0 was 1.3 and a fixed proportion of the population were infected with DENV-3 between October 2013 and April 2014. This clarification has been added to Table 2 (page 40) as well (see point 2.12).

2.10 The formatting of your S*lambda*I/Ns and S*beta*I/Ns is confusing. It almost looks like the I_Z/I_Ds are superscripts. I recommend using a full display equation, or simply leaving I_Z, I_D and N as full size characters.
Amended in Equations 6,7, 12 and 13.

2.11 S should be S_Z. It would be worth mentioning this is the full cycle R number (as per my comment on R0 in the main text)
Amended on line 854 and Equation 17.

2.12 This is the table of both fixed and fitted parameters, but some (fixed) are missing. It would be helpful to specify what the parameter values were for fixed ones. Some parameters including beta_D, nu, N, omega, mu, gamma_Z/D, and alpha_Z/D aren't defined here and I don't know what value they took without hunting around the text (beta_D is completely missing).
Table 2 (page 40) has been expanded to include all parameters used in both the DENV-3 and ZIKV model fitting processes.

2.13 Equations should have numbers. The formatting is hard to read. I suggest using a display equation style.

Amended at Equation 18.

2.14 What is the prior for the reporting probability? I can't see it, unlike all other fitted parameters where there is a red line. (I believe it is uniform, but missing from the plots)
This is a limitation with the plotting function used. The following clarification has been added to the Figure 13 caption "A uniform prior was used for the reporting proportion but is not shown in the plot".

2.15 Check the y-axes limits on your trace plots – sometimes not all chains are visible.
This is a limitation with the plotting function used. The following clarification has been added to the Figure captions "A condensed plotting area is shown here to focus on the converged region of the chains. Hence sometimes chains may not be visible; this is a sign of poor convergence".

REVIEWER 3
Thank you for providing detailed responses to all the suggestions from the first round of reviews. I think the revised manuscript is much improved and has gained in clarity throughout. I am now satisfied with the validity of the approach and predictions but would like to make two further comments.

Firstly, I'm still unconvinced by the justification for the use of an Erlang distribution (which is really about the variance in the duration of immunity) but I believe it's a really minor point.
We agree that a wider range of distributions could in theory be explored, but our decision of distribution is extremely unlikely to affect our conclusions, because there is strong evidence that ZIKV was introduced after the DENV-3 2013/14 epidemic therefore the variance in duration of any cross-protection from DENV-3 will not affect our main findings.

Secondly, while I agree that fitting a deterministic model to your data is valid, I think the range of predictions presented in Figure 3 (i.e. the effect of introduction time on epidemic sizes and attack rates) might look different with a stochastic model, given the value of R0 just above 1. It might not change the overall picture, but it would give additional insight into the probability of outbreaks and fadeouts. I'm not insisting you should add a stochastic model, and I appreciate that you have clearly acknowledged the limitations in the discussion, but I think it does limit the potential impact of your study.
We agree that using a stochastic model could provide further insight into arbovirus emergence and fadeout dynamics in Fiji – such models would be particularly interesting for exploring factors like critical community size in smaller Pacific Island nations. However, we believe that the deterministic model presented in our manuscript is sufficient to address our main study aim, which focuses on a period of sustained multi-wave transmission within the relatively large Fiji population, and the effect of accumulated immunity and seasonality on dynamics during this period.
